# Computational analysis of the amino acid interactions that promote or decrease protein solubility

**DOI:** 10.1038/s41598-018-32988-w

**Published:** 2018-10-02

**Authors:** Qingzhen Hou, Raphaël Bourgeas, Fabrizio Pucci, Marianne Rooman

**Affiliations:** Department of BioModeling BioInformatics & BioProcesses, Université Libre de Bruxelles, Brussels, 1050 Belgium

## Abstract

The solubility of globular proteins is a basic biophysical property that is usually a prerequisite for their functioning. In this study, we probed the solubility of globular proteins with the help of the statistical potential formalism, in view of objectifying the connection of solubility with structural and energetic properties and of the solubility-dependence of specific amino acid interactions. We started by setting up two independent datasets containing either soluble or aggregation-prone proteins with known structures. From these two datasets, we computed solubility-dependent distance potentials that are by construction biased towards the solubility of the proteins from which they are derived. Their analysis showed the clear preference of amino acid interactions such as Lys-containing salt bridges and aliphatic interactions to promote protein solubility, whereas others such as aromatic, His-*π*, cation-*π*, amino-*π* and anion-*π* interactions rather tend to reduce it. These results indicate that interactions involving delocalized *π*-electrons favor aggregation, unlike those involving no (or few) dispersion forces. Furthermore, using our potentials derived from either highly or weakly soluble proteins to compute protein folding free energies, we found that the difference between these two energies correlates better with solubility than other properties analyzed before such as protein length, isoelectric point and aliphatic index. This is, to the best of our knowledge, the first comprehensive in silico study of the impact of residue-residue interactions on protein solubility properties.The results of this analysis provide new insights that will facilitate future rational protein design applications aimed at modulating the solubility of targeted proteins.

## Introduction

Solubility is a fundamental and complex biophysical property of globular proteins, which is often crucial for their correct functioning^1,2^. It is intimately connected with the stability of the three-dimensional (3D) protein structure and strongly depends on environmental quantities such as the pH, the temperature, the buffer type and the protein concentration.

Solubility problems manifest themselves through different physical behaviors. The simplest one consists of the irreversible formation of native-state protein precipitants when the protein concentration overpasses the solubility limit; note that this limit depends on the environmental conditions. The picture gets more complicated when the aggregated or precipitated form includes not only native structures but also misfolded, partially folded and unfolded conformations. The formation of highly ordered aggregates such as amyloid fibrils from misfolded conformations constitutes the pathological characteristics of a large variety of disease conditions such as the neurodegenerative Alzheimer and Parkinson diseases. In these cases, the deposition of the *β*-amyloid and *α*-synuclein aggregates, respectively, in the patient’s brain prevents the normal functioning of neurons^2–5^.

Lack of solubility is frequently a major bottleneck in high-throughput structural genomic studies as well as in industrial applications requiring high-concentration production of recombinant proteins, such as monoclonal antibody solutions for pharmaceutical applications. In these processes, the formation of amorphous inclusion bodies from the aggregation of different (denatured and partially folded) conformations limits the biological activity of the product and makes necessary to implement complex solubilization and refolding procedures in order to recover the bioactive forms^6–11^.

The understanding of the mechanisms that modulate protein solubility is highly challenging due to their dependence on many intrinsic and extrinsic factors. Unraveling these complex relationships and the connection between the 3D structural properties and the solubility is a crucial objective for many academic and biotechnological applications. Despite the research devoted to these problems in the last 20 years and some important advances, the precise identification of the amino acid interactions and structural characteristics that lead to soluble or aggregated states and their physical interpretation remain elusive.

An early study^12^ showed that the solubility of proteins overexpressed in *Escherichia coli* is anti-correlated with the total number of residues. Regarding the contribution of specific amino acids to protein solubility, the favorable role of the negatively charged aspartic and glutamic acids was observed^13^. This trend was confirmed by other studies^14–16^. In contrast, weakly soluble protein expression appears to be correlated with large, positively charged, surface patches^17^. Note that recent studies demonstrated that arginines lead to aggregation, but not lysines^17,18^, probably because the Arg side chain is more prone to inter-protein interactions. Finally, a series of investigations point out that aromatic-rich proteins tend to be less soluble than aromatic-depleted ones^16,19^.

Many of these properties, combined with sequence features such as the aliphatic index, the secondary structure propensities and/or the amino acid composition, have been employed by computational approaches to predict the soluble nature of target proteins^15,19–22^. Although these methods reach good performances and are thus quite useful, their sequence-based nature linked to the fact that they employ “black box” machine learning approaches, fails in providing comprehensive biophysical insights into protein solubility.

In this paper, we used knowledge-based mean force potentials derived from datasets of protein structures of known solubility to get a clearer picture of the mechanisms that drive protein solubility. In particular, we focused on the solubility dependence of all possible amino acid pair interactions, with the aim of understanding which and why some of them are more favorable in soluble than in weakly soluble proteins and *vice versa*. We also tested the ability of our new potentials to discriminate between soluble and aggregation-prone proteins, on different datasets and with different solubility definitions. The comprehension gained from such studies is of utmost importance for the rational design of proteins with increased solubility, a challenging goal in protein engineering. Indeed, it saves costly, time-consuming, wet lab experiments that are needed to reduce unwanted aggregate formation and increase solubility^14,23,24^.

## Methods

### Protein structure and solubility dataset

To investigate the relation between protein structure, energy properties and solubility, we constructed a dataset of high-resolution X-ray structures with known solubility value. The starting point was the eSOL database^16^ that contains aggregation propensities of about 70% of the entire proteome of the *E*. *coli* K-12 strain synthesized with the PURE system^25^, an *in vitro* reconstituted and chaperone-free translation system. For each protein, the solubility ${\mathscr{S}}$ (in %) was experimentally determined as the ratio between the supernatant protein fraction obtained after centrifugation of the translation mixture, and the total uncentrifuged protein fraction.

To map the gene accession IDs associated with the eSOL entries onto the corresponding 3D structures in the Protein Data Bank (PDB)^26^, we used the EcoGene server^27^, a functional and structural annotation database of *E*. *coli*. We selected only the PDB structures that have a sequence identity of 100% with the associated EcoGene entries, as evaluated with the sequence alignment software BLAST^28^. The protein-culling server PISCES^29^ was then used to further refine the structure dataset and avoid biases due to the inclusion of proteins of similar sequences. We chose a threshold value of 25% on the pairwise sequence identity and a structure resolution of 2.5 Å at most. Transmembrane proteins were also filtered out.

The resulting ${{\mathscr{D}}}^{{\rm{tot}}}$ set is composed of 412 proteins with experimental structure and solubility. To investigate how protein structural properties are related to solubility, we divided this dataset in two subsets with an equal number of proteins. The first set, called ${{\mathscr{D}}}^{{\rm{sol}}}$, contains all structures with solubility ${\mathscr{S}}\ge 64 \%$, while the ${{\mathscr{D}}}^{{\rm{insol}}}$ dataset is composed of aggregation-prone proteins with ${\mathscr{S}} < 64 \%$. The list of proteins in these sets and some of their characteristics are given in Table S1, the distribution of soluble and weakly soluble proteins in Fig. S1, and the relative frequency of the twenty amino acids in the two datasets in Fig. S2 of Supplementary Information.

### Standard statistical residue-residue potentials

Knowledge-based statistical potentials were used to describe the interaction strength between two interacting residues. These potentials of mean force^30–33^ are widely used in a large variety of applications, from protein structure prediction to the analysis of the impact of mutations on protein stability. They are derived from the frequency of observation of associations of specific sequence-structure elements in a dataset of experimental 3D protein structures using the inverse Boltzmann law.

In this paper we focused on distance potentials, where the structure elements are the distances *d* between the side chain geometric centers of two amino acids. The sequence elements are amino acid types *s* and *s*′. The energy associated to a sequence-structure association $(s,s^{\prime} ,d)$ can be evaluated as^31,33^:

1 $${\rm{\Delta }}W(s,s^{\prime} )=-\,{k}_{B}T\,\mathrm{ln}\,\frac{P(s,s^{\prime} ,d)}{P(s,s^{\prime} )P(d)}$$

where *k*_*B*_ is the Boltzmann constant and *T* the absolute temperature. $P(s,s^{\prime} ,d)$ is the probability of observation of two amino acid types *s* and *s*′ at the spatial distance *d*, $P(s,s^{\prime} )$ the probability of these two amino acid types at any distance, and *P*(*d*) the probability of any types of amino acids at the distance *d*. These probabilities are estimated from the relative frequencies *F* of observation of sequence-structure elements in a dataset of 3D protein structures, which are in turn derived from the number of occurrences *n* of these elements as:

2 $${\rm{\Delta }}W(s,s^{\prime} ,d)\cong -\,{k}_{B}T\,\mathrm{ln}\,\frac{F(s,s^{\prime} ,d)}{F(s,s^{\prime} )F(d)}=-\,{k}_{B}T\,\mathrm{ln}\,\frac{n\,{n}_{ss^{\prime} d}}{{n}_{ss^{\prime} }\,{n}_{d}}$$

where *n* is the total number of amino acid pairs. The distances *d*, between 3 and 10 Å, were divided into 35 bins of 0.2 Å width; the last bin contains all distances larger than 10 Å. The discretized *d* values correspond to the middle value of each bin. The frequencies were computed separately according to the separation along the sequence of the two amino acids *s* and *s*′. More precisely, if *s* and *s*′ are at positions *i* and *j* along the sequence, respectively, a separate potential is computed for each value of $1 < |i-j|\le 8$, to take into account the effect of the protein chain. For $|i-j| > 8$, where the effect of the chain can be considered as insignificant, all the frequencies are mixed into a single potential.

### Solubility-dependent statistical potentials

A commonly alleged drawback of the statistical potential formalism defined in Eq. (2) is their bias towards the protein structure dataset from which they are derived. However, this drawback can be turned into an asset if these biases are utilized to better describe specific properties of the dataset. The temperature dependence of the amino acid interactions has been extensively analyzed using this technique in our earlier works^34–36^.

Here we used this strategy to deepen the analysis of protein solubility at the molecular level. The central idea is that the potentials obtained from the complete dataset ${{\mathscr{D}}}^{{\rm{tot}}}$ and from the datasets ${{\mathscr{D}}}^{{\rm{sol}}}$ and ${{\mathscr{D}}}^{{\rm{insol}}}$, which only contain protein structures with solubility values in a certain range, reflect the properties of the ensemble from which they are derived.

We defined three types of statistical potentials. The first, referred to as soluble protein potentials, are obtained from the dataset of soluble proteins ${{\mathscr{D}}}^{{\rm{sol}}}$ and the full set ${{\mathscr{D}}}^{{\rm{tot}}}$^34^:

3 $${\rm{\Delta }}{W}^{{\rm{sol}}}(s,s^{\prime} ,d)\cong -\,{k}_{B}T\,\mathrm{ln}\,\frac{F(s,s^{\prime} ,d,{{\mathscr{D}}}^{sol})}{F(s,s^{\prime} ,{{\mathscr{D}}}^{tot})F(d,{{\mathscr{D}}}^{sol})}$$

where $F(s,s^{\prime} ,d,{{\mathscr{D}}}^{{\rm{sol}}})$ and $F(d,{{\mathscr{D}}}^{{\rm{sol}}})$ are observation frequencies computed in the ${{\mathscr{D}}}^{{\rm{sol}}}$ subset, while $F(s,s^{\prime} ,{{\mathscr{D}}}^{{\rm{tot}}})$ are frequencies from the ${{\mathscr{D}}}^{{\rm{tot}}}$ set. In an analogous way, the second type of potentials, called for simplicity “insoluble” protein potentials, are derived from the ${{\mathscr{D}}}^{{\rm{insol}}}$ set of weakly soluble proteins and the total set ${{\mathscr{D}}}^{{\rm{tot}}}$:

4 $${\rm{\Delta }}{W}^{{\rm{insol}}}(s,s^{\prime} ,d)\cong -\,{k}_{B}T\,\mathrm{ln}\,\frac{F(s,s^{\prime} ,d,{{\mathscr{D}}}^{{\rm{insol}}})}{F(s,s^{\prime} ,{{\mathscr{D}}}^{{\rm{tot}}})F(d,{{\mathscr{D}}}^{{\rm{insol}}})}$$

The last potentials, referred to as total potentials, are computed from the complete set ${{\mathscr{D}}}^{{\rm{tot}}}$ only:

5 $${\rm{\Delta }}{W}^{{\rm{tot}}}(s,s^{\prime} ,d)\cong -\,{k}_{B}T\,\mathrm{ln}\,\frac{F(s,s^{\prime} ,d,{{\mathscr{D}}}^{{\rm{tot}}})}{F(s,s^{\prime} ,{{\mathscr{D}}}^{{\rm{tot}}})F(d,{{\mathscr{D}}}^{{\rm{tot}}})}$$

### Coping with finite-size dataset effect

When estimating the probabilities in eq. (1) in terms of frequencies to obtain Eq. (2), the underlying assumption is that the number of protein structures contained in the dataset is large enough to yield statistically significant values. While this is in general a reasonable hypothesis for standard statistical potentials, which are derived from thousands of structures, it is less so for the potentials constructed here, since there are only a few hundreds of protein structures with experimentally characterized solubility. The relative smallness of the ${{\mathscr{D}}}^{{\rm{sol}}}$ and ${{\mathscr{D}}}^{{\rm{insol}}}$ sets is thus likely to introduce some distortions. To cope with these problems and get smooth and statistically significant potentials, we introduced two additional layers of computation.

The first layer consists in considering only the distance bins *d* that contain a sufficient number of occurrences. We chose the threshold value on *n*_*ss*′*d*_ equal to 10. If this value is not reached, the potentials are set to zero. Eq. (2) thus becomes:

6 $$\begin{array}{llll}{\rm{\Delta }}W(s,s^{\prime} ,d) & = & -{k}_{B}T\,\mathrm{ln}\,\frac{n\,{n}_{ss^{\prime} d}}{{n}_{ss^{\prime} }\,{n}_{d}} & {\rm{if}}\,{n}_{ss^{\prime} d} > 10\\ {\rm{\Delta }}W(s,s^{\prime} ,d) & = & 0 & {\rm{otherwise}}\end{array}$$

The second layer is dedicated to achieving a smoother potential behavior through a smoothing procedure that consists in replacing the number of occurrences in a bin $(s,s^{\prime} ,d)$ with the weighted sum of the occurrences of the four neighborhood bins as:

7 $${\hat{n}}_{ss^{\prime} d}=\frac{1}{{\alpha }^{2}}{n}_{ss^{\prime} (d-2b)}+\frac{1}{\alpha }{n}_{ss^{\prime} (d-b)}+{n}_{ss^{\prime} d}+\frac{1}{\alpha }{n}_{ss^{\prime} (d+b)}+\frac{1}{{\alpha }^{2}}{n}_{ss^{\prime} (d+2b)}$$

where *α* is a constant larger than one, which we fixed here to 4/3, and *b* is the width of the distance bin, equal here to 0.2 Å. The four bins $d\pm b$ and $d\pm 2b$ correspond to the four bins that are the closest from the central bin *d*. The number of occurrences ${\hat{n}}_{ss}$ and ${\hat{n}}_{d}$ are obtained from ${\hat{n}}_{ssd}$ by summing over all distances and amino acid types, respectively.

### Statistical significance analysis

To quantitatively determine whether the differences between soluble and insoluble potentials are statistically significant or due to random fluctuations, we computed two quantities: the mean ${\mathcal M}$ difference between the two potentials, summed over all *N*_*d*_ distances bins:

8 $${ {\mathcal M} }_{ss^{\prime} }=\frac{1}{{N}_{d}}\,\sum _{d=1}^{{N}_{d}}\,({\rm{\Delta }}{W}^{{\rm{sol}}}(s,s^{\prime} ,d)-{\rm{\Delta }}{W}^{{\rm{insol}}}(s,s^{\prime} ,d))$$

and the variance ${\mathscr{V}}$ of these potentials:

9 $${{\mathscr{V}}}_{ss^{\prime} }=\frac{1}{{N}_{d}}\,\sum _{d=1}^{{N}_{d}}\,{({\rm{\Delta }}{W}^{{\rm{sol}}}(s,s^{\prime} ,d)-{\rm{\Delta }}{W}^{{\rm{insol}}}(s,s^{\prime} ,d))}^{2}$$

To test the significance of the differences between soluble and insoluble potentials for a given residue pair (*s*, *s*′), we compared $|{ {\mathcal M} }_{ss^{\prime} }|$ and ${{\mathscr{V}}}_{ss^{\prime} }$ with the analogous quantities computed on sets obtained by randomly separating ${{\mathscr{D}}}^{{\rm{tot}}}$ into two subsets with an equal number of proteins. This random shuffling and ${\mathcal M}$ and ${\mathscr{V}}$ computations were repeated 100 times. If the $|{ {\mathcal M} }_{ss^{\prime} }|$ and/or ${{\mathscr{V}}}_{ss^{\prime} }$ values computed from the datasets ${{\mathscr{D}}}^{{\rm{sol}}}$ and ${{\mathscr{D}}}^{{\rm{insol}}}$ are higher than 95% of those computed from the randomized datasets, the interaction (*s*, *s*′) was considered to differ significantly between soluble and aggregation-prone proteins. We actually used two statistical significance criteria: a stricter one in which the fraction of randomly obtained $|{ {\mathcal M} }_{ss^{\prime} }|$ and ${{\mathscr{V}}}_{ss^{\prime} }$ values that are smaller than the actual $|{ {\mathcal M} }_{ss^{\prime} }|$ and ${{\mathscr{V}}}_{ss^{\prime} }$ values, denoted Sig${ {\mathcal M} }_{ss^{\prime} }$ and Sig${{\mathscr{V}}}_{ss^{\prime} }$, are both larger than 0.95, and a relaxed criterion in which Sig${ {\mathcal M} }_{ss^{\prime} }\ge 0.95$ or Sig${{\mathscr{V}}}_{ss^{\prime} }\ge 0.95$.

### Solubility-dependent protein folding free energy

Three types of folding free energies were computed for proteins represented by their sequence *S* and 3D conformation *C*, using the three potentials derived from the soluble, insoluble and total protein datasets, as defined in eqs (3, 4 and 5):

10 $$\begin{array}{rcl}{\rm{\Delta }}{W}_{S,C}^{{\rm{sol}}} & = & \sum _{i=1}^{N}\,\sum _{j=i+2}^{N}\,{\rm{\Delta }}W({s}_{i},{s}_{j}^{\prime} ,d,{{\mathscr{D}}}^{sol})\\ {\rm{\Delta }}{W}_{S,C}^{{\rm{insol}}} & = & \sum _{i=1}^{N}\,\sum _{j=i+2}^{N}\,{\rm{\Delta }}W({s}_{i},{s}_{j}^{\prime} ,d,{{\mathscr{D}}}^{insol})\\ {\rm{\Delta }}{W}_{S,C}^{{\rm{tot}}} & = & \sum _{i=1}^{N}\,\sum _{j=i+2}^{N}\,{\rm{\Delta }}W({s}_{i},{s}_{j}^{\prime} ,d,{{\mathscr{D}}}^{tot})\end{array}$$

where *s*_*i*_ and ${s}_{j}^{\prime}$ are two amino acid types at positions *i* and *j* along the sequence, respectively; *N* is the sequence length. To avoid any overfitting, the folding free energies were computed using a leave-one-out cross validation strategy, consisting of removing the target protein $(\bar{S},\bar{C})$ from all the datasets ${{\mathscr{D}}}^{{\rm{sol}}}$, ${{\mathscr{D}}}^{{\rm{insol}}}$ and ${{\mathscr{D}}}^{{\rm{tot}}}$ when computing its folding free energies ${\rm{\Delta }}{W}_{\bar{S},\bar{C}}^{{\rm{sol}}}$, ${\rm{\Delta }}{W}_{\bar{S},\bar{C}}^{{\rm{insol}}}$ and ${\rm{\Delta }}{W}_{\bar{S},\bar{C}}^{{\rm{tot}}}$. Note that this cross validation procedure is very strict, since the datasets contain, by construction, no proteins with more than 25% sequence identity with any target $(\bar{S},\bar{C})$.

We also computed the soluble and insoluble folding free energy difference:

11 $${\rm{\Delta }}{W}_{S,C}^{\mathrm{insol}-\mathrm{sol}}={\rm{\Delta }}{W}_{S,C}^{{\rm{insol}}}-{\rm{\Delta }}{W}_{S,C}^{{\rm{sol}}}$$

It was used to estimate protein solubility.

## Results and Discussion

**Figure 1:**
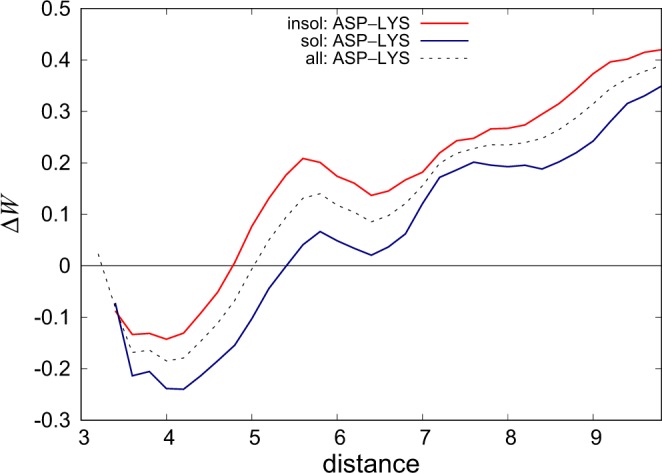
The folding free energy contribution ${\rm{\Delta }}W({\rm{Asp}},\,{\rm{Lys}},\,d)$ of the salt bridge interaction Asp-Lys differs according to whether the potentials are derived from soluble or weakly soluble proteins. The energies are in kcal/mol, the distance *d* (in Å) is computed between residue side chain centroids, and the residues are separated by at least 8 residues along the chain. Distance bins containing ten occurrences or less are not drawn (see Eq. (6)).

We derived both classical and solubility-dependent statistical distance potentials from the three sets ${{\mathscr{D}}}^{{\rm{sol}}}$, ${{\mathscr{D}}}^{{\rm{insol}}}$ and ${{\mathscr{D}}}^{{\rm{tot}}}$ containing proteins with different solubility values, with the aim of quantifying the contribution of amino acid pair interactions to protein solubility. These novel potentials ${\rm{\Delta }}{W}^{{\rm{sol}}}$, ${\rm{\Delta }}{W}^{{\rm{insol}}}$ and ${\rm{\Delta }}{W}^{{\rm{tot}}}$ were computed and analyzed for all 210 residue-residue pairs. For each of them, we computed the folding free energy profiles as a function of the distance *d* between the residues, compared the profiles obtained with the three potentials, and identified the residue pairs for which the profiles differ significantly. In this way, we were able to highlight the interactions that contribute more strongly than the others to the increase or decrease of protein solubility. A first striking result is that the soluble and insoluble folding free energy profiles obtained with ${\rm{\Delta }}{W}^{{\rm{sol}}}$ and ${\rm{\Delta }}{W}^{{\rm{insol}}}$ differ for a large number of residue pairs, with the ${\rm{\Delta }}{W}^{{\rm{tot}}}$ profiles always in between these two extremes. An example is shown in Fig. 1 for lysine-aspartic acid interacting pairs. The interaction energy presents a clear minimum when the residue side chain centers are about 3–4 Å apart, which corresponds to a salt bridge interaction. Clearly, this interaction appears more favorable in soluble proteins than in aggregation-prone proteins, which means that they contribute more strongly to the stability of the native structure of soluble proteins.

The whole set of energy profiles computed with the three types of potentials, for the 210 residue pairs, is shown in Fig. S3 of Supplementary Information. Tables 1 and 2 contain the insolubilizing and solubilizing pair interactions, respectively, which are estimated as statistically significant on the basis of both ${\mathcal M}$ and ${\mathscr{V}}$, and Tables S2 and S3 those that are significant on the basis of ${\mathcal M}$ or ${\mathscr{V}}$.

**Table 1:** Insolubilizing residue-residue interactions, defined by ${\mathcal M} \, < \,0$ and the strict significance criteria requiring both $| {\mathcal M} |$ and ${\mathscr{V}}$ values to be higher than 95% of the equivalent quantities computed from randomly shuffled datasets (Sig${\mathcal M}$ and Sig${\mathscr{V}}\ge 0.95$).

Interactions	Residue pairs	${\boldsymbol{ {\mathcal M} }}$	Sig${\boldsymbol{ {\mathcal M} }}$	$\pmb{\mathscr{V}}$	Sig$\pmb{\mathscr{V}}$
*π*-*π*	TRP-TRP	−0.412	0.99	0.181	0.99
	TRP-PHE	−0.207	1	0.052	1
	TYR-TRP	−0.177	1	0.038	0.99
	TYR-PHE	−0.124	0.97	0.019	0.99
His-*π*	HIS-TYR	−0.155	1	0.038	0.99
	HIS-TRP	−0.191	0.99	0.063	1
	HIS-PHE	−0.122	0.96	0.022	0.95
Cation-*π*	ARG-TRP	−0.238	1	0.074	1
	ARG-PHE	−0.120	0.99	0.017	0.99
	ARG-TYR	−0.101	0.98	0.017	0.98
	LYS -TRP	−0.162	0.97	0.068	0.98
Amino-*π*	GLN-TRP	−0.359	1	0.135	1
	GLN-PHE	−0.128	1	0.028	1
	ASN-PHE	−0.140	1	0.024	0.99
	ASN-TRP	−0.183	1	0.044	0.98
	GLN-TYR	−0.141	0.99	0.024	0.95
Anion-*π*	ASP-TRP	−0.211	1	0.049	1
Aromatic-containing	TRP-SER	−0.294	1	0.104	1
	PHE-CYS	−0.232	1	0.062	1
	TRP-ALA	−0.206	1	0.048	1
	TRP-PRO	−0.205	1	0.045	1
	TYR-SER	−0.129	1	0.021	1
	TRP-LEU	−0.192	1	0.037	1
	TRP-GLY	−0.153	0.99	0.033	0.98
	TRP-CYS	−0.267	0.99	0.076	0.97
	TYR-GLY	−0.109	0.98	0.021	0.97
	TRP-ILE	−0.114	0.97	0.024	0.98
His-containing	HIS-ALA	−0.108	1	0.016	0.98
	HIS-PRO	−0.124	0.99	0.021	0.97
	HIS-LEU	−0.110	0.97	0.027	0.99
Arg-containing	ARG-SER	−0.152	1	0.025	1
	ARG-ARG	−0.184	0.99	0.036	0.99
	ARG-PRO	−0.128	0.99	0.030	0.99
	ARG-LEU	−0.084	0.99	0.008	0.96
	ARG-CYS	−0.230	0.98	0.062	0.98
	ARG-GLN	−0.166	1	0.033	1
	ARG-ASN	−0.120	0.99	0.023	1
Asn/Gln-containing	ASN-GLN	−0.158	0.99	0.032	0.99
	GLN-CYS	−0.152	0.95	0.051	1
Miscellaneous	LEU-CYS	−0.195	1	0.050	1
	LEU-SER	−0.074	0.97	0.010	0.97
	SER-SER	−0.109	0.96	0.019	0.95

**Table 2:** Solubilizing residue-residue interactions, defined by ${\mathcal M} \, > \,0$ and the strict significance criteria requiring both $| {\mathcal M} |$ and ${\mathscr{V}}$ values to be higher than 95% of the equivalent quantities computed from randomly shuffled datasets (Sig${\mathcal M}$ and Sig${\mathscr{V}}\ge 0.95$).

Interactions	Residue pairs	${\boldsymbol{ {\mathcal M} }}$	Sig${\boldsymbol{ {\mathcal M} }}$	$\pmb{\mathscr{V}}$	Sig$\pmb{\mathscr{V}}$
Lys-salt bridges	LYS-GLU	0.115	1	0.017	1
	LYS-ASP	0.105	0.97	0.013	0.96
Aliphatic-aliphatic	VAL-VAL	0.156	1	0.025	1
	ILE-ILE	0.125	1	0.018	1
	VAL-ILE	0.096	1	0.010	1
	GLY-VAL	0.114	1	0.015	1
	ILE-ALA	0.072	1	0.006	0.97
	LEU-ILE	0.064	0.99	0.007	1
	LEU-VAL	0.058	0.99	0.004	0.96
	GLY-GLY	0.113	0.98	0.014	0.96
Aliphatic-containing	ILE-LYS	0.134	1	0.026	1
	VAL-GLU	0.120	1	0.017	1
	VAL-THR	0.086	1	0.010	0.99
	GLY-ASP	0.114	1	0.017	0.99
	ILE-THR	0.080	1	0.008	0.97
	GLY-THR	0.093	0.99	0.015	0.99
	GLY-GLU	0.105	0.99	0.012	0.96
	ILE-GLU	0.089	0.99	0.011	0.95
	ALA-LYS	0.095	0.98	0.013	0.97
	VAL-PRO	0.068	0.95	0.008	0.98
	VAL-LYS	0.097	0.95	0.014	0.98
Miscellaneous	GLU-THR	0.153	1	0.032	1

In the next two subsections, the pair interactions that contribute most to the increase or decrease of protein solubility are extensively discussed. We grouped and analyzed together the residue pairs that share similar biophysical characteristics, in order to illustrate the solubility dependence of amino acid interactions, provide an overview of their contribution to protein solubility and unravel the underlying physical principles.

### Interactions that decrease the solubility

There are 42 residue-residue interactions which are more favorable in aggregation-prone proteins than in soluble proteins (${{\rm{Sig}}}_{ {\mathcal M} }\ge 0.95$ and ${{\rm{Sig}}}_{{\mathscr{V}}}\ge 0.95$) (Table 1), and 58 if the less strict statistical significance criterion is used (${{\rm{Sig}}}_{ {\mathcal M} }\ge 0.95$ or ${{\rm{Sig}}}_{{\mathscr{V}}}\ge 0.95$) (Table S2).

The first result that falls up when looking at these tables is that almost all insolubilizing interactions involve side chain moieties with delocalized *π*-electrons^37^. Indeed, many involve the aromatic residues Phe, Tyr and Trp, as well as His which is also aromatic although usually considered separately as it carries a positive charge under some conditions. These aromatic residues have *π*-electrons that are delocalized below and above the plane of the aromatic moiety. The other residues that are overrepresented among desolubilizing interactions are: arginine, whose side chain carries a guanidinium cation that has three resonance forms with the positive charge delocalized on three N atoms; aspartic and glutamic acids, which possess a carboxylic acid anion with two resonating forms and the negative charge delocalized on the two O atoms; asparagine and glutamine, whose side chain has a neutral amide group with two resonating forms, one having a partial positive charge on the NH_2_ group and a partial negative charge on the O atom. We detail in what follows the different types of insolubilizing interactions that satisfied our statistical significance tests.

#### Aromatic-aromatic or *π*-*π* interactions

The interaction between two non-charged amino acids with aromatic side chains (Phe, Trp, Tyr) are known to be essential for the stabilization of protein structure and protein complexes^38^. Their attraction occurs through the interaction between the aromatic rings that contain delocalized *π*-electrons. Their interaction geometries are classified in three types, namely T-shaped, face-to-face and off-stacked^38^. Two kinds of physical forces stabilize these conformations, the electrostatic force that comes from the interaction between the quadrupole moments of the aromatic rings, and the London dispersion force that results from the *π*-electron delocalization on the ring and the overlap between the *π*-orbitals of the two aromatic moieties. The face-to-face geometry is mainly stabilized by the London force, which tends to compensate the electrostatic contribution that is unfavorable in this case. In the off-stacked and T-shaped conformations, both the electrostatic and dispersion contributions are stabilizing, which makes them usually more favorable and thus more frequent than face-to-face conformations. Note that the most favorable geometries also depend on the extracyclic atoms and thus on the type of amino acid.

The distance-dependent profiles of the six aromatic-aromatic interaction potentials (Phe-Phe, Phe-Tyr, Phe-Trp, Tyr-Tyr, Tyr-Trp, Trp-Trp) are clearly well separated according to whether they are computed from the soluble or insoluble protein potentials ${\rm{\Delta }}{W}^{{\rm{sol}}}$ and ${\rm{\Delta }}{W}^{{\rm{insol}}}$, as shown in Fig. S3. Since these individual interactions are ruled by the same physical effect, we combined them to define the Phe/Tyr/Trp-Phe/Tyr/Trp group potential; for this purpose, we shifted the inter-residue distances *d* of the larger residues towards smaller distances by subtracting the difference in radii between the larger amino acid and the smallest residue in the group; the minimum number of occurrences per bin was here chosen to be 20 instead of 10 (see Eq. (6)).

**Figure 2:**
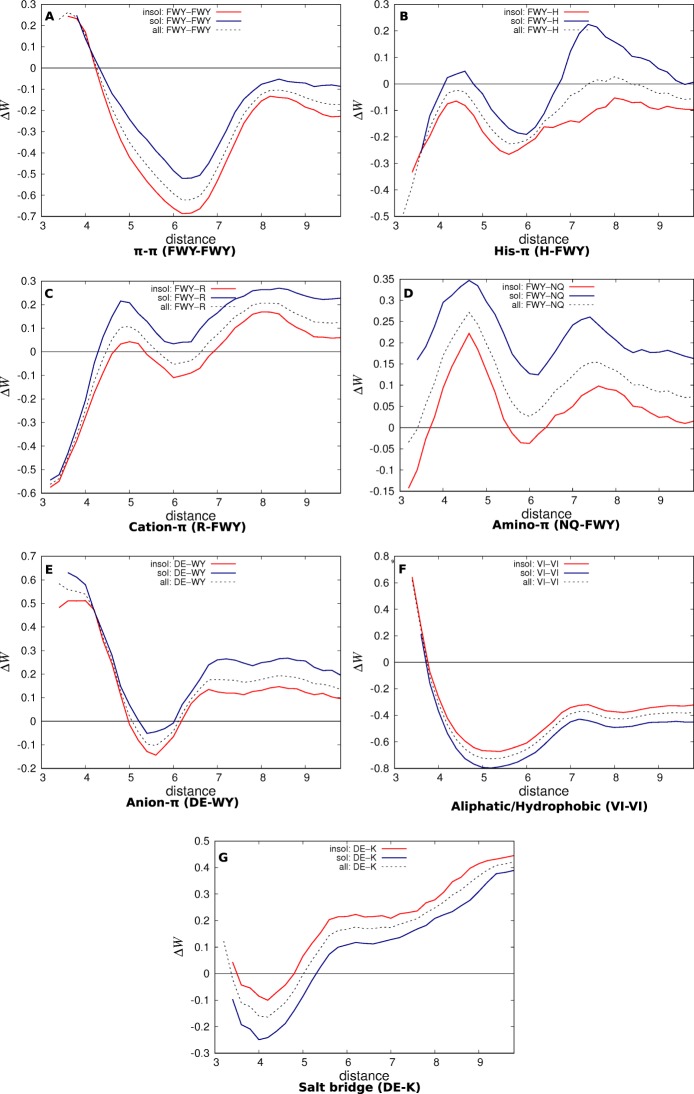
Residue-residue group potentials derived from datasets of soluble, aggregation-prone and all proteins (${{\mathscr{D}}}^{{\rm{sol}}}$, ${{\mathscr{D}}}^{{\rm{insol}}}$ and ${{\mathscr{D}}}^{{\rm{tot}}}$). The energies are in kcal/mol, the distance *d* is computed between the residue side chain centroids of the smallest amino acids in the group, and the residue pairs are separated by at least 8 residues along the chain. Distance bins containing twenty occurrences or less are not drawn.

The aromatic-aromatic group potential is shown in Fig. 2A. The large separation between the two profiles, with the profile obtained from the soluble potential above the profile obtained from the insoluble potential for all distance bins, indicates that these interactions tend to reduce the solubility of the proteins, even though they remain important for promoting their structural stability. The minimum of both profiles is located at about 6.3 Å, which corresponds to the usual distance between the side chain centers of two interacting phenylalanines, the smallest aromatic amino acids in this group. The separation of the curves in this distance range is quite high, *i*.*e*. around 0.2 kcal/mol, which shows the significantly larger importance of this interaction in aggregation-prone proteins.

#### His-aromatic or His-*π* interactions

The aromatic amino acid histidine is quite special as its imidazole ring can be positively charged or neutral depending on the environmental conditions; its pKa is indeed equal to 6.8. Hence, when the histidine is neutral, its aromaticity allows it to form *π*–*π* interactions with itself and with the other aromatic residues Phe, Tyr, Trp, as well as cation-*π* interactions with the positively charged residues Lys and Arg. When the histidine is positively charged, it can play the role of cation in cation-*π* interactions with aromatic residues Phe, Tyr and Trp. These His-containing interactions are known to substantially contribute to protein stability^39^.

As expected from the similarity with the aromatic-aromatic interactions described in the previous subsection and the cation-*π* interactions presented in the next, His-aromatic interactions promote protein aggregation rather than solubility, as shown by the individual pair potentials (Fig. S3) and the group potential His-Phe/Tyr/Trp (Fig. 2B), obtained from the individual pair potentials in the same way as the *π*-*π* group potential.

#### Cation-*π* interactions

Cation-*π* interactions in proteins link the aromatic moiety of a Phe, Tyr, or Trp side chain with the cationic moiety of a Lys or Arg side chain, positioned above (or below) the aromatic ring where there is an excess of (delocalized) electrons. This interaction plays an important role in protein stabilization and contributes favorably to protein-protein binding and recognition^40,41^.

Here we make a distinction between the cation-*π* interactions involving lysines and arginines, since they differ in their solubility dependence. As shown in Tables 1 and S2 and Fig. S3, the Arg-*π* interactions are significantly more favorable in aggregation-prone than in soluble proteins, unlike Lys-*π* interactions; only Lys-Trp satisfies the statistical significance criteria.

The strong insolubilizing nature of Arg-*π* interactions is clearly shown in the group potential Arg-Phe/Tyr/Trp (Fig. 2C). The difference between the profiles obtained from the soluble and aggregation-prone protein datasets is about 0.2 kcal/mol, and thus highly significant.

The difference in behavior between Arg-*π* and Lys-*π* cation-*π* interactions is rooted in the intrinsic differences between the two positively charged amino acids: the positive charge in Lys is localized on the ammonium group, while the Arg charge is delocalized on the guanidinium group with three resonating forms. Thus in addition to the electrostatic contribution that is similar for Arg-*π* and Lys-*π* interactions, Arg-*π* is stabilized through the overlap of the molecular *π*-orbitals of Arg and the aromatic side chain, and thus by the London dispersion force^42^. As in the case of the *π*-*π* and *π*-His interactions, this type of force reduces the solubility and promotes aggregation.

#### Amino-aromatic or amino-*π* interactions

Amino-*π* interactions connect the aromatic side chain of Phe, Tyr or Trp with the side chain amide group of asparagine or glutamine^43^. The geometry of this interaction is quite similar to that of cation-*π* interactions, where the partial positive charge *δ*_+_ on the amino group of Asn or Gln (in one of the resonating forms) interacts with the *δ*_−_ located above or below the aromatic ring. However, in contrast to cation-*π* interactions, the electrostatic contribution is unfavorable in Asn/Gln-*π*. Instead, this interaction is exclusively stabilized by London dispersion forces, which involve electron correlation contributions. Note that the strength of the latter forces in Asn/Gln-*π* interactions are similar to that in Arg-*π*^42^.

The group potential Asn/Gln-Phe/Tyr/Trp is depicted in Fig. 2D. Amino-*π* interactions are found to be favorable in aggregation-prone proteins, and unfavorable in soluble ones. The distance between the soluble and insoluble energy profiles is here also about 0.2 kcal/mol.

#### Anion-aromatic or anion-*π* interactions

Anion-*π* interactions are established between a residue with an aromatic moiety and a residue with an anionic side chain, *i*.*e*. between Phe, Tyr or Trp and Asp or Glu. They are stabilized through anion-quadrupole interactions between the *δ*_+_ edge of the aromatic ring and the anion, as well as through the overlap of *π*-orbitals and thus London interactions. In our analysis, the anion-*π* interactions, like the other interactions involving delocalized *π*-electrons, promote insolubility and aggregation^44,45^.

Note however that we did not include Phe in the anion-*π* group potential showed in Fig. 2E, as the anion-Phe interactions behave differently from anion-Tyr and anion-Trp. Indeed, anion-Phe interactions are unfavorable in all distance ranges, as we can see in Fig. S3. Moreover, the difference between the profiles derived from soluble and aggregation-prone proteins seems more associated to a distance shift in Asp-Phe, with the residues more closely packed in the soluble proteins. This difference could be due to the marked hydrophobicity of Phe or to the absence of extracyclic atoms whose presence in Tyr and Trp anion-*π* could provide stabilization effects. Note also that Asp, but not Glu, satisfies the statistical significance criteria (Tables 1 and S2). The Glu-Tyr/Trp interactions show the same trend as Asp-Tyr/Trp but to a lesser extent.

#### Other interactions

The large majority of the other interactions that promote insolubility have at least one of the interacting residues that contain *π*-delocalized electrons. Among these, we find sulfur-aromatic interactions between a cysteine and an aromatic residue, especially Phe and Trp. Note that sulfur-aromatic interactions involving a methionine and Phe or Trp also promote insolubility, as seen in Fig. S3, but do not satisfy the statistical significance criteria. In these interactions, the partial negative charge *δ*_−_ on the sulfur group of the side chain of the Cys or Met side chain interacts with the *δ*_+_ on the edge of the aromatic ring^46^.

In this group we also find Arg-Arg interactions, which are obviously unfavorable because of the proximity of the two positive charges, but are significantly less unfavorable in insoluble than in soluble proteins. Again, this can be explained by the London dispersion force contributions due to the overlap of the *π*-orbitals of the arginines, which is less unfavorable in aggregation-prone proteins.

Similarly, the Asn-Gln interactions - and also the Asn-Asn and Gln-Gln even though they do not satisfy the statistical significance criteria -, which involve London dispersion forces, have more favorable energy profiles when computed from aggregation-prone proteins.

#### Relative orientation of the interacting *π*-planes

In view of deepening the understanding of the relation between *π*-*π*, His-*π*, Arg-*π* and amino-*π* interactions and solubility, we analyzed the geometry of their conformations in the soluble and insoluble protein datasets ${{\mathscr{D}}}^{{\rm{sol}}}$ and ${{\mathscr{D}}}^{{\rm{insol}}}$. For that purpose, we used an in-house program^47^ that detects these interactions and characterizes their geometry; in particular, it computes the angle between the *π*-planes. We found a significantly higher number of such interactions in insoluble than in soluble proteins - in agreement with their more favorable energy profiles -, but no significant difference between their conformational geometries. Thus, for aromatic-aromatic interactions, there does not seem to be a statistically significant preference for T-shaped, face-to-face or off-stacked geometries. There is also no preferred geometry for His-$\pi$, Arg-$\pi$ and amino-$\pi$ interactions.

### Interactions that increase solubility

The residue pairs for which the potential derived from soluble proteins is significantly more favorable than the potential derived from aggregation-prone proteins are listed in Tables 2 and S3 and shown in Fig. S3. There are 22 residue-residue interactions of this type with the statistical significance criterion ${{\rm{Sig}}}_{ {\mathcal M} }\ge 0.95$ and ${{\rm{Sig}}}_{{\mathscr{V}}}\ge 0.95$, and 27 if the less strict criterion Sig${{\rm{Sig}}}_{ {\mathcal M} }\ge 0.95$ or ${{\rm{Sig}}}_{{\mathscr{V}}}\ge 0.95$ is used.

Two main conclusions can be drawn from these tables. The first is that aliphatic residues have the driving role for promoting solubility. Indeed, most interactions involve at least one aliphatic residue. The second conclusion is that lysine-involving salt bridges also favor solubility.

#### Aliphatic-aliphatic interactions

The four residues alanine, valine, isoleucine and leucine have only C heavy atoms on their side chain and are thus aliphatic. Their hydrophobicity increases with increasing number of C atoms. Ala can thus be found both in the protein core and at the surface, while the Val, Leu and Ile are predominantly in the core. Glycine, which has only an H atom as side chain, is often added to the aliphatic amino acid group.

The subset of aliphatic amino acids which are also hydrophobic (Val, Ile, Leu) are well known to play a fundamental role in the stabilization of the folded protein structure by contributing to the formation of the hydrophobic core^48^. Indeed, though these residues do not form physical interactions, they cluster together to avoid any contact with the solvent.

Our results show that the effective interactions between aliphatic residues are more favorable as their hydrophobicity increases, and appear stabler in soluble than in aggregation-prone proteins (Fig. S3; Tables 2 and S3). This suggests that the core is more hydrophobic and stable in soluble proteins. This characteristic is likely to help during the folding process to avoid some unwanted interactions between partially folded structures that could lead to aggregation phenomena.

There is, however, a counterexample to this rule: the aliphatic interactions involving leucine have a different behavior than those involving other aliphatic residues. Despite their similar chemical properties, the Leu-Leu interaction does not show any difference whether computed from the soluble or insoluble protein datasets (Fig. S3). This result could be put in relation with the different secondary structure propensities of Leu compared to Ile and Val, and also with its different thermal propensities^34^, but a deeper investigation is needed to explain this counterintuitive behavior. Therefore, we showed in Fig. 2F the group potential involving only Val and Ile residues.

At first sight, the understanding of the role of the hydrophobicity in promoting solubility seems unclear. Indeed, interactions between hydrophobic aliphatic residues (except Leu) are more frequent in soluble proteins whereas interactions between aromatic residues, which are also hydrophobic, are more frequent in aggregation-prone proteins. Different analyses reported in the literature actually reach contradictory conclusions on the role of hydrophobicity: indications that the average protein hydrophobicity is anti-correlated with its solubility is presented in an early study^49^, while more recent investigations point out that only exposed hydrophobic patches seems to be related to insolubility^49,50^. The key result of the present paper that allows reconciling these views is that it is not the hydrophobicity that matters for solubility, but rather the absence or presence of interactions involving delocalized *π*-electrons.

Note finally that, in an extensive amino acid sequence-based analysis^16^, no significant difference was observed between the relative content of aliphatic hydrophobic residues (Val, Ile, Leu) in soluble and insoluble proteins. However, the difference in protein length between the sets of soluble and insoluble proteins was overlooked. Indeed, soluble proteins are smaller than insoluble proteins (214 residues versus 287 on the average in the ${{\mathscr{D}}}^{{\rm{sol}}}$ and ${{\mathscr{D}}}^{{\rm{insol}}}$ sets). The percentage of Val, Ile and Leu residues is only marginally different in the two sets: 23.4% in ${{\mathscr{D}}}^{{\rm{sol}}}$ and 22.5% in ${{\mathscr{D}}}^{{\rm{insol}}}$, with a low statistical significance (Kolmogorov-Smirnov P-value = 0.03). However, the percentage of these residues that are in the protein core is about 40.1% and 36.7% in ${{\mathscr{D}}}^{{\rm{sol}}}$ and ${{\mathscr{D}}}^{{\rm{insol}}}$, respectively (Kolmogorov-Smirnov P-value < 10^−5^). This shows that the number of Val, Ile and Leu residues is about the same, but that the frequency of these residues is higher in the core of soluble proteins than in the core of aggregation-prone proteins.

#### Lys-containing salt bridges

A salt bridge is a short-range electrostatic interaction formed by two residues of opposite charge. An example of this interaction is shown in Fig. 1 for the Lys-Asp pair computed from the two datasets ${{\mathscr{D}}}^{{\rm{sol}}}$ and ${{\mathscr{D}}}^{{\rm{insol}}}$. The three other salt bridge pairs are Lys-Glu, Arg-Glu, Arg-Asp. The potentials for these four interactions have all a minimum located at an inter-residue distance of about 4 Å, which is the common distance associated to salt bridge formation.

We found that salt bridges involving lysine (Figs 2G and S3) are significantly more favorable in soluble proteins than in weakly soluble ones. For salt bridges involving arginine, on the contrary, no significant difference is observed between the energy profiles derived from both types of proteins.

These results, as well as those of the previous section showing that arginine favors aggregation propensities, are in agreement with the observed tendencies of the lysine/arginine ratio to be well correlated with an increased solubility^18^. They are also in agreement with the finding that large patches with a net positive charge disfavor protein solubility especially when there is an Arg prevalence in the patch^17^. The conclusion of the absence of correlation between the solubility and the positively charged residue content, found in^16^, does not contradict the results of this paper, since no difference is made between the chemical properties of Arg and Lys. Instead, they observed the statistically significant trend that Asp/Glu-rich proteins are more soluble than Asp/Glu-poor ones.

### Correlation between solubility and stability

To test how the energies computed with the newly developed solubility-dependent statistical potentials correlate with solubility, we started by computing, for each protein of the ${{\mathscr{D}}}^{{\rm{tot}}}$ set, the three folding free energy values ${\rm{\Delta }}{W}_{S,C}^{{\rm{sol}}}$, ${\rm{\Delta }}{W}_{S,C}^{{\rm{insol}}}$ and ${\rm{\Delta }}{W}_{S,C}^{{\rm{all}}}$, defined in Eq. (10). These energies and the associated experimental solubility values ${\mathscr{S}}$ are reported in Table S1.

To evaluate the energy-solubility correlation, we used leave-one-out cross validation (see Methods). The Pearson correlation coefficient between the solubility ${\mathscr{S}}$ and the folding free energy values ${\rm{\Delta }}{W}_{S,C}^{{\rm{sol}}}-{\rm{\Delta }}{W}_{S,C}^{{\rm{insol}}}$ and ${\rm{\Delta }}{W}_{S,C}^{{\rm{all}}}$ are given in Table 3. We also computed the correlation of ${\mathscr{S}}$ with different sequence features, namely the protein length, the isoelectric point and the aliphatic index (defined as the relative volume of a protein occupied by aliphatic side chains)^51^, as they have been suggested to be related to solubility^16,52,53^.

**Table 3:** Correlation between experimental solubility, folding free energies and sequence-derived features.

	Solubility $\pmb{\mathscr{S}}$	Length	Isoelectric point	Aliphatic Index
Solubility ${\mathscr{S}}$	—	−0.31	−0.18	0.11
${\rm{\Delta }}{W}_{S,C}^{{\rm{insol}}}-{\rm{\Delta }}{W}_{S,C}^{{\rm{sol}}}$	**0**.**39**	−0.33	−0.11	0.37
${\rm{\Delta }}{W}_{S,C}^{{\rm{tot}}}$	0.20	−0.65	0.12	−0.30

Interestingly, we found that the folding free energy difference (${\rm{\Delta }}{W}_{S,C}^{{\rm{insol}}}$ − ${\rm{\Delta }}{W}_{S,C}^{{\rm{sol}}}$) correlates with ${\mathscr{S}}$ with quite a high correlation coefficient (r = 0.39), and outperforms all other features tested. This means that, the more favorable the energy computed with the potentials derived from soluble proteins compared to that obtained with aggregation-prone proteins, the more soluble the protein. This constitutes a strong check of the performance and robustness of our solubility-dependent statistical potentials that are able to accurately capture the solubility properties of proteins. Note that the energy ${\rm{\Delta }}{W}_{S,C}^{{\rm{tot}}}$ is also correlated with the solubility (r = 0.20), but less than our new solubility-dependent statistical potentials. Based on these results, we are currently using the energy difference (${\rm{\Delta }}{W}_{S,C}^{{\rm{insol}}}$ − ${\rm{\Delta }}{W}_{S,C}^{{\rm{sol}}}$) as a novel feature in developing a structure-based solubility predictor.

Among the three tested sequence-based features, the protein length has the best score: it is significantly anti-correlated with ${\mathscr{S}}$, with a correlation coefficient r = −0.31. This means that smaller proteins have the tendency to be more soluble, in agreement with earlier findings^16,24^. Protein length is therefore widely used as a feature in different solubility predictors^15,19^. Not surprisingly, protein length is anticorrelated with the free energy difference $({\rm{\Delta }}{W}_{S,C}^{{\rm{insol}}}-{\rm{\Delta }}{W}_{S,C}^{{\rm{sol}}})$ (r = −0.33).

Finally, the correlation between ${\mathscr{S}}$ and the two other sequence-based quantities that are commonly considered as related to solubility is rather low. It is positive (r = 0.11) for the aliphatic index, which confirms the trends found from the analysis of aliphatic interactions (see previous subsection). The correlation is negative (r = −0.18) for the isoelectric point, as already observed earlier^16,24^. The low correlation could be attributed to the fact that no difference is made between Lys and Arg, which yet have different effects on the solubility.

### Testing other datasets and solubility definitions

The solubility ${\mathscr{S}}$ (in %) used in this paper is the concentration of the soluble protein fraction over the total concentration of the protein, measured under fixed conditions^16^, and is possibly affected by the fact that the total concentration is not the same for all proteins. It may differ from the common definition of solubility ${{\mathscr{S}}}_{0}$ (in g/l), which is the concentration of protein in a saturated solution that is in equilibrium with a solid phase. As this quantity is often difficult to measure, a common strategy consists of adding precipitants in various concentrations and extrapolating the results to zero concentration. However, the results may depend on the type of precipitant and the validity of the extrapolation is questionable^14^.

The first solubility definition (${\mathscr{S}}$) was used to derive our solubility-dependent potentials, since it is compatible with large-scale analyses and thus with large datasets^16^. We assessed the performance of these potentials on other datasets described in the literature, which use the same or other solubility definitions, by computing the linear correlation coefficient *r* between the solubility values and the energy difference (${\rm{\Delta }}{W}_{S,C}^{{\rm{insol}}}-{\rm{\Delta }}{W}_{S,C}^{{\rm{sol}}}$). They have to be compared with $r=0.39$ obtained in cross validation on the ${{\mathscr{D}}}^{{\rm{tot}}}$ set (Table 3). The results are summarized below:

- Solubility ${\mathscr{S}}$: another dataset has recently been published, with solubility data of yeast proteins rather than *E*. *coli* proteins^52^. The correlation coefficient *r* on the subset of 54 proteins for which an experimental structure is available (obtained with the same criteria as for the construction of the ${{\mathscr{D}}}^{{\rm{tot}}}$ set), is equal to 0.41.
- Solubility ${{\mathscr{S}}}_{0}$: the solubility of TEV protease, eight single mutants and a double mutant has been assayed by concentrating the proteins^54^. The *r*-value on this set is as high as 0.70.
- Solubility ${{\mathscr{S}}}_{0}$ measured using precipitants: the solubility of seven proteins has been estimated using two different precipitants, polyethylene glycol (PEG) and ammonium sulfate^14^. For six out of the seven proteins, *r* is equal to 0.40 when the solubility is extrapolated to zero PEG concentration, and to 0.07 when extrapolated at zero ammonium sulfate concentration; this indicates that the type of precipitant has an effect on the measured solubility values. The correlation is much higher (0.59 and 0.67) with the solubilities measured at non-zero precipitant concentration, for both types of precipitant, which suggests possible inaccuracies due to the extrapolation.

Thus, our solubility-dependent potentials appear to be suitable for estimating the solubilities ${\mathscr{S}}$ and ${{\mathscr{S}}}_{0}$ on different datasets, except when the measured values depend too much on some added precipitant.

## Conclusion

Even though the structural and stability properties of proteins are of fundamental importance for the biophysical understanding of solubility data, obtained for example from cell-free expression systems, their precise role is not yet clear. Sometimes, the literature even reports contradictory results. Due to the complexity of the problem, it is probably impossible to find a unique mechanism that promotes solubility or aggregation propensities. Instead, these properties are likely to be associated with an intricate combination of physical tendencies that can moreover be protein-, function- or environment-dependent.

In this paper, we tackled the solubility issue by defining new knowledge-based mean force potentials that depend on the protein solubility. They were derived from sets of proteins with known 3D structures and solubility, which were divided into subsets on the basis of their solubility values. These potentials were used to investigate the relation between the amino acid interactions and the solubility propensity. This is possible as these potentials are effective potentials and thus include the impact of the solvent on protein stability. Note that the solubility-dependent potentials that we obtained only marginally depend on the threshold values used for dividing the full protein set into soluble and aggregation-prone proteins. Indeed, as shown in Fig. S4, using stricter threshold values does not modify significantly the potentials.

The main quantitative results that we obtained pinpoint the role of charge delocalization. We indeed found that all the interactions that involve residues with delocalized *π*-electrons on their side chain disfavor solubility. This is the case of the aromatic residues Phe, Tyr and Trp, of the aromatic and sometimes positively charged residue His, of the positively charged Arg, of Gln and Asn that possess a side chain amide group, and of the negatively charged residues Asp and Glu. These residues make *π*–*π*, His-*π*, cation-*π*, amino-*π*, and anion-*π* interactions, which appear to stabilize more strongly insoluble than soluble proteins. In contrast, the interactions that promote protein solubility are salt bridges that involve Lys, aliphatic-aliphatic interactions, and some aliphatic-containing interactions. Note that none of the latter involve aromatic residues, His, Arg, Asn or Gln. Some however involve Glu or Asp, which indicates that these negatively charged residues promote aggregation only when interacting with other *π*-systems.

The biophysical explanation of these results is not totally clear. However, we can argue that interactions involving delocalized *π*-electrons are more prone to occur across protein-protein interfaces, and thus lead to aggregation phenomena. The frequent occurrence of cation-*π* and *π*-*π* interactions in protein-protein interactions has already been discussed^38,55^. In contrast, interactions between hydrophobic aliphatic residues are likely to favor the stability of the hydrophobic core in the folding process, hence avoid dangerous interactions between partially folded structures, and promote protein solubility. To check and fully understand these tendencies and interpretations, other experiments and/or quantum chemistry calculations are needed.

The present analysis is mainly focused on solubility values on the *E*. *coli* proteome, but our solubility-dependent potentials were also tested on the yeast proteome^52^ as well as on smaller datasets where the solubility is defined and experimentally measured in different ways^14,54^. The results are quite encouraging, but need to be further analyzed in view of setting up an efficient solubility predictor. Other features should possibly also be taken into account, such as the presence of intrinsically disordered sequence regions, which seem to favor aggregate formation in eukaryotes^52^.

The understanding of the solubilization and aggregation mechanisms and the role of specific residue interactions has a lot of extremely useful applications in rational protein design studies. Indeed, the solubility is often a bottleneck in academic, medical and industrial processes that require high concentrations of proteins. Although the present study is far from solving completely the solubility and aggregation issues, it is a significant step forward in this direction.

## Electronic supplementary material

Supplementary Information

## References

[1] Fink AL (1998). Protein aggregation: folding aggregates, inclusion bodies and amyloid. Fold. design.

[2] Chiti F, Dobson CM (2006). Protein misfolding, functional amyloid, and human disease. Annu. Rev. Biochem..

[3] Bucciantini M (2002). Inherent toxicity of aggregates implies a common mechanism for protein misfolding diseases. Nature.

[4] Irvine GB, El-Agnaf OM, Shankar GM, Walsh DM (2008). Protein aggregation in the brain: the molecular basis for alzheimer’s and parkinson’s diseases. Mol. medicine.

[5] Ross CA, Poirier MA (2004). Protein aggregation and neurodegenerative disease. Nat. medicine.

[6] Baneyx F, Mujacic M (2004). Recombinant protein folding and misfolding in escherichia coli. Nat. biotechnology.

[7] Singh SM, Panda AK (2005). Solubilization and refolding of bacterial inclusion body proteins. J. bioscience bioengineering.

[8] Vallejo LF, Rinas U (2004). Strategies for the recovery of active proteins through refolding of bacterial inclusion body proteins. Microb. cell factories.

[9] Rudolph R, Lilie H (1996). In vitro folding of inclusion body proteins. The FASEB J.

[10] Pédelacq J-D (2002). Engineering soluble proteins for structural genomics. Nat. biotechnology.

[11] Schmid MB (2002). Structural proteomics: the potential of high-throughput structure determination. Trends microbiology.

[12] Wilkinson DL, Harrison RG (1991). Predicting the solubility of recombinant proteins in escherichia coli. Nat. Biotechnol..

[13] Trevino SR, Scholtz JM, Pace CN (2008). Measuring and increasing protein solubility. J. pharmaceutical sciences.

[14] Kramer RM, Shende VR, Motl N, Pace CN, Scholtz JM (2012). Toward a molecular understanding of protein solubility: increased negative surface charge correlates with increased solubility. Biophys. journal.

[15] Smialowski P, Doose G, Torkler P, Kaufmann S, Frishman D (2012). Proso ii–a new method for protein solubility prediction. The FEBS journal.

[16] Niwa T (2009). Bimodal protein solubility distribution revealed by an aggregation analysis of the entire ensemble of escherichia coli proteins. Proc. Natl. Acad. Sci..

[17] Chan P, Curtis RA, Warwicker J (2013). Soluble expression of proteins correlates with a lack of positively-charged surface. Sci. Reports.

[18] Warwicker J, Charonis S, Curtis RA (2013). Lysine and arginine content of proteins: computational analysis suggests a new tool for solubility design. Mol. pharmaceutics.

[19] Hebditch M, Carballo-Amador MA, Charonis S, Curtis R, Warwicker J (2017). Protein–sol: a web tool for predicting protein solubility from sequence. Bioinformatics.

[20] Idicula-Thomas S, Kulkarni AJ, Kulkarni BD, Jayaraman VK, Balaji PV (2005). A support vector machine-based method for predicting the propensity of a protein to be soluble or to form inclusion body on overexpression in escherichia coli. Bioinformatics.

[21] Magnan CN, Randall A, Baldi P (2009). Solpro: accurate sequence-based prediction of protein solubility. Bioinformatics.

[22] Agostini F, Cirillo D, Livi CM, Delli Ponti R, Tartaglia G (2014). G. cc sol omics: a webserver for solubility prediction of endogenous and heterologous expression in escherichia coli. Bioinformatics.

[23] Sormanni P, Aprile FA, Vendruscolo M (2015). The camsol method of rational design of protein mutants with enhanced solubility. J. molecular biology.

[24] Ganesan A (2016). Structural hot spots for the solubility of globular proteins. Nat. communications.

[25] Shimizu Y, Kanamori T, Ueda T (2005). Protein synthesis by pure translation systems. Methods.

[26] Berman HM (2000). The Protein Data Bank. Nucleic Acids Res.

[27] Zhou J, Rudd KE (2013). EcoGene 3.0. Nucleic Acids Res..

[28] Altschul S, Gish W, Miller W, Myers E, Lipman D (1990). Basic local alignment search tool. J. Mol. Biol..

[29] Wang G, Dunbrack RL (2003). Pisces: a protein sequence culling server. Bioinformatics.

[30] Miyazawa S, Jernigan RL (1985). Estimation of effective interresidue contact energies from protein crystal structures: quasi-chemical approximation. Macromolecules.

[31] Sippl MJ (1990). Calculation of conformational ensembles from potentials of mean force: an approach to the knowledge-based prediction of local structures in globular proteins. J. molecular biology.

[32] Rooman MJ, Kocher J-PA, Wodak SJ (1991). Prediction of protein backbone conformation based on seven structure assignments: influence of local interactions. J. molecular biology.

[33] Kocher J-PA, Rooman MJ, Wodak SJ (1994). Factors influencing the ability of knowledge-based potentials to identify native sequence-structure matches. J. molecular biology.

[34] Folch B, Dehouck Y, Rooman M (2010). Thermo-and mesostabilizing protein interactions identified by temperaturedependent statistical potentials. Biophys. journal.

[35] Pucci F, Rooman M (2014). Stability curve prediction of homologous proteins using temperature-dependent statistical potentials. PLoS computational biology.

[36] Pucci F, Dhanani M, Dehouck Y, Rooman M (2014). Protein thermostability prediction within homologous families using temperature-dependent statistical potentials. PLoS One.

[37] Kyte, J. *Structure in protein chemistry* (Garland Science, 2006).

[38] Burley S, Petsko GA (1985). Aromatic-aromatic interaction: a mechanism of protein structure stabilization. Science.

[39] Cauët E, Rooman M, Wintjens R, Liévin J, Biot C (2005). Histidine- aromatic interactions in proteins and protein- ligand complexes: quantum chemical study of x-ray and model structures. J. chemical theory computation.

[40] Dougherty DA (2007). Cation-*π* interactions involving aromatic amino acids. The J. nutrition.

[41] Gallivan JP, Dougherty DA (1999). Cation-*π* interactions in structural biology. Proc. Natl. Acad. Sci..

[42] Biot C, Buisine E, Kwasigroch J-M, Wintjens R, Rooman M (2002). Probing the energetic and structural role of amino acid/nucleobase cation-*π* interactions in protein-ligand complexes. J. Biol. Chem..

[43] Burley S, Petsko G (1986). Amino-aromatic interactions in proteins. FEBS letters.

[44] Schottel BL, Chifotides HT, Dunbar KR (2008). Anion-*π* interactions. Chem. Soc. Rev..

[45] Philip V (2011). A survey of aspartate- phenylalanine and glutamate- phenylalanine interactions in the protein data bank: searching for anion-*π* pairs. Biochemistry.

[46] Hunter CA, Singh J, Thornton JM (1991). *π*-*π* interactions: the geometry and energetics of phenylalanine-phenylalanine interactions in proteins. J. molecular biology.

[47] Wintjens R, Liévin J, Rooman M, Buisine E (2000). Contribution of cation-*π* interactions to the stability of protein-dna complexes1. J. molecular biology.

[48] Pace CN (2011). Contribution of hydrophobic interactions to protein stability. J. molecular biology.

[49] Mosavi LK, Peng Z-Y (2003). Structure-based substitutions for increased solubility of a designed protein. Protein engineering.

[50] Damodaran, S. & Parkin, K. L. *Fennema’s food chemistry* (CRC press, 2017).

[51] Gasteiger, E. *et al*. Protein identification and analysis tools on the expasy server. In *The proteomics protocols handbook*, 571–607 (Springer, 2005).

[52] Uemura E (2018). Large-scale aggregation analysis of eukaryotic proteins reveals an involvement of intrinsically disordered regions in protein folding. Sci. reports.

[53] Idicula-Thomas S, Balaji PV (2005). Understanding the relationship between the primary structure of proteins and its propensity to be soluble on overexpression in escherichia coli. Protein Sci..

[54] Cabrita L, Gilis D, Dehouck Y, Rooman M, Bottomley S (2007). Enhancing the stability and solubility of tev protease using in silico design. Protein Sci..

[55] Crowley PB, Golovin A (2005). Cation–*π* interactions in protein–protein interfaces. Proteins: Struct. Funct. Bioinforma..

