# Supplementary material for: Computational analysis of the amino acid interactions that promote or decrease protein solubility
Source: Sci Rep. 2018 Oct 2;8:14661. doi: 10.1038/s41598-018-32988-w (PMC6168528; doi:10.1038/s41598-018-32988-w)

Supplementary Information

Computational analysis of the amino acid interactions that promote or decrease protein solubility

Qingzhen Hou, Raphaël Bourgeas, Fabrizio Pucci*, Marianne Rooman*

Department of BioModeling BioInformatics & BioProcesses,
Université Libre de Bruxelles, Brussels, Belgium

Emails: qinghou@ulb.ac.be, fapucci@ulb.ac.be, mrooman@ulb.ac.be
(*) Co-last authors

Table S1. Dataset. List of proteins used in the derivation of the (solubility)-dependent statistical potentials and their characteristics.

Table S2. Insolubilizing interactions. List of insolubilizing amino acid interactions which satisfy the relaxed significance criteria.

Table S3. Solubilizing interactions. List of solubilizing amino acid interactions which are statistically significant.

Figure S1. Solubility distribution. Distribution of solubility values $\mathcal{S}$ (in %) of the proteins from the $\mathcal{D}^{\rm tot}$ dataset.

Figure S2. Amino acid frequencies in soluble and aggregation-prone proteins. Ratio of the frequency of each amino acid in the set $\mathcal{D}^{\rm insol}$ over the frequency in the set $\mathcal{D}^{\rm sol}$, minus 1.

Figure S3. Distance potentials. Distance potentials of all 210 residue-residue pairs derived from soluble or aggregation-prone protein sets.

Figure S4. Distance group potentials. Residue-residue group potentials derived from datasets of highly soluble proteins, soluble proteins, aggregation-prone proteins, and highly aggregation-prone proteins.

Table S1. List of proteins from the $\mathcal{D}^{\text{sol}}$ set used for the derivation of solubility-dependent statistical potentials. The PDB codes of the proteins from the $\mathcal{D}^{\text{sol}}$ subset are in blue and those from $\mathcal{D}^{\text{insol}}$ are in red. The values of $\Delta W^{\text{sol}}_{S,C} - \Delta W^{\text{insol}}_{S,C}$ and $\Delta W^{\text{all}}_{S,C}$ are in kcal/mol. pI means isoelectric point and AI aliphatic index.

PDB Code	Chain	Bio Unit	Resolution (Å)	pI	AI	Length	Solubility (%)	ΔW ^{total} (kcal/mol)	ΔW ^{insol} – ΔW ^{sol} (kcal/mol)
1dnp	A	Monomer	2.3	6.7	80.4	471	2	-108.98	-88.23
3qs3	A	Monomer	2.1	4.9	72	191	2	-35.53	-12.13
2bll	A	Homo2	2.3	5.8	92.9	345	3	-92.61	-15.70
1db3	A	Homo2	2.3	5.8	84.1	372	4	-60.89	-0.56
2jlc	A	Homo2	2.5	6.2	94.3	577	4	-260.81	-44.95
2wiu	A	Hetero4	2.35	8.3	93.6	446	4	-74.41	-5.80
5kvr	A	Homo2	1.36	9.7	98.8	77	4	-19.54	-0.58
2i06	A	Monomer	2.2	9.6	100.1	309	5	-36.97	-17.61
3n75	A	Homo10	2	5.9	83.5	715	5	-205.94	-27.63
2a9w	A	Homo2	1.65	5.6	84.2	264	6	-77.98	-47.29
3qxf	A	Monomer	1.85	7.9	64.9	355	6	-46.54	-49.83
2c4n	A	Homo4	1.8	5.2	92.5	250	7	-148.72	21.83
5caj	A	Monomer	1.65	7.9	83.9	278	7	-32.44	-4.88
2cxa	A	Monomer	1.6	7.7	78.1	256	8	-76.47	-49.94
4i8o	A	Monomer	2.1	6.4	98	358	8	-104.28	-3.58
4muo	A	Homo2	1.94	6.8	89	328	8	-145.23	2.54
4xb6	D	Hetero8	1.7	5.1	74.2	281	8	-35.95	-2.90
1r8g	A	Homo2	2.15	5.7	83.7	372	9	-122.54	-42.09
1u08	A	Homo2	2.35	5.8	92.6	386	9	-177.25	-23.87
3i8s	A	Monomer	1.8	5.3	109.3	274	9	-117.93	2.28
3uqy	L	Hetero2	1.47	5.8	84.5	582	9	-167.50	-88.76
4lrz	E	Hetero4	2.32	5.5	99.2	318	9	-141.16	-24.33
1udc	A	Homo2	1.65	5.9	84.5	338	10	-124.12	-26.09
4bfa	A	Monomer	1.65	6.7	96.3	338	10	-124.38	-4.54
4lw2	A	Homo2	1.8	5.4	92.8	404	10	-190.40	-17.72
1i2k	A	Homo2	1.79	6.1	95.7	269	11	-88.85	-44.31
1lqa	A	Monomer	1.6	6.3	88.3	346	11	-110.38	-22.29
1qsa	A	Monomer	1.65	8.5	73.4	618	11	-147.27	-108.58
1v9y	A	Homo2	1.32	6.5	77.7	167	11	-9.76	3.61
1z9t	A	Monomer	1.54	6.6	76.6	255	11	-75.00	-35.32
4bh5	A	Monomer	1.57	9.7	83	142	11	-42.05	-2.29
4jrp	A	Monomer	1.95	5.5	83.3	478	11	-114.94	-47.51
1bl0	A	Monomer	2.3	9.4	64.3	129	12	-1.48	-2.97
1sur	A	Homo2	2	5.5	102.1	215	12	-52.08	-9.30
1j3e	A	Monomer	2.5	7.3	82.4	115	13	-57.83	-2.67
1j54	A	Monomer	1.7	5.3	91.8	186	13	-48.19	15.43
1zzm	A	Monomer	1.8	6.1	94.6	259	13	-118.94	8.41
3abq	A	Hetero12	2.05	4.8	91.1	453	13	-88.54	12.55
4blu	A	Monomer	1.85	8.9	93.5	289	13	-72.84	-15.21
1gvf	A	Homo4	1.45	5.4	93.8	286	14	-149.66	19.28
1uug	A	Hetero2	2.4	6.7	86.9	229	14	-83.21	-22.57
1ec7	A	Homo4	1.9	5.7	81.8	446	15	-153.06	-6.23
1k77	A	Monomer	1.63	5.1	86	260	15	-96.76	-30.61
2dgk	A	Homo6	1.9	5.3	79.5	452	15	-112.39	-53.29
2f1f	A	Homo2	1.75	8	111.7	164	15	-42.74	19.03
3asu	A	Homo4	1.9	5.7	90	248	15	-99.96	8.91
3lbf	A	Monomer	1.8	6.5	104.5	210	15	-67.89	0.26
3vus	A	Monomer	1.65	5.8	81.5	268	15	-99.01	-19.95
5dcf	A	Monomer	2.3	6.7	93.2	275	15	-83.58	-14.11
5g5g	B	Hetero3	1.7	9	96.8	318	15	-130.89	4.68
1ecf	A	Homo4	2	5.3	92	504	16	-165.18	30.39
1ej0	A	Monomer	1.5	8.6	92.5	180	16	-72.93	5.96
2iea	A	Homo2	1.85	5.5	80.7	886	16	-199.27	-14.33
2p0b	A	Monomer	1.74	6.6	54.4	163	16	-10.95	-21.51

PDB Code	Chain	Bio Unit	Resolution (Å)	pI	AI	Length	Solubility (%)	ΔW ^{total} (kcal/mol)	ΔW ^{insol} – ΔW ^{sol} (kcal/mol)
2y8p	A	Monomer	2	8	82.1	194	16	-71.93	-13.82
2zpm	A	Monomer	0.98	6.4	108.1	91	16	-31.60	6.67
3d1g	A	Homo2	1.64	5.3	94	366	16	-98.98	2.97
3lbm	A	Homo4	1.48	6.7	93.5	319	16	-147.42	-0.10
4dzd	A	Monomer	2	9.7	94.8	218	16	-62.93	-7.53
1ns5	A	Homo2	1.68	8.7	92.6	155	17	-71.98	-4.24
3abq	B	Hetero12	2.05	6.5	90.2	306	17	-149.84	16.77
3tlq	A	Monomer	1.91	5.6	106.8	242	17	-120.90	-19.39
4isa	A	Monomer	1.8	5.5	91.4	300	17	-80.69	12.38
1d2f	A	Homo2	2.5	5.3	88.4	390	18	-172.24	-35.22
2rgk	A	Homo6	2.5	5.9	76.5	421	18	-144.93	-66.03
3i3q	A	Monomer	1.4	8.9	79.2	211	18	-70.21	-30.86
4mn4	A	Hetero4	2.3	8.6	86.8	259	18	-56.34	21.10
1brm	A	Homo2	2.5	5.4	95.1	367	19	-173.67	2.73
1cyw	A	Homo2	2.5	5.3	60.1	205	19	-39.93	-5.58
1k92	A	Homo4	1.6	5.5	81.7	455	19	-103.06	-20.52
1sdi	A	Monomer	1.65	9.3	112.7	213	19	-150.56	-11.03
3d2y	A	Monomer	1.75	6.4	83.5	261	19	-45.40	-35.64
1id0	A	Monomer	1.6	4.8	95.5	152	20	-54.50	7.14
1l2p	A	Monomer	1.55	9.2	80.3	61	20	-23.56	7.64
2icu	A	Monomer	1.6	5.2	73.8	229	20	-69.70	-23.99
2wsk	A	Monomer	2.25	5.7	83.7	657	20	-147.99	-140.37
3b8o	A	Homo8	2.4	5.2	76.7	265	20	-4.08	-25.00
3gfh	A	Homo3	2.2	5.5	91.7	225	20	-120.33	13.76
4dap	A	Monomer	2.2	6.5	81.4	234	20	-49.73	-10.58
4lov	A	Monomer	1.5	5.6	88.7	158	20	-70.15	-6.12
2o99	A	Homo2	1.7	6.4	93.4	182	21	-59.72	1.07
4cvq	A	Homo2	2.1	5.9	93.3	431	21	-136.14	-1.60
4zvf	A	Monomer	1.15	8	93.6	172	21	-72.62	2.70
2qia	A	Homo3	1.74	6.6	93.8	262	22	-155.59	33.64
4bht	A	Homo6	2.5	6	79.9	447	22	-182.56	17.32
4bjq	A	Homo2	2.1	9.5	102.7	78	22	-28.32	0.75
1mug	A	Monomer	1.8	9.2	91.1	168	23	-66.36	1.97
1owg	B	Homo2	2.1	9.3	69.6	94	23	-14.04	5.72
1tke	A	Monomer	1.46	5.7	83.2	224	23	-52.82	-4.90
2r19	A	Homo4	2.16	7.1	72.8	159	23	3.66	7.81
3dhx	A	Homo2	2.1	5.4	84.6	106	23	-35.91	4.35
3foz	A	Homo2	2.5	5.7	96.5	316	23	-125.86	-12.54
4xpu	A	Monomer	2.4	7.9	93.6	217	23	-78.91	-7.60
1aj2	A	Monomer	2	5.7	97.6	282	24	-139.13	13.25
1ako	A	Monomer	1.7	5.8	77.5	268	24	-39.08	-2.40
1txk	A	Monomer	2.5	6.4	69.7	498	24	13.94	-40.23
3brq	A	Homo2	2	6.1	99.3	296	24	-108.80	-10.97
3ca8	A	Monomer	1.8	5.5	92.1	266	24	-124.57	-35.10
2rdz	A	Homo2	1.74	6.3	65.8	452	25	-19.60	-37.74
1eq2	A	Homo5	2	4.8	78.4	310	26	-73.26	19.69
1zp3	A	Homo2	1.85	6.2	84.1	304	26	-118.19	-20.42
2hds	A	Monomer	1.16	8.8	85.9	358	26	-105.76	-28.32
4h8a	A	Homo2	1.64	5.8	86.1	339	26	-137.25	-9.62
4iff	A	Homo2	2.3	4.4	81.7	86	26	-11.62	-1.68
4kie	A	Monomer	1.7	5.9	99.3	279	26	-115.49	8.64
1a3a	A	Homo2	1.8	4.9	100.8	148	27	-83.16	25.51
4xb6	A	Hetero6	1.7	6.5	82.2	150	27	-44.07	-15.97
1b12	A	Monomer	1.95	5.5	74.6	248	28	7.62	0.55
1cl1	A	Homo4	1.83	6	95.4	395	28	-193.71	-21.02

PDB Code	Chain	Bio Unit	Resolution (Å)	pI	AI	Length	Solubility (%)	ΔW ^{total} (kcal/mol)	ΔW ^{insol} – ΔW ^{sol} (kcal/mol)
2dbi	A	Monomer	2.05	5.7	76.5	461	28	-105.22	-53.22
1nnx	A	Monomer	1.45	4.7	74	109	29	-6.56	14.52
1d9e	A	Homo4	2.4	6.3	91.3	284	30	-89.91	25.01
1qtq	A	Monomer	2.25	5.9	78.3	553	30	-50.81	-69.77
2iw1	A	Monomer	1.5	7.2	82.5	374	30	-136.87	-14.86
3zdb	A	Homo2	1.47	6.2	90.2	251	30	-95.98	-27.80
1iye	A	Homo6	1.82	5.5	84.3	309	31	-90.20	-17.47
1ks9	A	Monomer	1.7	5.5	104.6	291	31	-140.65	-13.11
3htv	A	Homo2	1.95	6.6	87.6	310	31	-114.58	-15.42
1hzt	A	Monomer	1.7	6.2	79.1	190	32	-21.06	-24.89
1kg2	A	Monomer	1.2	9.4	82.8	225	32	-113.86	-30.56
2hqs	A	Hetero2	1.5	6.8	74.5	415	32	-12.62	-30.03
2pqx	A	Monomer	1.42	8.6	64.3	245	32	-60.69	-19.72
2qdf	A	Monomer	2.2	5.7	89.8	335	32	-63.89	26.37
2x5j	O	Homo4	2.3	6.3	98.1	339	32	-142.23	-5.74
3edc	A	Homo4	2.1	6.4	103.5	360	32	-153.09	-9.77
3n37	A	Homo2	1.65	4.7	87.2	319	32	-65.04	-19.37
3sxu	B	Hetereo2	1.85	5.5	98.3	138	32	-54.67	-13.90
4bin	A	Monomer	2.49	9.4	86.4	403	33	-60.64	17.98
1bf6	A	Monomer	1.7	5.3	86.8	291	34	-85.61	10.12
2y4d	A	Homo2	2	6.1	82.6	403	34	-44.25	-32.20
3glc	A	Homo10	2.5	6.1	87	295	34	-129.28	21.62
3l9w	A	Homo2	1.75	6.1	84.4	413	34	-119.11	-25.66
3by8	A	Monomer	1.45	8.3	96.9	142	35	-63.60	5.49
1nxu	A	Homo2	1.8	5.2	87.4	333	37	-83.15	-20.96
1vh4	A	Homo2	1.75	6.4	89.8	435	37	-52.52	-43.28
2bjw	A	Monomer	1.75	5.1	96.8	265	37	-85.69	-13.35
2dg5	B	Hetero2	1.6	5.1	85.1	190	37	-36.05	13.11
3a7r	A	Monomer	2.05	5.7	82.3	337	37	-78.15	-52.38
3fwz	A	Homo2	1.79	4.9	115	140	37	-92.82	18.47
4hl6	A	Homo2	2.12	6.1	84.4	401	37	-117.68	-22.76
1bia	A	Monomer	2.3	7.8	110	321	38	-138.48	1.28
1j1v	A	Monomer	2.1	9.3	90.3	94	38	-26.70	-7.70
1jke	A	Homo2	1.55	4.8	86.7	145	38	-60.78	11.16
1ydy	A	Homo2	1.7	5.4	75.6	356	39	-15.01	7.27
3nre	A	Monomer	1.59	4.8	68.4	291	39	-35.80	-61.69
4auk	A	Monomer	1.9	7.6	79.8	375	39	-120.90	-40.77
4d02	A	Homo2	1.76	5	80	479	39	-105.20	-10.72
1sq5	A	Homo2	2.2	6.3	99.7	308	40	-93.19	-8.13
1fhu	A	Monomer	1.65	4.8	99.4	320	41	-85.71	-17.48
1gnk	A	Homo3	2	5.8	114	112	41	-38.25	33.52
1ivn	A	Monomer	1.9	6.3	87.9	190	41	-62.35	-20.17
3ipo	A	Homo2	2.4	6	75.4	416	41	-90.54	-68.45
2i82	A	Monomer	2.05	7.8	83.6	217	44	-7.43	3.74
2oug	A	Monomer	2.1	8.6	93.3	162	44	-41.50	0.41
2yh9	A	Homo6	1.8	6.7	68.6	88	44	-13.33	-6.64
3atp	A	Homo2	2.5	5	76.5	170	44	-18.19	-24.89
3hwo	A	Monomer	2.3	5.6	83.9	394	44	-125.06	-23.24
3l6i	A	Monomer	2.01	6.5	85.1	181	45	-13.56	24.62
1mxr	A	Homo2	1.42	4.7	92.8	375	46	-62.68	-30.41
3fmy	A	Homo2	1.4	9.5	90.8	73	46	-22.88	9.03
3lpf	A	Homo4	2.26	5.3	77.5	605	46	-30.32	-63.73
4ou6	A	Homo5	1.96	6.9	83.5	76	46	-19.11	-9.36
1oyw	A	Monomer	1.8	6.9	92.9	523	47	-179.83	-27.67
1rg9	A	Homo4	2.5	5.1	86.9	383	48	-59.48	24.04

PDB Code	Chain	Bio Unit	Resolution (Å)	pI	AI	Length	Solubility (%)	ΔW [total] (kcal/mol)	ΔW [insol] – ΔW [sol] (kcal/mol)
1f0k	A	Homo2	1.9	9.6	93.6	364	49	-226.69	13.46
1qsg	A	Homo4	1.75	5.7	91.4	265	49	-145.06	5.12
1u2m	A	Hetero3	2.3	9.5	76.4	143	49	-35.90	10.08
2fsu	A	Monomer	1.7	6	100.4	210	49	-75.56	-5.48
4k22	A	Homo4	2	6.6	93.1	365	49	-148.62	-33.52
2gqr	A	Homo2	2	5.1	88	237	50	26.65	11.47
3a2z	A	Monomer	1.5	4.5	80.1	197	50	-47.71	-15.56
3h9c	A	Monomer	1.4	5.6	77.4	547	50	-140.75	-42.24
1dnl	A	Homo2	1.8	9.4	82.3	199	51	-21.52	-18.13
1v58	A	Homo2	1.7	7.4	80.2	241	51	-71.49	4.31
3gwi	A	Monomer	1.6	6	96.9	170	51	-32.23	-5.78
3my2	A	Monomer	2.2	6.1	77.4	175	51	-0.18	-7.32
1dwk	A	Homo10	1.65	5	112	156	52	-55.93	3.77
1k2x	B	Hetero4	1.65	4.4	86.8	143	52	-80.57	10.58
1k7j	A	Monomer	1.4	6	90.8	206	52	-66.54	15.25
1r9l	A	Monomer	1.59	5.7	73.7	309	52	-96.33	-33.87
2aco	A	Homo2	1.8	9	67.6	173	52	-28.08	-13.23
2o1c	A	Monomer	1.8	5.8	89.1	150	53	-55.72	-3.30
1fua	A	Homo4	1.92	6.1	101.7	215	54	-111.79	-7.02
1k6d	A	Homo2	1.9	5.1	108.1	220	54	-95.19	20.11
1ef8	A	Homo3	1.85	5.7	98.2	261	56	-109.98	14.81
1xnf	A	Homo2	1.98	4.5	94.1	275	56	-41.90	-24.49
2ab0	A	Homo2	1.1	5.8	107.5	205	56	-123.66	12.72
1vhm	A	Homo2	2.1	5.2	102	195	57	-74.90	-1.22
1eq7	A	Homo3	1.9	6	71.6	56	58	-16.65	-1.24
2fsh	A	Homo2	2	5.3	87.8	853	58	-107.63	21.10
2pjp	A	Monomer	2.3	5.5	79.9	121	58	-27.88	-8.27
1or7	C	Hetero2	2	4.7	73.8	90	59	-15.52	-1.43
2qzb	A	Monomer	2.1	4.9	69.3	166	59	-40.35	-2.57
3ffv	A	Monomer	2	4.9	89.5	181	59	-44.07	-18.02
1cs0	B	Hetero8	2	5.9	87.9	382	60	-86.96	3.56
3f1l	A	Homo2	0.95	7.7	82.5	252	60	-114.88	-19.50
3rfa	A	Monomer	2.05	6.6	88.1	404	60	-111.51	-0.41
1hxq	A	Homo2	1.86	6	69.3	348	61	-8.61	-51.11
1k6k	A	Monomer	1.8	5.3	99.6	143	61	-67.12	-5.29
3ucs	C	Hetero4	1.87	6.1	58.1	74	61	-12.96	-3.31
1a99	A	Homo2	2.2	5.5	85.6	344	62	-111.58	-4.41
2gq1	A	Homo4	1.4	5.7	86.3	332	62	-75.12	6.01
3u0o	A	Homo2	2.25	5.3	92	347	62	-153.43	45.39
2xap	A	Homo6	2.1	5.8	87.1	761	63	-204.00	-56.52
2xuv	A	Homo2	1.5	4.9	65.4	79	63	-25.19	-7.97
3o1f	A	Monomer	1.4	5.2	93.8	81	63	-44.62	-1.94
1h5r	A	Homo4	1.9	5.4	92.9	293	64	-108.27	-1.32
2nul	A	Homo2	2.1	5.5	70.1	164	64	-35.25	8.89
4a6v	A	Monomer	1.46	5.6	93.7	265	64	-91.04	26.05
4aq4	A	Monomer	1.8	6.1	62.7	419	64	-81.99	-24.75
4jak	A	Homo2	2	6.6	84.2	167	64	-61.46	-3.63
2hur	A	Homo4	1.62	5.6	81.8	142	65	-51.26	5.77
3f4l	A	Homo2	2	6.1	82.2	345	65	-42.63	1.81
1q2l	A	Monomer	2.2	5.7	82.1	939	66	-181.87	-57.33
3a5y	A	Homo2	1.9	5.6	81.5	345	66	-38.56	-24.19
3qd7	X	Monomer	2.3	8.9	96.1	137	66	-37.39	-18.16
4keh	A	Hetero4	1.9	6.2	88.9	171	66	-66.27	11.02
1i52	A	Monomer	1.5	6.2	93.9	236	67	-111.69	-7.15
1knw	A	Homo2	2.1	5.8	94.3	425	67	-168.59	-13.65

PDB Code	Chain	Bio Unit	Resolution (Å)	pI	AI	Length	Solubility (%)	ΔW [total] (kcal/mol)	ΔW [insol] – ΔW [sol] (kcal/mol)
1q0n	A	Monomer	1.25	5.3	99.4	158	67	-49.61	-9.10
1xeo	A	Monomer	1.3	5.2	112.6	168	67	-29.53	11.30
3qas	A	Homo2	1.7	6.5	83.8	253	67	-97.43	-15.80
1ni9	A	Monomer	2	5.4	102.2	338	69	-170.22	65.90
2d3w	A	Homo4	2.5	4.9	97.8	248	69	-70.84	22.93
4d79	A	Homo2	1.77	8.7	89.1	276	69	-157.41	17.53
1gyn	A	Homo2	2	5.5	85.2	358	70	-157.55	13.00
1hru	A	Homo2	2	4.9	93.9	188	70	-100.57	-3.20
1s5u	A	Homo4	1.7	7.2	85.4	138	70	-25.28	0.27
1xvi	A	Homo2	2.26	5	90.5	275	70	-94.19	-2.76
2yva	A	Homo4	1.85	5.3	101.2	196	70	-111.15	-3.17
1iwl	A	Monomer	1.65	5.6	61.1	182	71	26.82	-20.29
1q08	A	Homo2	1.9	6.4	93.6	99	71	-29.48	-7.28
3grh	A	Monomer	1.7	5.9	72.6	422	71	-76.95	-28.09
3r2q	A	Homo2	1.05	5.1	104.3	202	71	-77.17	-1.39
1fvk	A	Homo2	1.7	5.4	76.3	189	72	-59.62	15.62
1oro	A	Homo2	2.4	5.3	96.7	213	72	-99.46	20.94
1woc	A	Homo4	2	8.5	90.8	103	72	-19.99	-7.85
4wep	A	Monomer	1.5	8	86.2	297	72	-101.27	13.39
1t3d	A	Homo6	2.2	6.5	92.9	289	73	-114.76	6.88
2rn2	A	Monomer	1.48	8.4	73.7	155	73	-37.97	-14.32
3o52	A	Homo2	2.5	4.9	97.4	191	73	-20.34	12.80
4dy3	A	Monomer	1.8	9	62.3	111	73	7.79	2.60
3hi2	B	Hetero2	2	8.8	93.5	101	74	-28.89	4.52
3tch	A	Monomer	1.98	6.1	78.5	524	74	-49.21	-25.34
1eum	A	Homo24	2.05	4.8	75.8	165	75	1.64	-22.83
1pyu	A	Hetero8	1.9	10.9	71.2	41	75	1.67	1.12
1wl9	A	Homo4	1.9	5.3	89.8	440	75	-113.68	6.27
1q8r	A	Homo2	1.9	9.6	79.7	120	76	-36.72	3.15
1qu9	A	Homo3	1.2	5.4	96.8	128	76	-32.84	28.93
1rya	A	Homo2	1.3	5.1	79.8	160	76	-44.32	-20.39
2cx6	A	Monomer	2.43	4.5	93.1	90	76	-37.26	-1.54
2uvk	A	Homo2	1.5	6.7	68.1	357	76	-78.04	-4.56
2x26	A	Monomer	1.75	8.6	92.9	308	76	-112.49	-3.33
3ctl	A	Homo6	2.2	5.3	90.9	231	76	-89.17	16.27
3rer	A	Homo6	1.7	9.7	112.3	65	76	-19.22	2.43
1gsa	A	Homo4	2	5.1	93.5	316	77	-73.19	29.26
1hpu	A	Monomer	1.85	5.4	78	525	77	-68.89	26.59
1psu	A	Homo2	2.2	6.3	76.1	140	77	-66.93	-3.35
1rrm	A	Homo2	1.6	5	95.1	386	77	-215.44	51.75
1s16	A	Monomer	2.1	5.8	88.2	390	77	-113.06	3.10
1sff	A	Homo4	1.9	5.8	95.8	426	77	-201.67	51.46
3qmq	A	Homo2	2.2	5.7	76.7	99	77	1.77	-3.07
1eua	A	Homo3	1.95	5.6	106.8	213	78	-173.74	32.57
1zgz	A	Homo2	1.8	5	118.1	122	78	-53.36	5.14
2g2n	A	Homo4	1.65	8.2	77.8	114	78	-13.15	-6.99
2oml	A	Monomer	1.2	9.6	85.1	189	78	-40.84	-11.28
4k49	A	Homo2	1.89	7	96	136	78	-57.08	-6.72
1n57	A	Homo2	1.6	5.9	80.2	291	79	-85.67	7.02
1ujc	A	Monomer	1.9	4.5	99.3	161	79	-76.19	-1.52
1yrr	A	Homo4	2	5.7	100.4	382	79	-179.25	29.32
3csu	A	Homo3	1.88	6.1	98.2	310	79	-94.49	-8.53
4cnd	A	Homo2	1.5	6.4	93.9	267	79	-123.13	18.32
4kdc	A	Monomer	2.09	6.4	85.2	246	79	-97.00	6.69
4qak	A	Monomer	2.03	10.5	85	177	79	-54.14	-23.94

PDB Code	Chain	Bio Unit	Resolution (Å)	pI	AI	Length	Solubility (%)	ΔW ^{total} (kcal/mol)	ΔW ^{insol} – ΔW ^{sol} (kcal/mol)
1ixh	A	Monomer	0.98	6.9	80.6	321	80	-136.43	12.17
1q5y	A	Homo4	1.4	5.9	92.8	85	80	-8.35	-9.10
4qus	A	Homo2	1.28	4.8	90.9	149	80	-29.96	3.42
1b79	A	Homo4	2.3	4.8	86.1	119	81	-47.57	-8.11
1jgs	A	Homo2	2.3	8.6	122.2	138	81	-65.73	-1.56
1qvc	A	Homo4	2.2	9.1	64.5	145	81	-14.46	9.22
1rxy	A	Homo6	1.7	5.8	89.5	253	81	-124.80	16.91
1yoe	A	Homo4	1.78	5.3	98.7	322	81	-155.17	19.51
2eby	A	Homo2	2.25	5.7	99.3	113	81	-50.06	2.60
2hqs	C	Hetero2	1.5	6.8	77.7	118	81	-68.01	-1.89
4ej1	A	Hetero2	1.75	4.8	84.7	159	81	-47.48	-8.55
5isv	A	Monomer	1.35	5.5	85.9	165	81	-32.84	-2.30
1c0a	A	Monomer	2.4	5.5	87.4	585	82	-131.60	23.93
1eej	A	Homo2	1.9	5.9	84.5	216	82	-74.21	11.98
1i7h	A	Homo4	1.7	4.5	87	111	82	-43.76	-2.99
1k0e	A	Monomer	2	5.1	87.7	453	82	-102.96	-32.12
1qy9	A	Homo2	2.05	5.8	92.3	297	82	-118.59	4.41
1spv	A	Monomer	2	5.9	93.9	184	82	-128.29	1.45
2ane	A	Homo8	2.03	6.1	99.8	125	82	-37.02	16.68
1a6j	A	Homo4	2.35	5.6	103.6	163	83	-74.46	3.71
2h28	A	Monomer	2.1	6.1	78.1	130	83	-38.10	-15.24
4eac	A	Monomer	2.3	5.8	81.3	414	83	-63.95	-3.31
4q2u	A	Hetero4	1.8	5.2	107.9	86	83	-18.38	9.35
1fm0	E	Hetero2	1.45	5.3	73.5	150	84	-49.67	5.70
1npd	A	Homo2	2.3	5	88.5	288	84	-115.09	14.43
1u2k	A	Monomer	2	4.8	93.1	309	84	-119.94	9.46
2a6q	A	Hetero3	2.05	5.3	84.1	86	84	-26.82	3.26
3cqd	A	Homo4	1.98	5.3	99.8	309	84	-191.22	41.28
4jhc	A	Homo2	1.85	6.3	85.8	215	84	-29.78	0.44
1n8o	E	Hetero6	2	5.9	85.7	142	85	-7.04	15.16
1onr	A	Monomer	1.87	5.1	99.8	316	85	-117.48	15.29
1dfu	P	Monomer	1.8	9.6	86.2	94	86	-18.06	9.66
2e3d	A	Homo4	1.95	5.1	102.6	302	86	-93.09	28.61
2p2d	A	Homo4	1.89	6	87.2	358	86	-134.06	2.53
2pv2	A	Homo2	1.3	5.8	97.7	103	86	-54.22	2.83
1abe	A	Monomer	1.7	5.6	81.9	306	87	-112.57	37.67
1gvh	A	Homo12	2.19	5.5	79.9	396	87	-89.42	-22.76
1opc	A	Monomer	1.95	7	78.9	110	87	-27.18	-0.35
2yh5	A	Monomer	1.25	5.7	76.8	127	87	-44.80	-1.93
1g8e	A	Homo2	1.8	6.5	107.7	116	88	-43.87	-6.17
1oms	A	Homo3	2.3	5.9	91.7	121	88	-31.09	10.80
1poh	A	Monomer	2	5.6	84.9	85	88	-37.24	9.26
1qfj	A	Monomer	2.2	5.3	93.8	232	88	-107.66	3.74
1tq5	A	Monomer	1.76	5.7	78.6	242	88	27.42	-16.95
1cmc	A	Homo2	1.8	5.4	78.9	104	89	10.46	-4.54
1k4m	A	Homo3	1.9	5.5	92.1	213	89	-71.95	-9.80
1qyn	A	Homo4	2.35	4.3	78.4	153	89	-58.05	-2.95
1r62	A	Monomer	1.6	5.7	103	160	89	-44.46	9.24
2dy0	A	Homo2	1.25	5.3	104.2	190	89	-88.28	31.35
2trx	A	Monomer	1.68	4.7	104	108	89	-44.65	13.41
4zck	A	Monomer	1.48	7.9	78.6	332	89	-44.43	7.68
2vk2	A	Monomer	1.2	6.3	85.5	306	90	-83.17	48.52
5i4c	A	Homo2	2	5.6	119.4	147	90	-71.14	24.84
1i8d	A	Homo2	2	5.6	95.6	213	91	-50.98	30.53
1kae	A	Homo2	1.7	5.1	93.4	434	91	-214.48	31.81

PDB Code	Chain	Bio Unit	Resolution (Å)	pI	AI	Length	Solubility (%)	ΔW total (kcal/mol)	ΔW insol – ΔW sol (kcal/mol)
1moq	A	Homo2	1.57	5.3	105	368	91	-143.61	18.44
1xs1	A	Homo3	1.8	5.6	97.1	193	91	-33.67	4.74
1xxa	A	Homo6	2.2	4.1	123.9	78	91	-41.14	14.24
2wci	A	Homo4	1.9	5.9	79.6	135	91	-60.16	-14.82
3qn0	A	Homo6	2.34	6.6	76	141	91	-31.83	4.07
1ega	A	Homo2	2.4	6.7	100.1	301	92	-86.38	28.68
1hnj	A	Homo2	1.46	5.1	96.1	317	92	-191.90	21.70
2bh8	A	Homo4	1.9	6.6	70.5	101	92	-11.97	8.29
3pvs	A	Homo4	2.5	6	83.3	447	92	-156.02	-2.63
1faj	A	Homo6	2.15	5	91.4	175	93	-39.61	23.01
1gz0	A	Homo2	2.5	6.5	103	253	93	-126.92	5.42
2gmw	A	Monomer	1.5	5.8	79.1	211	93	-63.75	2.27
3i96	A	Homo6	1.65	5.9	105.8	119	93	-85.68	20.36
4arc	A	Monomer	2	5.4	77.8	880	93	-189.65	-17.45
1d8l	A	Homo4	2.5	6.3	107.9	149	94	-70.34	11.36
1o8b	A	Homo2	1.25	5.2	106.9	219	94	-72.33	38.75
1pdo	A	Homo2	1.7	4.5	103.3	135	94	-81.26	22.38
1qjc	A	Homo6	1.63	6.5	92.7	158	94	-63.97	4.11
1x8d	A	Homo2	1.8	5.3	76	104	94	-9.85	-18.10
1bdo	A	Homo2	1.8	4.6	94.9	80	95	-24.25	17.62
1hv9	A	Homo3	2.1	6.1	98.8	456	95	-185.87	69.13
1k7k	A	Monomer	1.5	5.9	86.6	221	95	-71.06	7.89
1kid	A	Monomer	1.7	5.7	100	203	95	-93.59	44.06
1ptm	A	Homo2	1.96	5.9	108.5	329	95	-167.73	16.20
3bmb	A	Monomer	1.91	4.5	105.4	136	95	-62.25	9.30
3c8f	A	Monomer	2.25	6	79.8	245	95	-75.39	-1.07
1anf	A	Monomer	1.67	5.2	83.4	370	96	-137.08	24.33
1xsq	A	Homo2	1.6	5.3	95.7	168	96	-10.14	-7.48
1y2g	A	Monomer	1.9	5.4	84.3	140	96	-39.16	5.92
2y3q	A	Homo24	1.55	4.7	104.9	158	96	6.45	-3.02
1euw	A	Homo3	1.05	5	95.6	152	97	-44.00	-1.91
1iov	A	Monomer	2.2	4.8	98.5	306	97	-143.07	9.41
3bb6	A	Homo4	2.3	6.3	72.2	127	97	-6.19	-5.48
3lvk	B	Hetero4	2.44	5.2	80.9	82	97	-14.06	8.55
4mva	A	Homo2	1.43	5.9	90.4	279	97	-131.36	30.61
1jkx	A	Homo2	1.6	5.5	94.9	212	98	-101.63	6.12
1np6	A	Homo2	1.9	5.3	104.3	174	98	-70.87	22.15
3g27	A	Homo2	2.1	8.3	87.5	96	98	-30.43	-12.77
4nfw	A	Monomer	2.3	5	86.7	153	98	-41.01	-16.91
4p1m	A	Homo4	1.95	6.3	78.6	123	98	-34.29	0.01
1ag9	A	Homo2	1.8	4.2	87.6	175	99	-72.42	1.56
1amf	A	Monomer	2.15	6.4	88.4	233	99	-115.48	19.60
1ctf	A	Homo2	1.7	5	101.8	74	99	-42.46	22.96
1u9l	A	Monomer	1.9	4.1	104.7	70	99	-41.79	9.38
1f9z	A	Homo2	1.5	5	86.7	135	100	-33.99	12.94
1gg1	A	Homo4	2	6.1	94.8	350	100	-138.36	11.46
1zmr	A	Monomer	2.4	5.1	102.6	387	100	-225.31	58.25
1vlo	A	Monomer	1.7	5.8	83.3	381	101	-92.75	19.80
1x6i	A	Homo2	1.2	5.5	80.4	91	101	-8.90	-8.34
2h27	A	Monomer	2.3	5.8	108.2	73	101	-35.40	1.31
3n3a	C	Hetero4	1.99	9.6	79	153	101	-63.92	-6.33
1h75	A	Monomer	1.7	7.9	74.8	81	103	-40.44	-6.08
2bz1	A	Homo2	1.54	5.6	99.5	196	103	-52.82	1.11
1mzg	A	Homo2	2	6.7	100.3	146	104	-47.85	-13.97
2igi	A	Homo2	1.7	5	91.1	180	104	-32.42	3.26

PDB Code	Chain	Bio Unit	Resolution (Å)	pI	AI	Length	Solubility (%)	ΔW ^{total} (kcal/mol)	ΔW ^{insol} – ΔW ^{sol} (kcal/mol)
4mte	A	Homo4	2.5	6	87.3	171	104	-60.27	-3.39
1ake	A	Monomer	2	5.6	89.3	214	105	-52.20	23.78
1r2k	A	Homo2	2.1	5.7	86	169	105	-63.68	-6.06
2wcv	A	Homo10	1.9	5.6	110.9	140	105	-62.06	15.67
3itf	A	Homo2	1.45	6.2	59.9	145	105	-8.48	-7.53
1nmn	A	Monomer	2.3	6.7	90.5	138	106	-37.65	11.57
4ml2	A	Monomer	1.5	9.5	88.3	95	107	0.04	0.64
1dj8	A	Homo2	2	4.7	67	89	108	-28.59	10.53
1k4n	A	Monomer	1.6	5.4	92.5	192	108	-60.55	-12.71
3a6s	A	Monomer	1.8	5	83.2	129	108	-23.47	10.02
1v4b	A	Homo2	1.8	5.1	101.5	200	109	-86.79	5.64
2gq0	A	Monomer	1.9	5.6	81.2	303	109	-40.53	-19.85
3a5z	B	Hetero4	2.5	5	80.6	191	109	-5.65	1.22
1fia	A	Homo2	2	9.3	86.4	98	110	-18.49	1.08
2fu4	A	Homo2	1.8	5.5	101	83	110	-35.88	4.74
4dt4	A	Monomer	1.35	4.9	82	169	110	-51.25	16.33
1b93	A	Homo2	1.9	6.1	104.6	152	112	-77.79	1.41
1fjj	A	Homo2	1.66	5.3	73.6	159	112	-49.79	-25.98
1cfz	A	Monomer	2.2	4.6	124	162	113	-103.04	33.99
1ew4	A	Monomer	1.4	4.2	79.2	106	113	-26.86	-19.92
1f3z	A	Homo2	1.98	4.7	108.8	161	114	-56.04	42.44
1s96	A	Homo2	2	6.4	83.3	219	115	-46.71	-6.83
3dpo	A	Homo2	2.1	5.1	87.9	219	116	-15.00	38.04
2air	B	Hetero4	2	6.9	98.1	153	117	-32.97	1.20
1tuv	A	Homo2	1.7	6.4	89.8	114	121	-28.01	4.28
1scz	A	Homo24	2.2	8.7	97.4	233	122	-65.86	14.97
4ynx	A	Homo2	1.5	9.8	81.4	66	130	2.91	8.34
1jyh	A	Monomer	1.8	4.6	74.4	157	133	-28.68	6.06

Table S2. Insolubilizing residue-residue interactions, defined by $\mathcal{M} < 0$ and the relaxed significance criteria requiring the $|\mathcal{M}|$ or $\mathcal{V}$ values to be higher than 95% of the equivalent quantities computed from randomly shuffled datasets (Sig$\mathcal{M}$ or Sig$\mathcal{V} \geq 0.95$).

**Interactions**	**Residue pairs**	$\mathcal{M}$	Sig$\mathcal{M}$	$\mathcal{V}$	Sig$\mathcal{V}$
π-π	TRP-PHE	-0.207	1	0.052	1
	TYR-TRP	-0.177	1	0.038	0.99
	TRP-TRP	-0.412	0.99	0.181	0.99
	TYR-PHE	-0.124	0.97	0.019	0.99
His-π	HIS-TYR	-0.155	1	0.038	0.99
	HIS-TRP	-0.191	0.99	0.063	1
	HIS-PHE	-0.122	0.96	0.022	0.95
Cation-π	ARG-TRP	-0.238	1	0.074	1
	ARG-PHE	-0.120	0.99	0.017	0.99
	ARG-TYR	-0.101	0.98	0.017	0.98
	LYS-TRP	-0.162	0.97	0.068	0.98
Amino-π	GLN -TRP	-0.359	1	0.135	1
	GLN-PHE	-0.128	1	0.028	1
	ASN-PHE	-0.140	1	0.024	0.99
	ASN-TRP	-0.183	1	0.044	0.98
	GLN-TYR	-0.141	0.99	0.024	0.95
Anion-π	ASP-TRP	-0.211	1	0.049	1
	ASP-TYR	-0.103	0.96	0.012	0.91
Aromatic-containing	TRP-SER	-0.294	1	0.104	1
	PHE-CYS	-0.232	1	0.062	1
	TRP-ALA	-0.206	1	0.048	1
	TRP-PRO	-0.205	1	0.045	1
	TYR-SER	-0.129	1	0.021	1
	TRP-LEU	-0.192	1	0.037	1
	TRP-GLY	-0.153	0.99	0.033	0.98
	TRP-CYS	-0.267	0.99	0.076	0.97
	TYR-GLY	-0.109	0.98	0.021	0.97
	TRP-ILE	-0.114	0.97	0.024	0.98
	TRP-VAL	-0.131	0.97	0.018	0.93
	TRP-MET	-0.197	0.95	0.048	0.94
	TYR-LEU	-0.062	0.95	0.005	0.86
	TYR-ALA	-0.024	0.52	0.015	1
His-containing	HIS-ALA	-0.108	1	0.016	0.98
	HIS-PRO	-0.124	0.99	0.021	0.97
	HIS-LEU	-0.110	0.97	0.027	0.99
Arg-containing	ARG-SER	-0.152	1	0.025	1
	ARG-ARG	-0.184	0.99	0.036	0.99
	ARG-PRO	-0.128	0.99	0.030	0.99
	ARG-LEU	-0.084	0.99	0.008	0.96
	ARG-CYS	-0.230	0.98	0.062	0.98
	ARG-GLY	-0.096	0.98	0.011	0.93
	ARG-GLN	-0.166	1	0.033	1
	ARG-ASN	-0.120	0.99	0.023	1
Asn/Gln-containing	ASN-GLN	-0.158	0.99	0.032	0.99
	GLN-CYS	-0.152	0.95	0.051	1
	ASN-CYS	-0.167	0.95	0.044	0.94
	GLN-ALA	-0.085	0.95	0.010	0.88
	ASN-SER	-0.087	0.95	0.009	0.8
	GLN-GLY	-0.081	0.95	0.010	0.8
	GLN-LEU	-0.065	0.94	0.010	0.98
	ASN-LEU	-0.009	0.17	0.010	0.97
Miscellaneous	LEU-CYS	-0.195	1	0.050	1
	LEU-SER	-0.074	0.97	0.010	0.97
	SER-SER	-0.109	0.96	0.019	0.95
	PRO-SER	-0.080	0.96	0.009	0.91
	CYS-PRO	-0.134	0.95	0.029	0.9
	ASP-LEU	-0.033	0.77	0.010	0.97
	SER-GLY	-0.004	0.11	0.010	0.97

Table S3. Solubilizing residue-residue interactions, defined by $\mathcal{M} > 0$ and the relaxed significance criteria requiring the $|\mathcal{M}|$ or $\mathcal{V}$ values to be higher than 95% of the equivalent quantities computed from randomly shuffled datasets (Sig$\mathcal{M} \geq 0.95$ or Sig$\mathcal{V} \geq 0.95$).

**Interactions**	**Residue pairs**	$\mathcal{M}$	Sig$\mathcal{M}$	$\mathcal{V}$	Sig$\mathcal{V}$
Lys-salt bridges	GLU-LYS	0.115	1	0.017	1
	LYS-ASP	0.105	0.97	0.013	0.96
Aliphatic-aliphatic	VAL-VAL	0.156	1	0.025	1
	ILE-ILE	0.125	1	0.018	1
	VAL-ILE	0.096	1	0.010	1
	GLY-VAL	0.114	1	0.015	1
	ILE-ALA	0.072	1	0.006	0.97
	LEU-ILE	0.064	0.99	0.007	1
	LEU-VAL	0.058	0.99	0.004	0.96
	GLY-GLY	0.113	0.98	0.014	0.96
	ALA-VAL	0.060	0.97	0.004	0.94
	GLY-ALA	0.063	0.96	0.005	0.93
Aliphatic-containing	ILE-LYS	0.134	1	0.026	1
	VAL-GLU	0.120	1	0.017	1
	VAL-THR	0.086	1	0.010	0.99
	GLY-ASP	0.114	1	0.017	0.99
	ILE-THR	0.080	1	0.008	0.97
	GLY-THR	0.093	0.99	0.015	0.99
	GLY-GLU	0.105	0.99	0.012	0.96
	ILE-GLU	0.089	0.99	0.011	0.95
	ALA-LYS	0.095	0.98	0.013	0.97
	VAL-PRO	0.068	0.95	0.008	0.98
	VAL-LYS	0.097	0.95	0.014	0.98
	GLY-LYS	0.088	0.95	0.012	0.91
Asp/Glu containing	GLU-THR	0.153	1	0.032	1
	GLU-ASP	0.112	1	0.015	0.9
	ASP-MET	0.124	0.93	0.039	1

Figure S1. Distribution of solubility values $\mathcal{S}$ (in %) of the proteins from the $\mathcal{D}^{\text{tot}}$ dataset. The vertical line at $\mathcal{S} = 64\%$ separates this set into the subset $\mathcal{D}^{\text{sol}}$ of soluble proteins (right) and the set $\mathcal{D}^{\text{insol}}$ of aggregation-prone proteins (left).

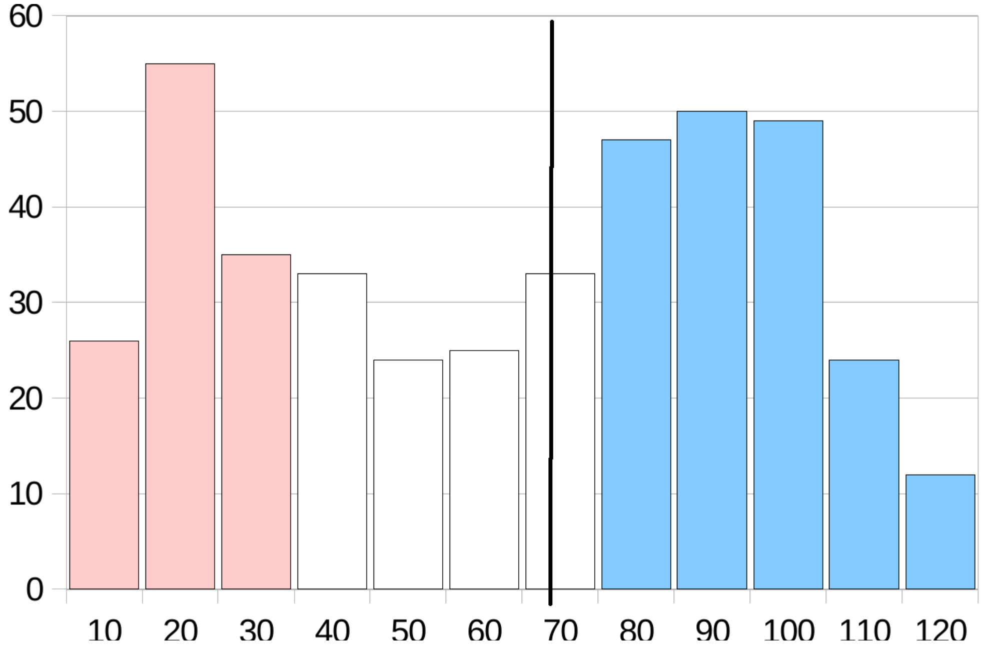

Figure S2. Ratio of the frequency of each amino acid in the set $\mathcal{D}^{\text{insol}}$ over the frequency in the set $\mathcal{D}^{\text{sol}}$, minus 1.

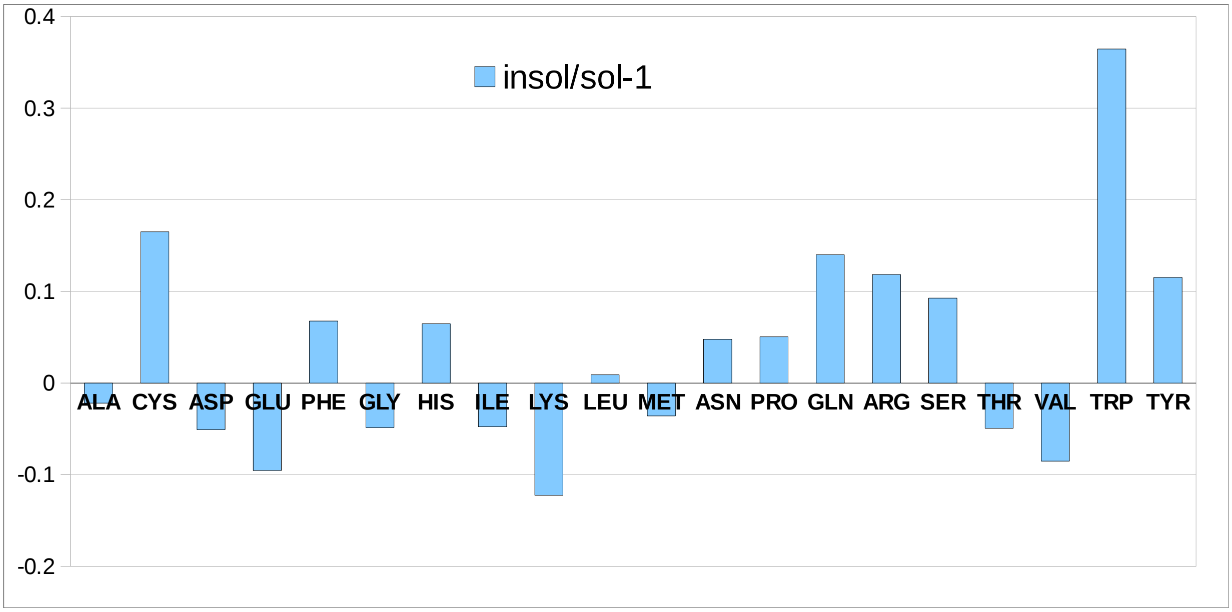

Figure S3. Statistical distance potentials derived from soluble or aggregation-prone protein sets. The energies are in kcal/mol, the distance d (in Å) is computed between residue side chain centroids, and the residues are separated by at least 8 residues along the chain. Distance bins containing ten occurrences or less are not drawn.

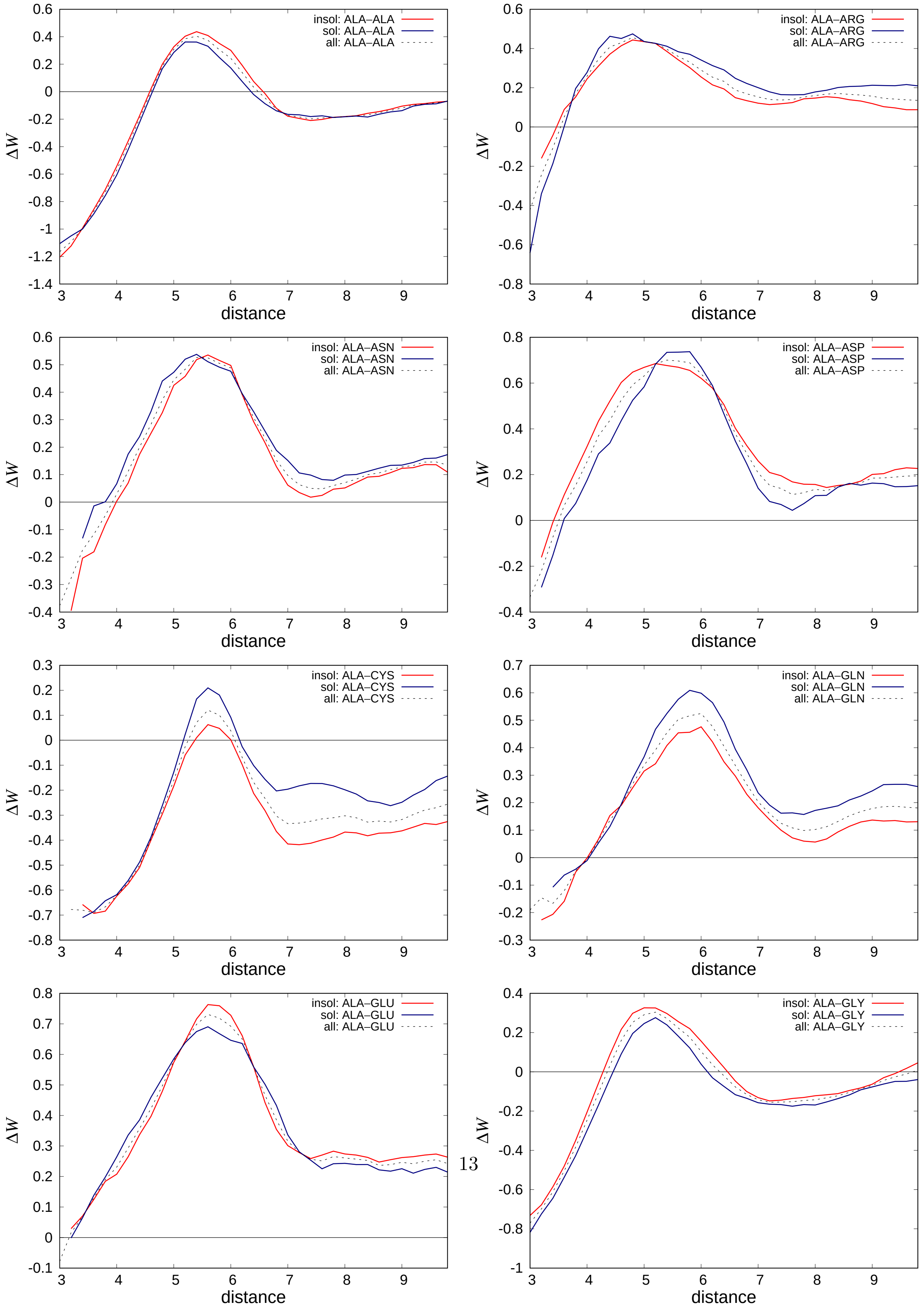

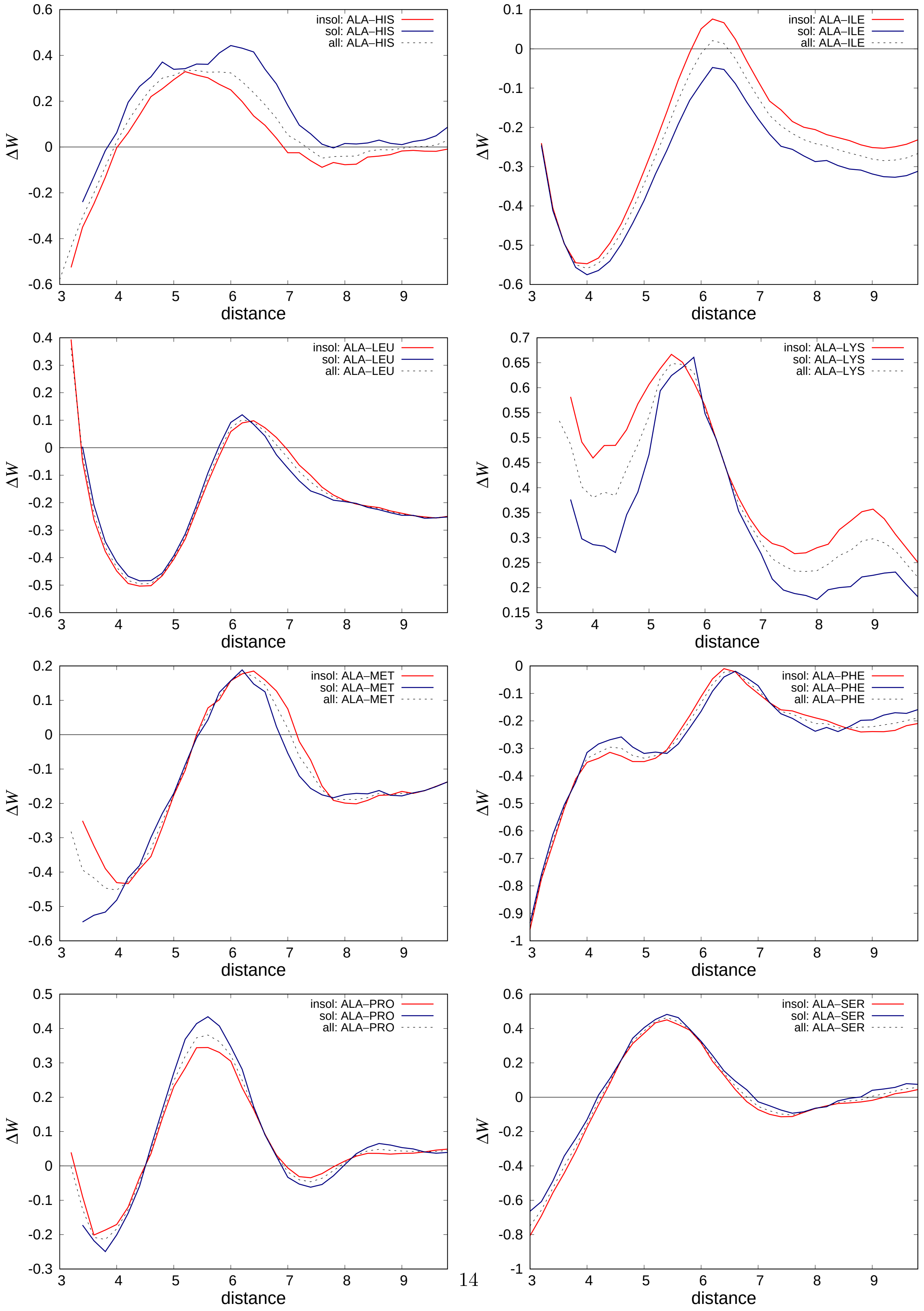
insol: ALA–HIS
sol: ALA–HIS
all: ALA–HIS
insol: ALA–ILE
sol: ALA–ILE
all: ALA–ILE
insol: ALA–LEU
sol: ALA–LEU
all: ALA–LEU
insol: ALA–LYS
sol: ALA–LYS
all: ALA–LYS
insol: ALA–MET
sol: ALA–MET
all: ALA–MET
insol: ALA–PHE
sol: ALA–PHE
all: ALA–PHE
insol: ALA–PRO
sol: ALA–PRO
all: ALA–PRO
insol: ALA–SER
sol: ALA–SER
all: ALA–SER
ΔW
distance

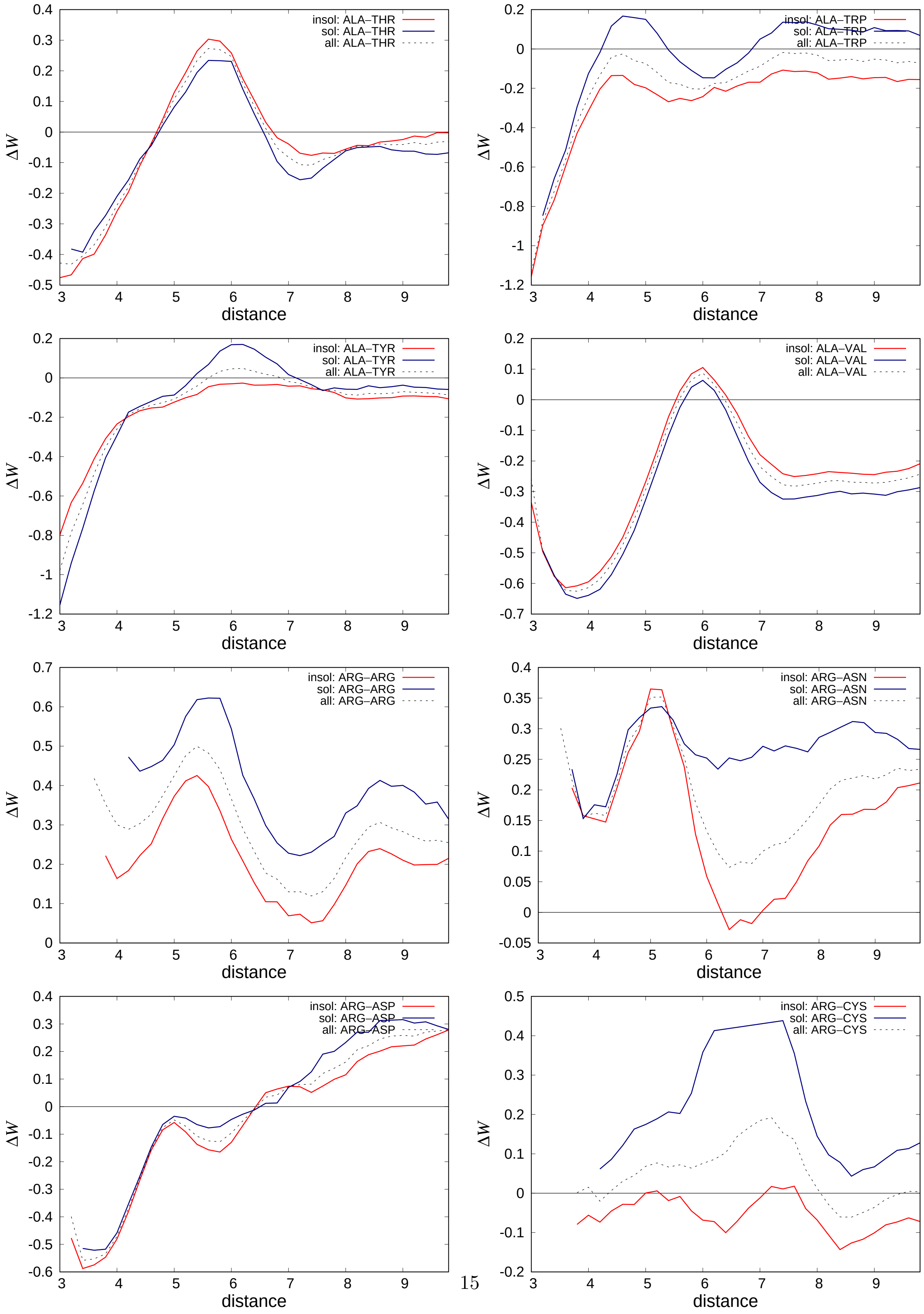
insol: ALA–THR
sol: ALA–THR
all: ALA–THR
insol: ALA–TRP
sol: ALA–TRP
all: ALA–TRP
insol: ALA–TYR
sol: ALA–TYR
all: ALA–TYR
insol: ALA–VAL
sol: ALA–VAL
all: ALA–VAL
insol: ARG–ARG
sol: ARG–ARG
all: ARG–ARG
insol: ARG–ASN
sol: ARG–ASN
all: ARG–ASN
insol: ARG–ASP
sol: ARG–ASP
all: ARG–ASP
insol: ARG–CYS
sol: ARG–CYS
all: ARG–CYS
ΔW
distance

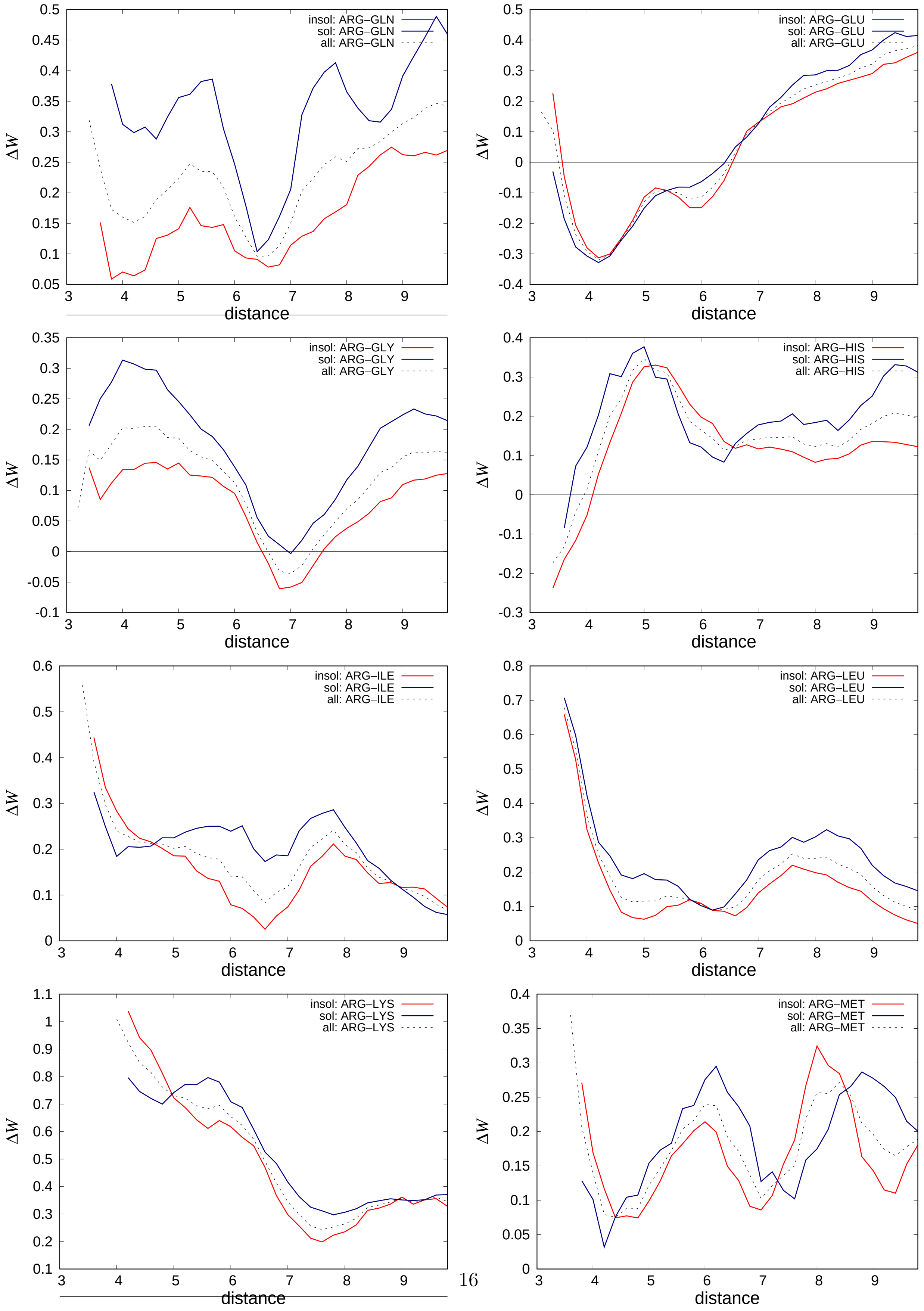
insol: ARG–GLN
sol: ARG–GLN
all: ARG–GLN
insol: ARG–GLU
sol: ARG–GLU
all: ARG–GLU
insol: ARG–GLY
sol: ARG–GLY
all: ARG–GLY
insol: ARG–HIS
sol: ARG–HIS
all: ARG–HIS
insol: ARG–ILE
sol: ARG–ILE
all: ARG–ILE
insol: ARG–LEU
sol: ARG–LEU
all: ARG–LEU
insol: ARG–LYS
sol: ARG–LYS
all: ARG–LYS
insol: ARG–MET
sol: ARG–MET
all: ARG–MET
ΔW
distance

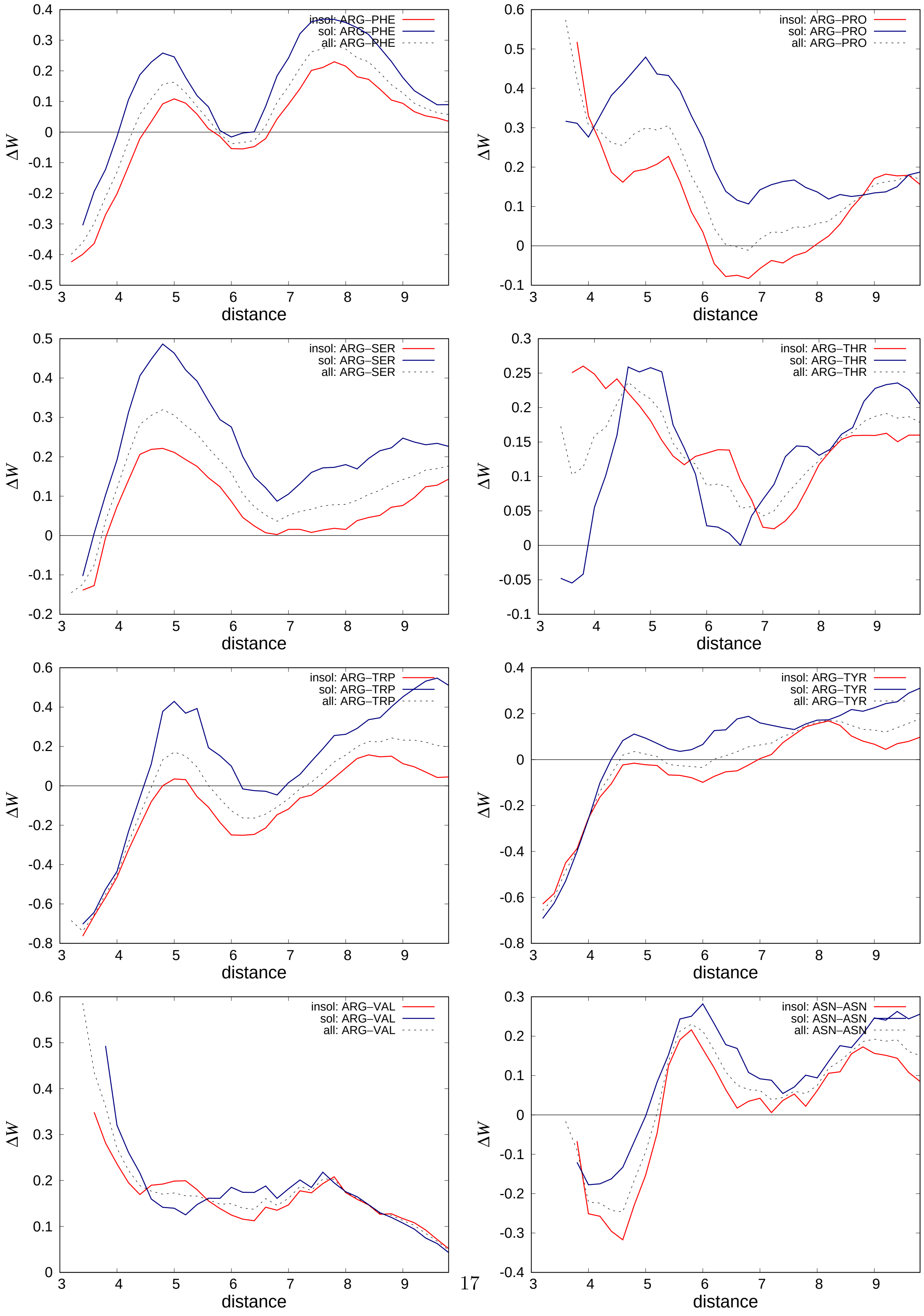
insol: ARG–PHE
sol: ARG–PHE
all: ARG–PHE
insol: ARG–PRO
sol: ARG–PRO
all: ARG–PRO
insol: ARG–SER
sol: ARG–SER
all: ARG–SER
insol: ARG–THR
sol: ARG–THR
all: ARG–THR
insol: ARG–TRP
sol: ARG–TRP
all: ARG–TRP
insol: ARG–TYR
sol: ARG–TYR
all: ARG–TYR
insol: ARG–VAL
sol: ARG–VAL
all: ARG–VAL
insol: ASN–ASN
sol: ASN–ASN
all: ASN–ASN
ΔW
distance

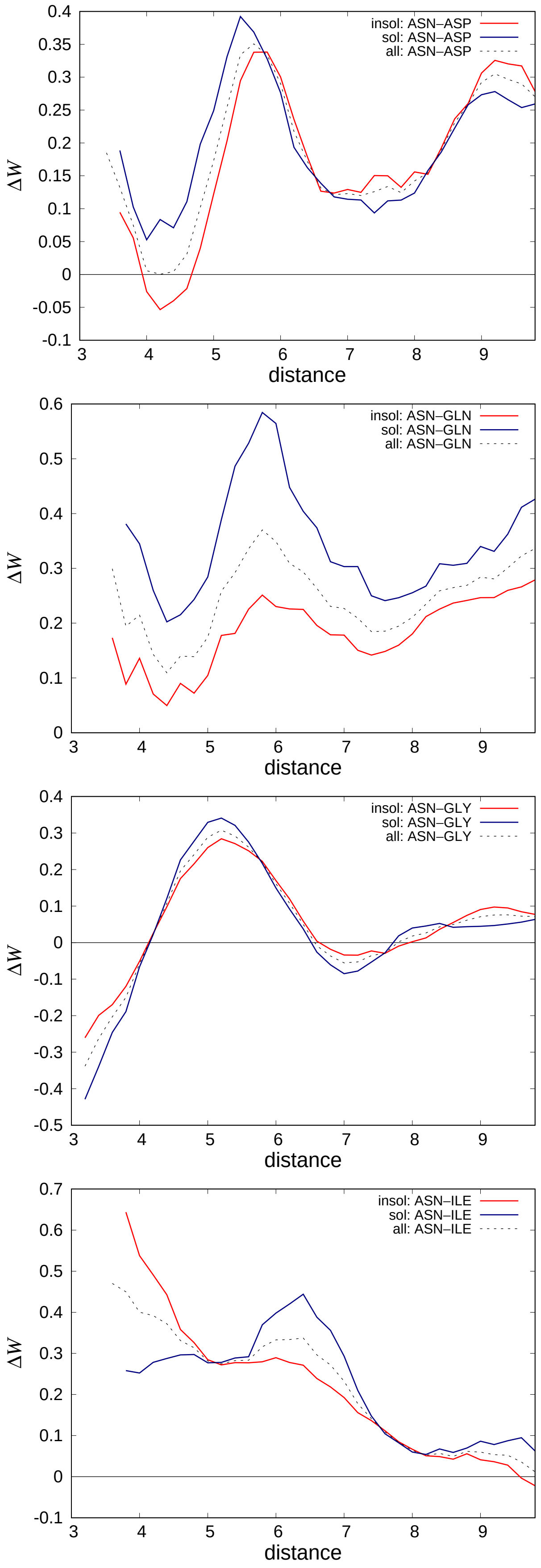
insol: ASN–ASP
sol: ASN–ASP
all: ASN–ASP
insol: ASN–GLN
sol: ASN–GLN
all: ASN–GLN
insol: ASN–GLY
sol: ASN–GLY
all: ASN–GLY
insol: ASN–ILE
sol: ASN–ILE
all: ASN–ILE
ΔW
distance

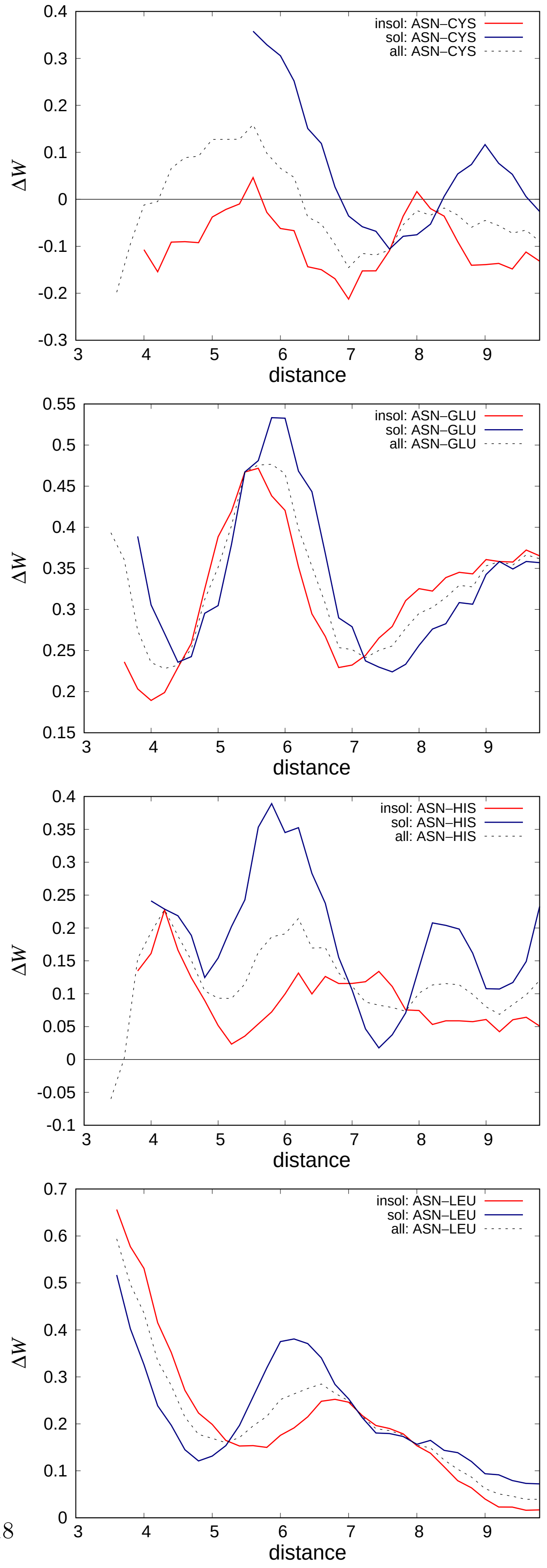
insol: ASN–CYS
sol: ASN–CYS
all: ASN–CYS
insol: ASN–GLU
sol: ASN–GLU
all: ASN–GLU
insol: ASN–HIS
sol: ASN–HIS
all: ASN–HIS
insol: ASN–LEU
sol: ASN–LEU
all: ASN–LEU
ΔW
distance

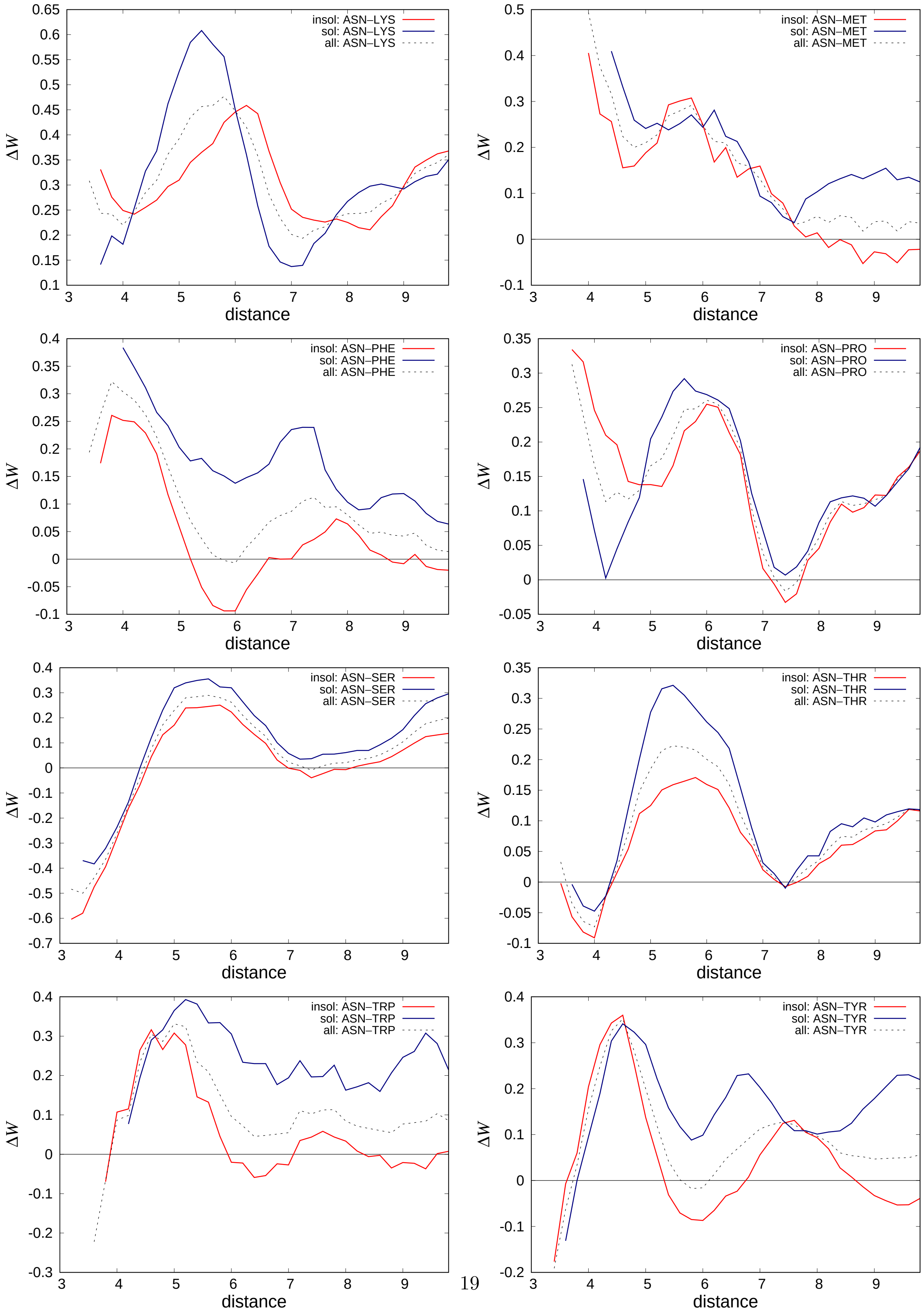
insol: ASN–LYS
sol: ASN–LYS
all: ASN–LYS
insol: ASN–MET
sol: ASN–MET
all: ASN–MET
insol: ASN–PHE
sol: ASN–PHE
all: ASN–PHE
insol: ASN–PRO
sol: ASN–PRO
all: ASN–PRO
insol: ASN–SER
sol: ASN–SER
all: ASN–SER
insol: ASN–THR
sol: ASN–THR
all: ASN–THR
insol: ASN–TRP
sol: ASN–TRP
all: ASN–TRP
insol: ASN–TYR
sol: ASN–TYR
all: ASN–TYR
ΔW
distance

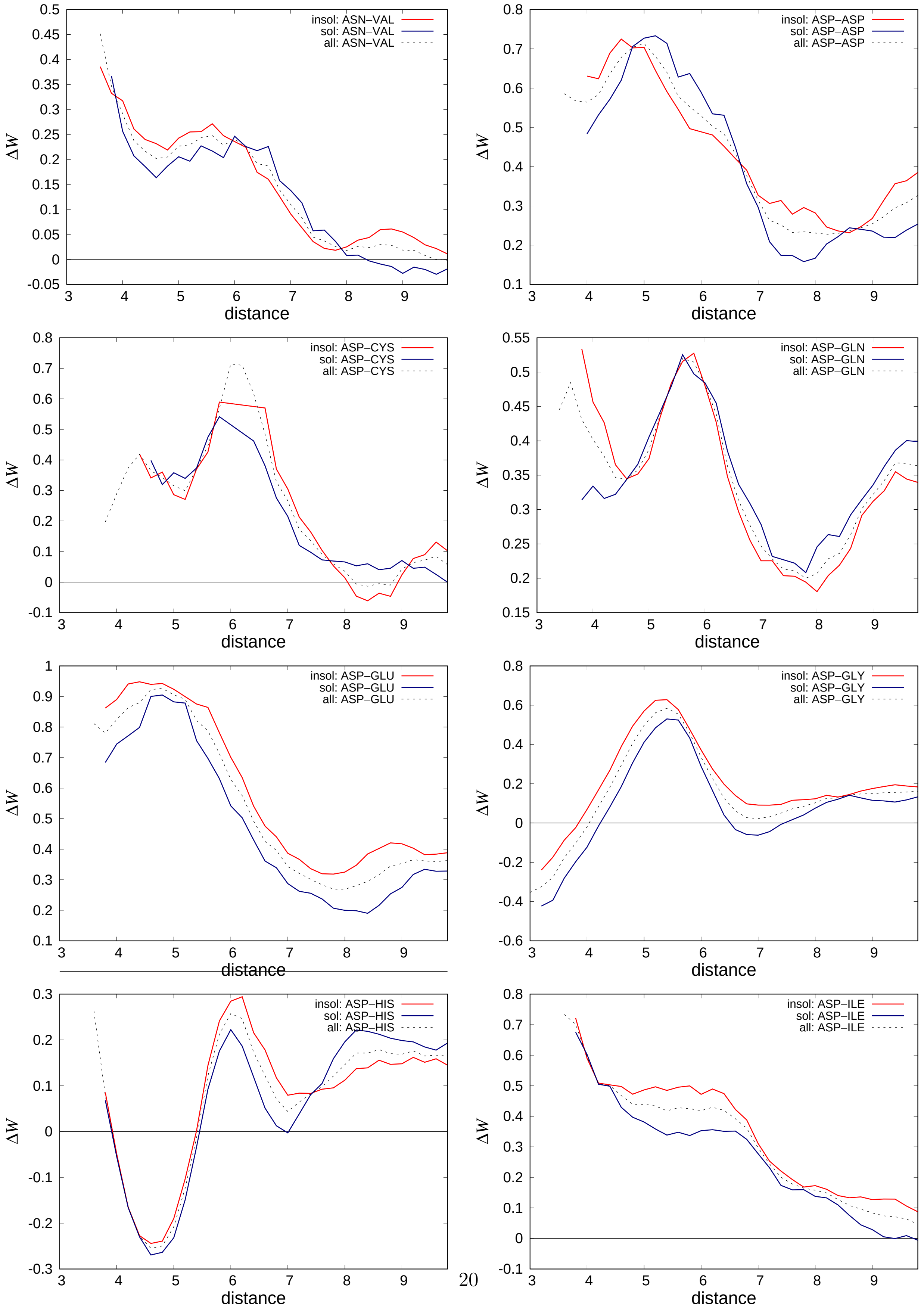
insol: ASN–VAL
sol: ASN–VAL
all: ASN–VAL
insol: ASP–ASP
sol: ASP–ASP
all: ASP–ASP
insol: ASP–CYS
sol: ASP–CYS
all: ASP–CYS
insol: ASP–GLN
sol: ASP–GLN
all: ASP–GLN
insol: ASP–GLU
sol: ASP–GLU
all: ASP–GLU
insol: ASP–GLY
sol: ASP–GLY
all: ASP–GLY
insol: ASP–HIS
sol: ASP–HIS
all: ASP–HIS
insol: ASP–ILE
sol: ASP–ILE
all: ASP–ILE
ΔW
distance

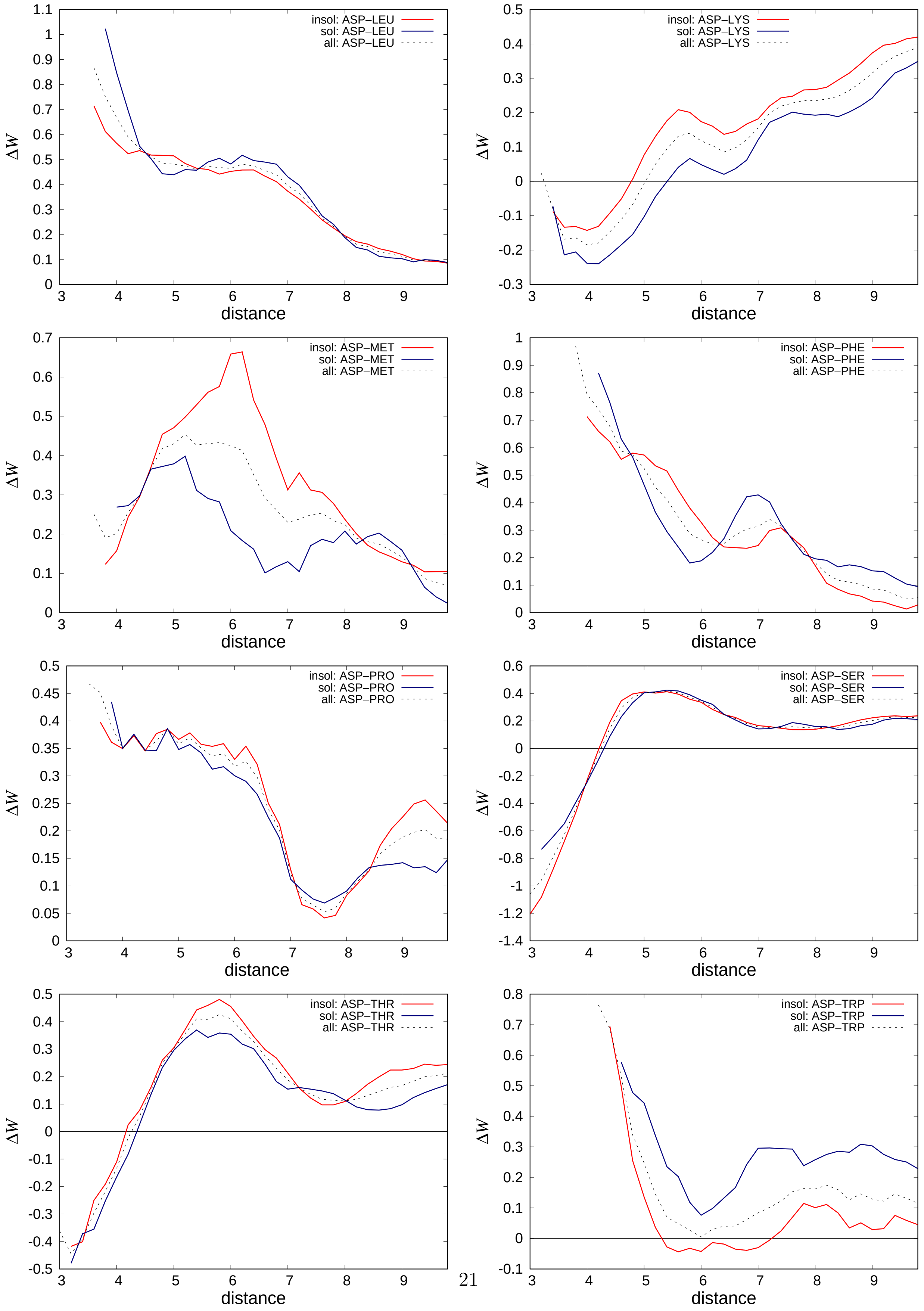
insol: ASP–LEU
sol: ASP–LEU
all: ASP–LEU
insol: ASP–LYS
sol: ASP–LYS
all: ASP–LYS
insol: ASP–MET
sol: ASP–MET
all: ASP–MET
insol: ASP–PHE
sol: ASP–PHE
all: ASP–PHE
insol: ASP–PRO
sol: ASP–PRO
all: ASP–PRO
insol: ASP–SER
sol: ASP–SER
all: ASP–SER
insol: ASP–THR
sol: ASP–THR
all: ASP–THR
insol: ASP–TRP
sol: ASP–TRP
all: ASP–TRP
ΔW
distance

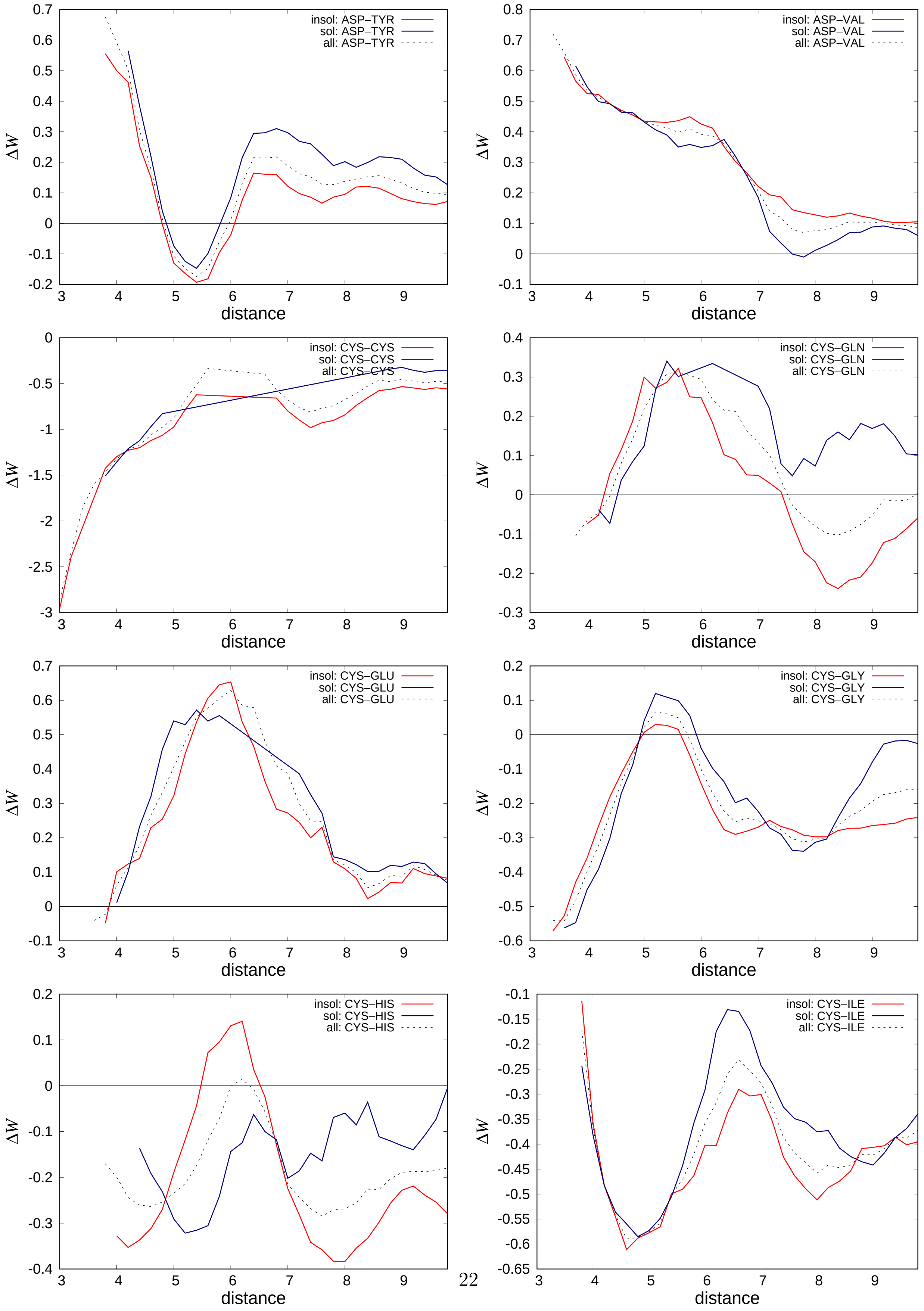
insol: ASP–TYR
sol: ASP–TYR
all: ASP–TYR
insol: ASP–VAL
sol: ASP–VAL
all: ASP–VAL
insol: CYS–CYS
sol: CYS–CYS
all: CYS–CYS
insol: CYS–GLN
sol: CYS–GLN
all: CYS–GLN
insol: CYS–GLU
sol: CYS–GLU
all: CYS–GLU
insol: CYS–GLY
sol: CYS–GLY
all: CYS–GLY
insol: CYS–HIS
sol: CYS–HIS
all: CYS–HIS
insol: CYS–ILE
sol: CYS–ILE
all: CYS–ILE
ΔW
distance

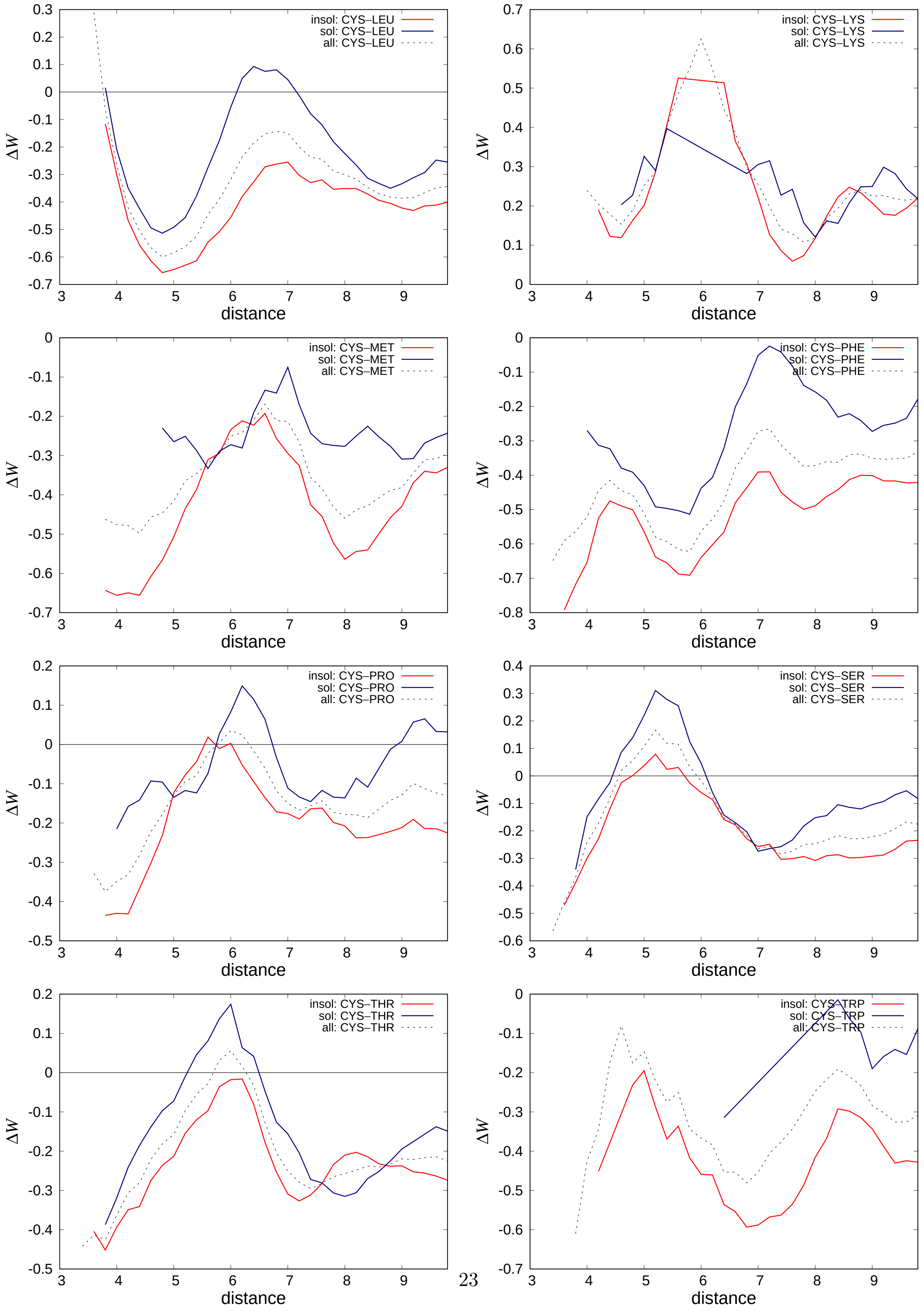
insol: CYS–LEU
sol: CYS–LEU
all: CYS–LEU
insol: CYS–LYS
sol: CYS–LYS
all: CYS–LYS
insol: CYS–MET
sol: CYS–MET
all: CYS–MET
insol: CYS–PHE
sol: CYS–PHE
all: CYS–PHE
insol: CYS–PRO
sol: CYS–PRO
all: CYS–PRO
insol: CYS–SER
sol: CYS–SER
all: CYS–SER
insol: CYS–THR
sol: CYS–THR
all: CYS–THR
insol: CYS–TRP
sol: CYS–TRP
all: CYS–TRP
ΔW
distance

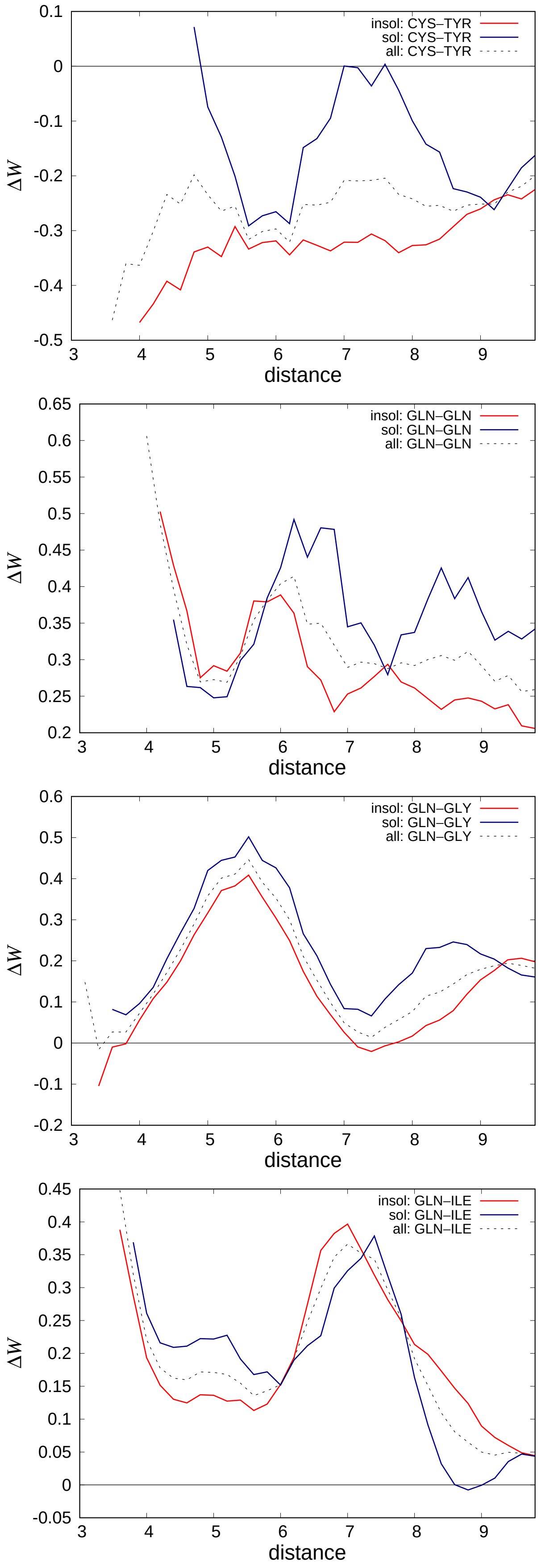

insol: CYS–TYR
sol: CYS–TYR
all: CYS–TYR
insol: GLN–GLN
sol: GLN–GLN
all: GLN–GLN
insol: GLN–GLY
sol: GLN–GLY
all: GLN–GLY
insol: GLN–ILE
sol: GLN–ILE
all: GLN–ILE
ΔW
distance

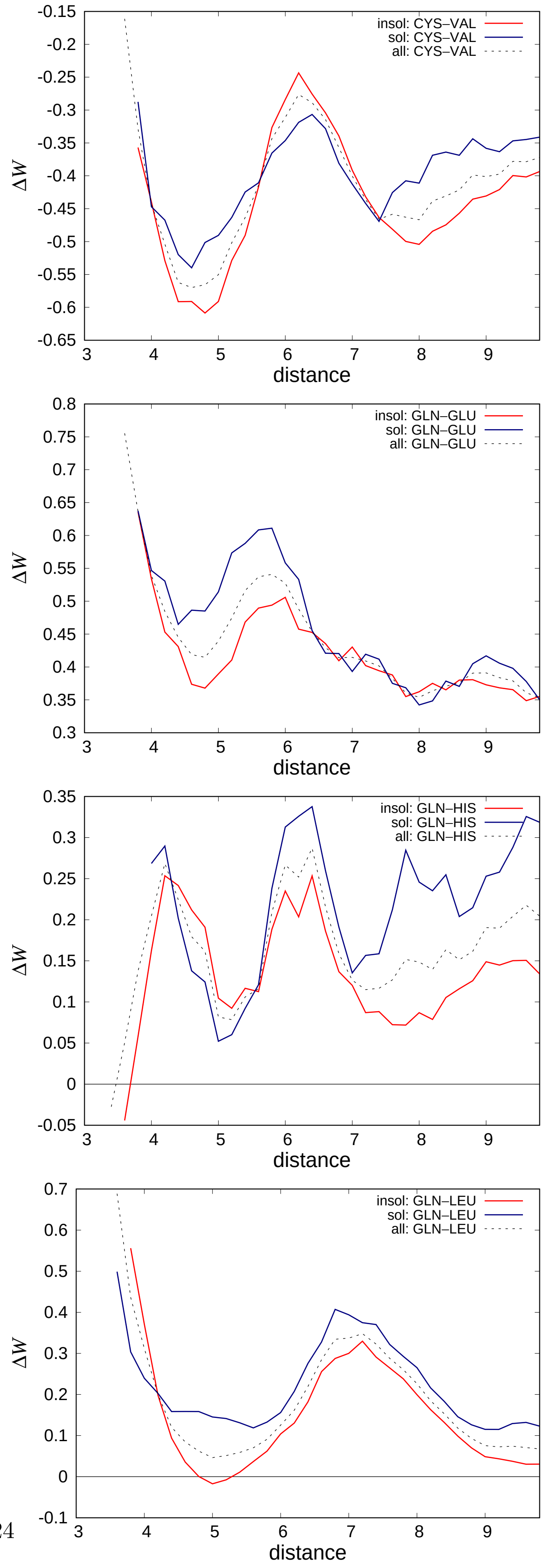

insol: CYS–VAL
sol: CYS–VAL
all: CYS–VAL
insol: GLN–GLU
sol: GLN–GLU
all: GLN–GLU
insol: GLN–HIS
sol: GLN–HIS
all: GLN–HIS
insol: GLN–LEU
sol: GLN–LEU
all: GLN–LEU
ΔW
distance

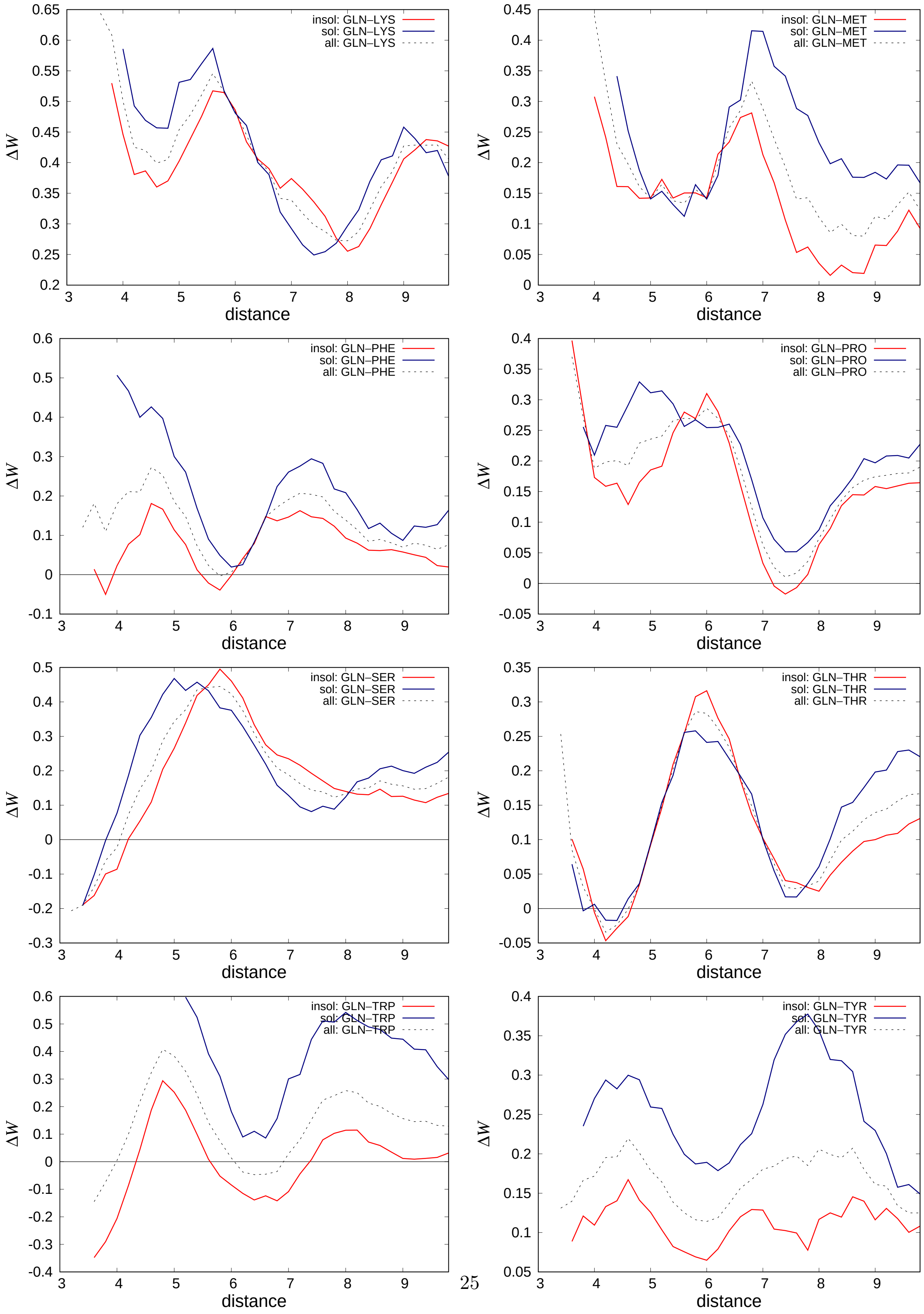
insol: GLN–LYS
sol: GLN–LYS
all: GLN–LYS
insol: GLN–MET
sol: GLN–MET
all: GLN–MET
insol: GLN–PHE
sol: GLN–PHE
all: GLN–PHE
insol: GLN–PRO
sol: GLN–PRO
all: GLN–PRO
insol: GLN–SER
sol: GLN–SER
all: GLN–SER
insol: GLN–THR
sol: GLN–THR
all: GLN–THR
insol: GLN–TRP
sol: GLN–TRP
all: GLN–TRP
insol: GLN–TYR
sol: GLN–TYR
all: GLN–TYR
ΔW
distance

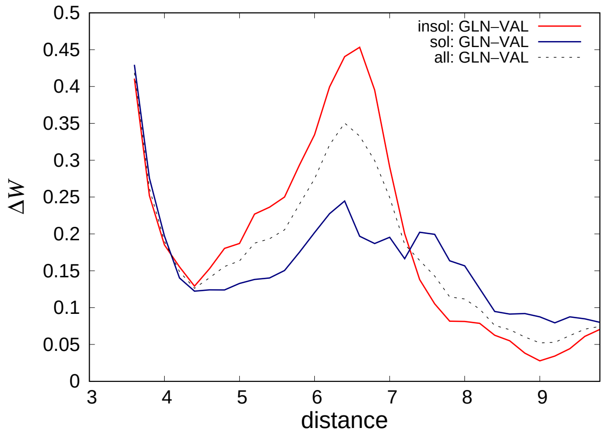
insol: GLN–VAL
sol: GLN–VAL
all: GLN–VAL
ΔW
distance
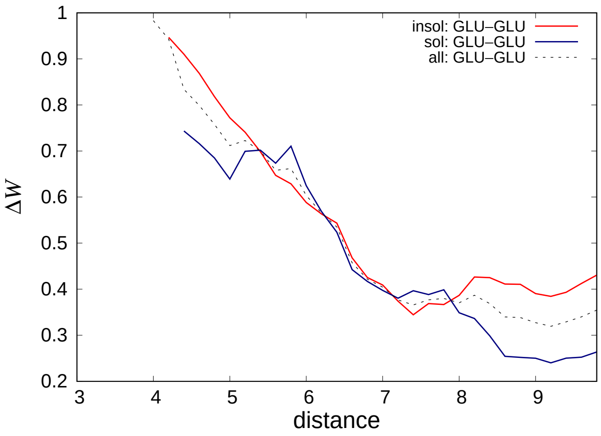
insol: GLU–GLU
sol: GLU–GLU
all: GLU–GLU
ΔW
distance
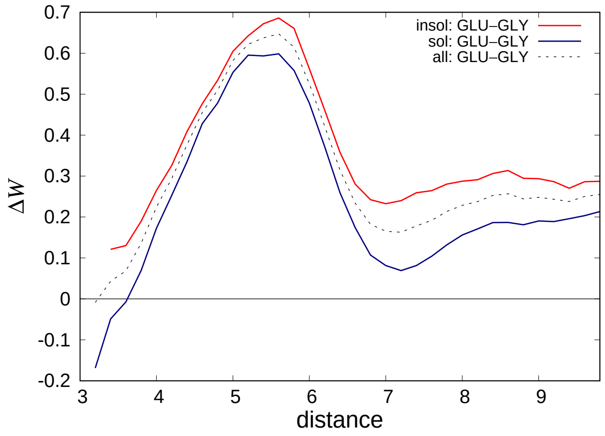
insol: GLU–GLY
sol: GLU–GLY
all: GLU–GLY
ΔW
distance
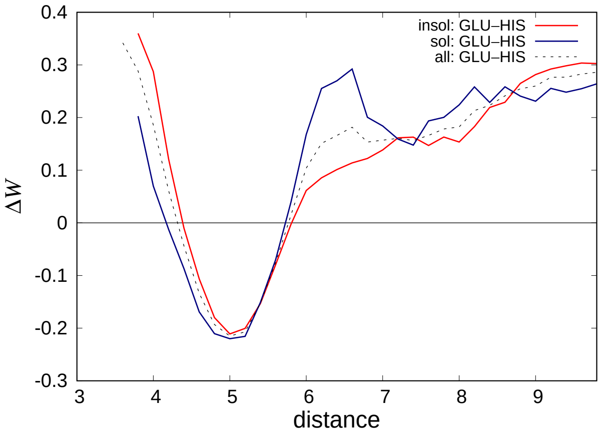
insol: GLU–HIS
sol: GLU–HIS
all: GLU–HIS
ΔW
distance
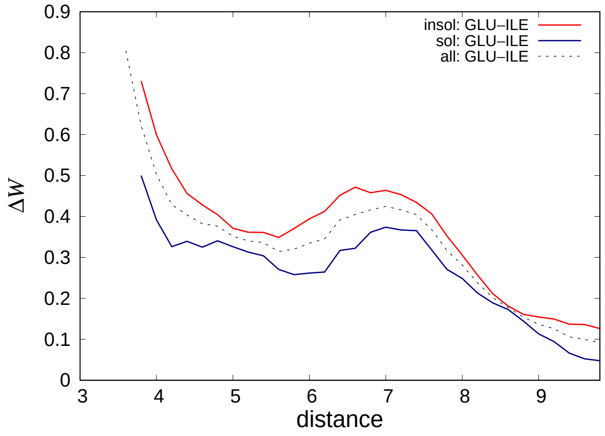
insol: GLU–ILE
sol: GLU–ILE
all: GLU–ILE
ΔW
distance
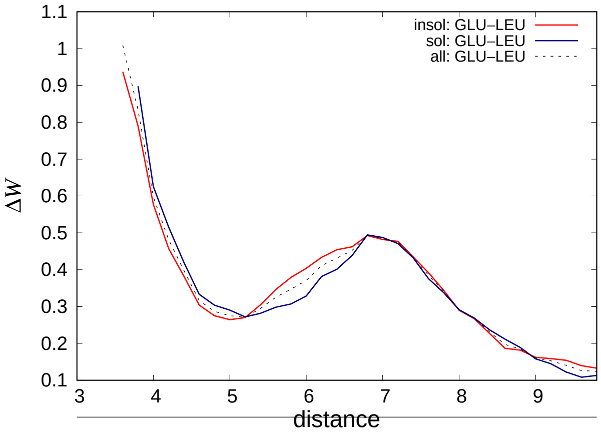
insol: GLU–LEU
sol: GLU–LEU
all: GLU–LEU
ΔW
distance
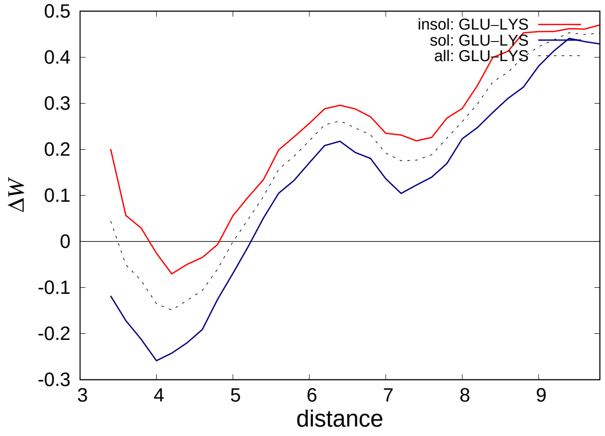
insol: GLU–LYS
sol: GLU–LYS
all: GLU–LYS
ΔW
distance
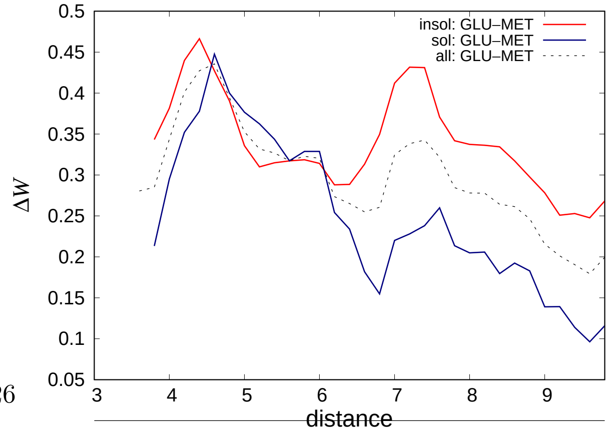
insol: GLU–MET
sol: GLU–MET
all: GLU–MET
ΔW
distance

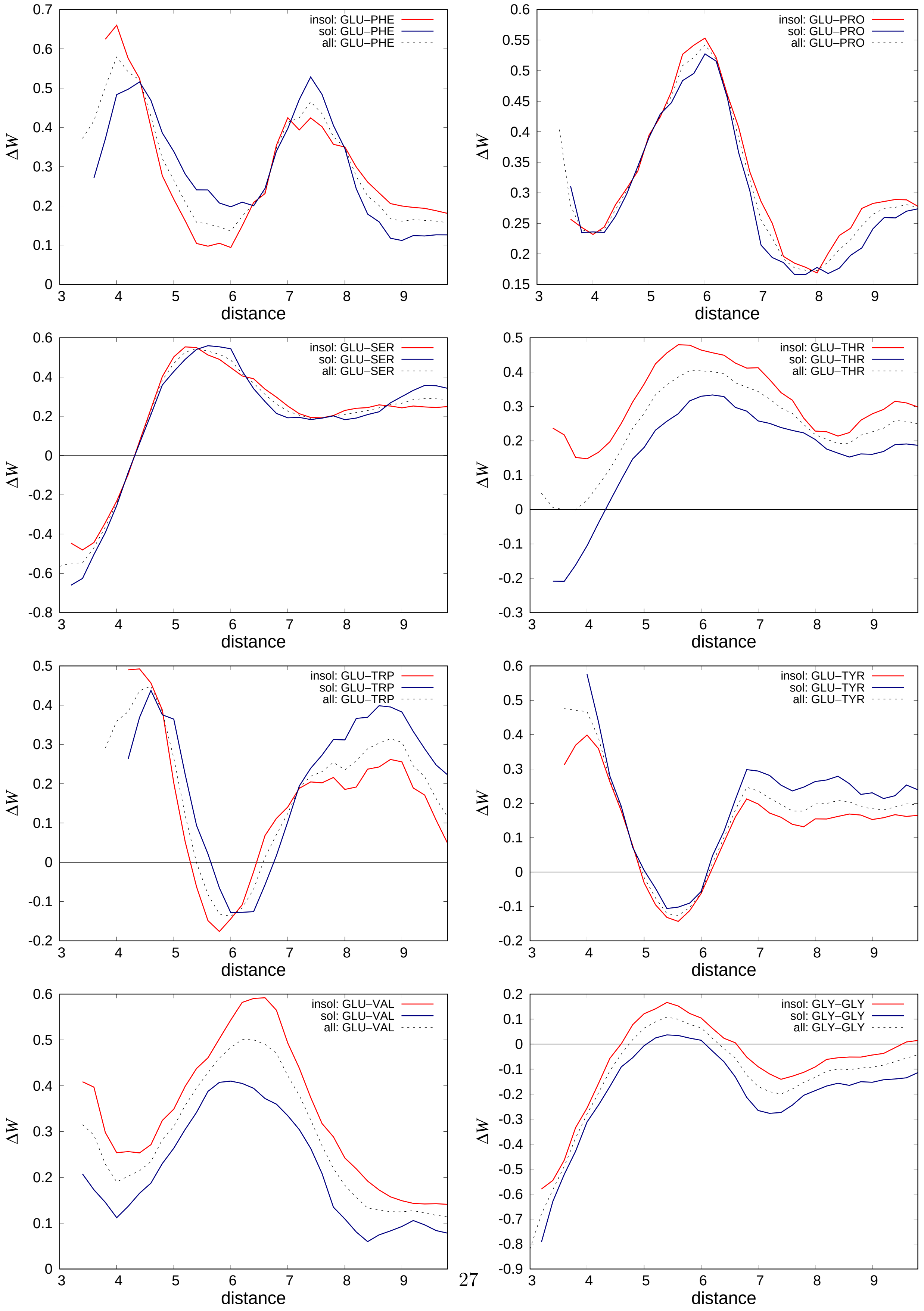
insol: GLU–PHE
sol: GLU–PHE
all: GLU–PHE
insol: GLU–PRO
sol: GLU–PRO
all: GLU–PRO
insol: GLU–SER
sol: GLU–SER
all: GLU–SER
insol: GLU–THR
sol: GLU–THR
all: GLU–THR
insol: GLU–TRP
sol: GLU–TRP
all: GLU–TRP
insol: GLU–TYR
sol: GLU–TYR
all: GLU–TYR
insol: GLU–VAL
sol: GLU–VAL
all: GLU–VAL
insol: GLY–GLY
sol: GLY–GLY
all: GLY–GLY
ΔW
distance

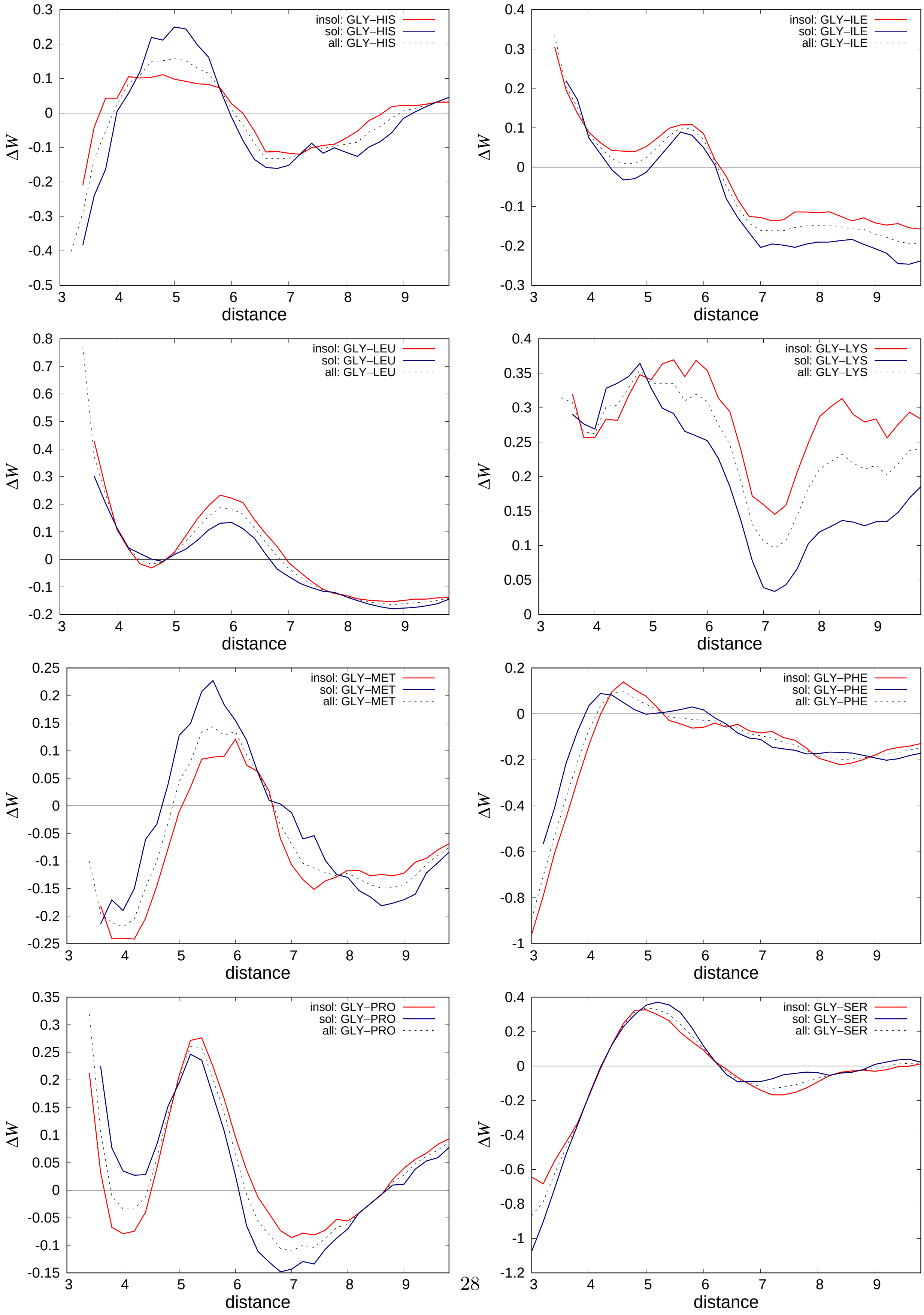
insol: GLY–HIS
sol: GLY–HIS
all: GLY–HIS
insol: GLY–ILE
sol: GLY–ILE
all: GLY–ILE
insol: GLY–LEU
sol: GLY–LEU
all: GLY–LEU
insol: GLY–LYS
sol: GLY–LYS
all: GLY–LYS
insol: GLY–MET
sol: GLY–MET
all: GLY–MET
insol: GLY–PHE
sol: GLY–PHE
all: GLY–PHE
insol: GLY–PRO
sol: GLY–PRO
all: GLY–PRO
insol: GLY–SER
sol: GLY–SER
all: GLY–SER
ΔW
distance

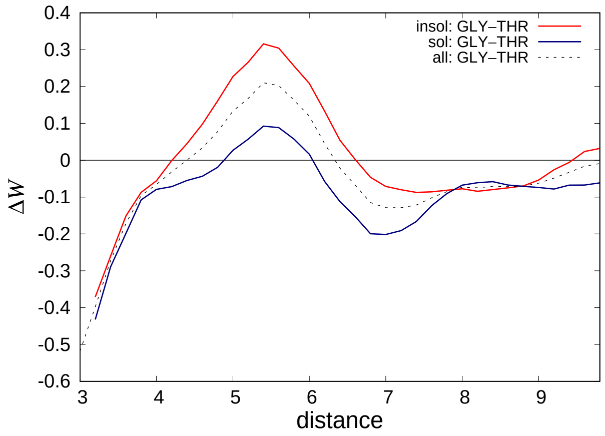
insol: GLY–THR
sol: GLY–THR
all: GLY–THR
ΔW
distance
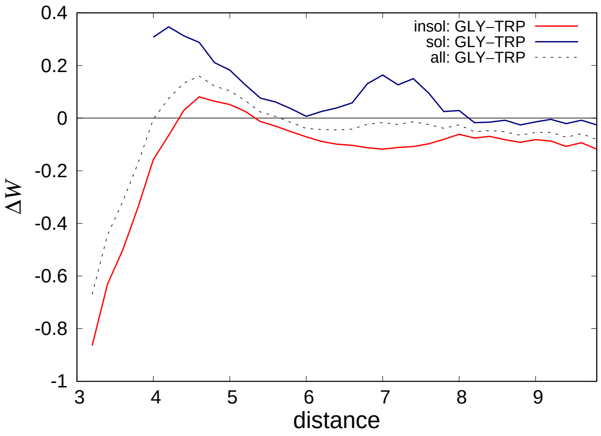
insol: GLY–TRP
sol: GLY–TRP
all: GLY–TRP
ΔW
distance
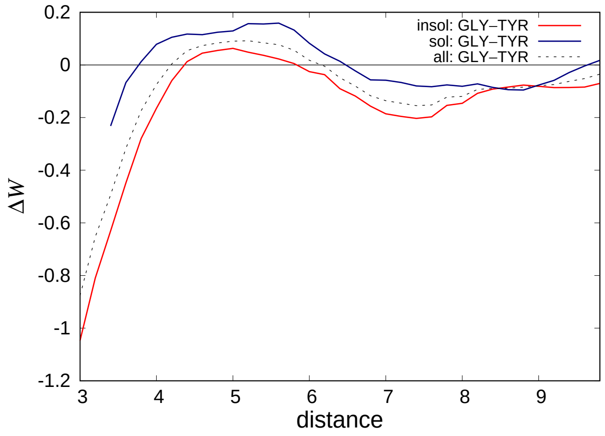
insol: GLY–TYR
sol: GLY–TYR
all: GLY–TYR
ΔW
distance
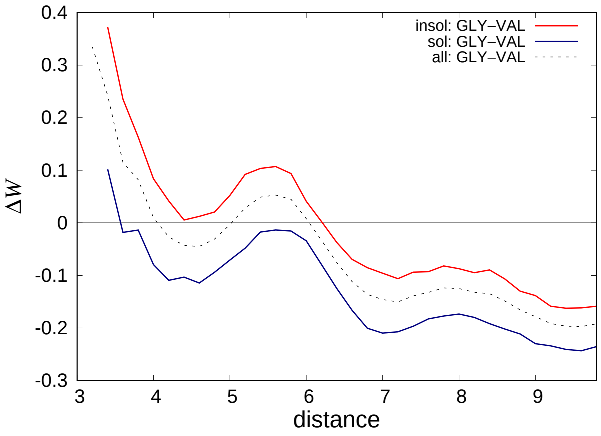
insol: GLY–VAL
sol: GLY–VAL
all: GLY–VAL
ΔW
distance
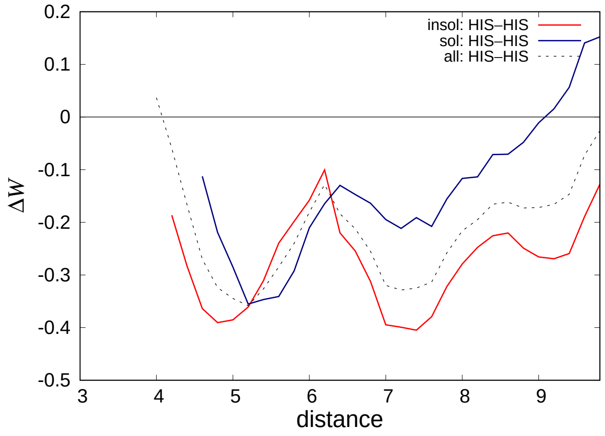
insol: HIS–HIS
sol: HIS–HIS
all: HIS–HIS
ΔW
distance
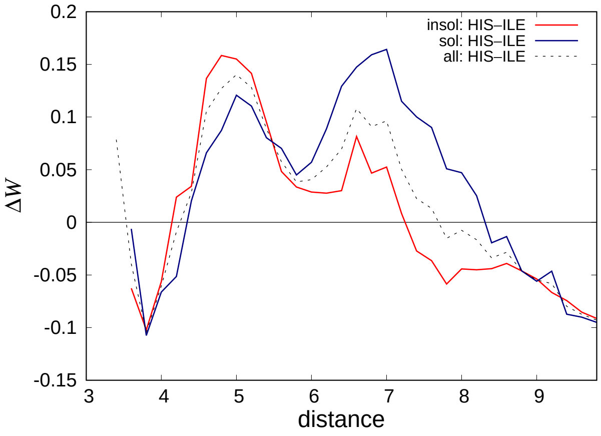
insol: HIS–ILE
sol: HIS–ILE
all: HIS–ILE
ΔW
distance
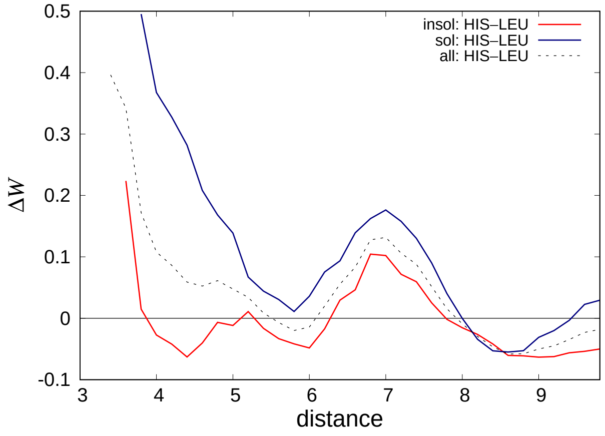
insol: HIS–LEU
sol: HIS–LEU
all: HIS–LEU
ΔW
distance
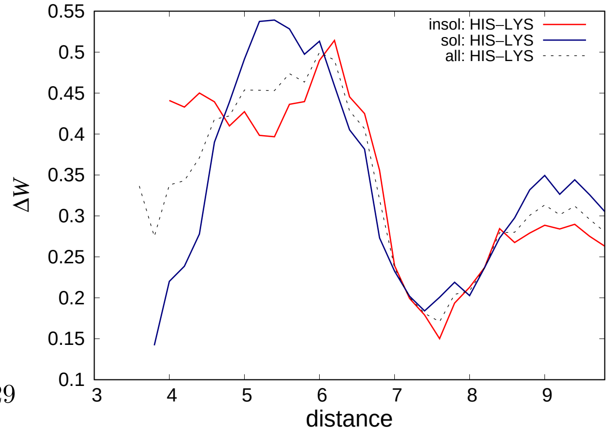
insol: HIS–LYS
sol: HIS–LYS
all: HIS–LYS
ΔW
distance

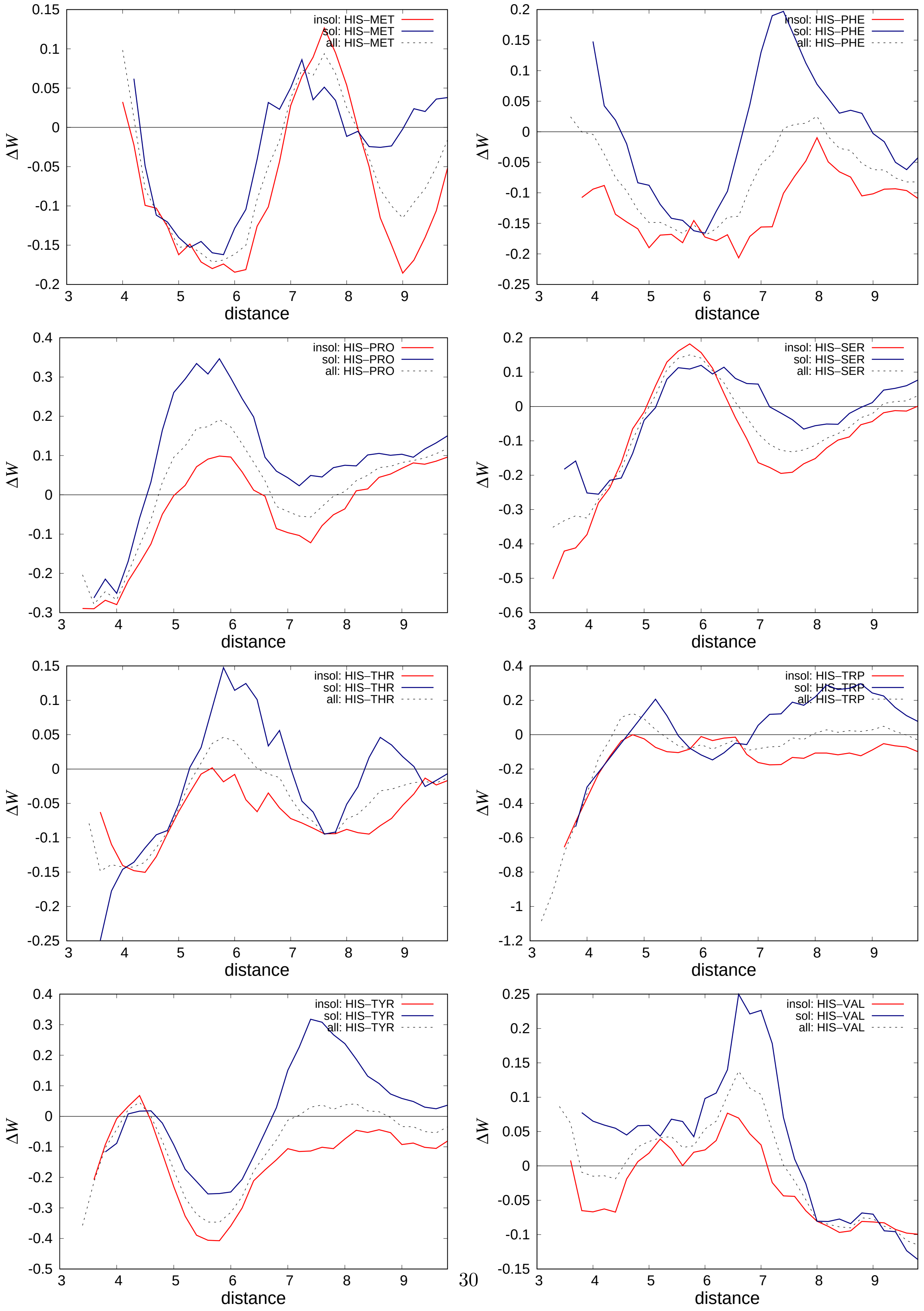
insol: HIS–MET
sol: HIS–MET
all: HIS–MET
insol: HIS–PHE
sol: HIS–PHE
all: HIS–PHE
insol: HIS–PRO
sol: HIS–PRO
all: HIS–PRO
insol: HIS–SER
sol: HIS–SER
all: HIS–SER
insol: HIS–THR
sol: HIS–THR
all: HIS–THR
insol: HIS–TRP
sol: HIS–TRP
all: HIS–TRP
insol: HIS–TYR
sol: HIS–TYR
all: HIS–TYR
insol: HIS–VAL
sol: HIS–VAL
all: HIS–VAL
ΔW
distance

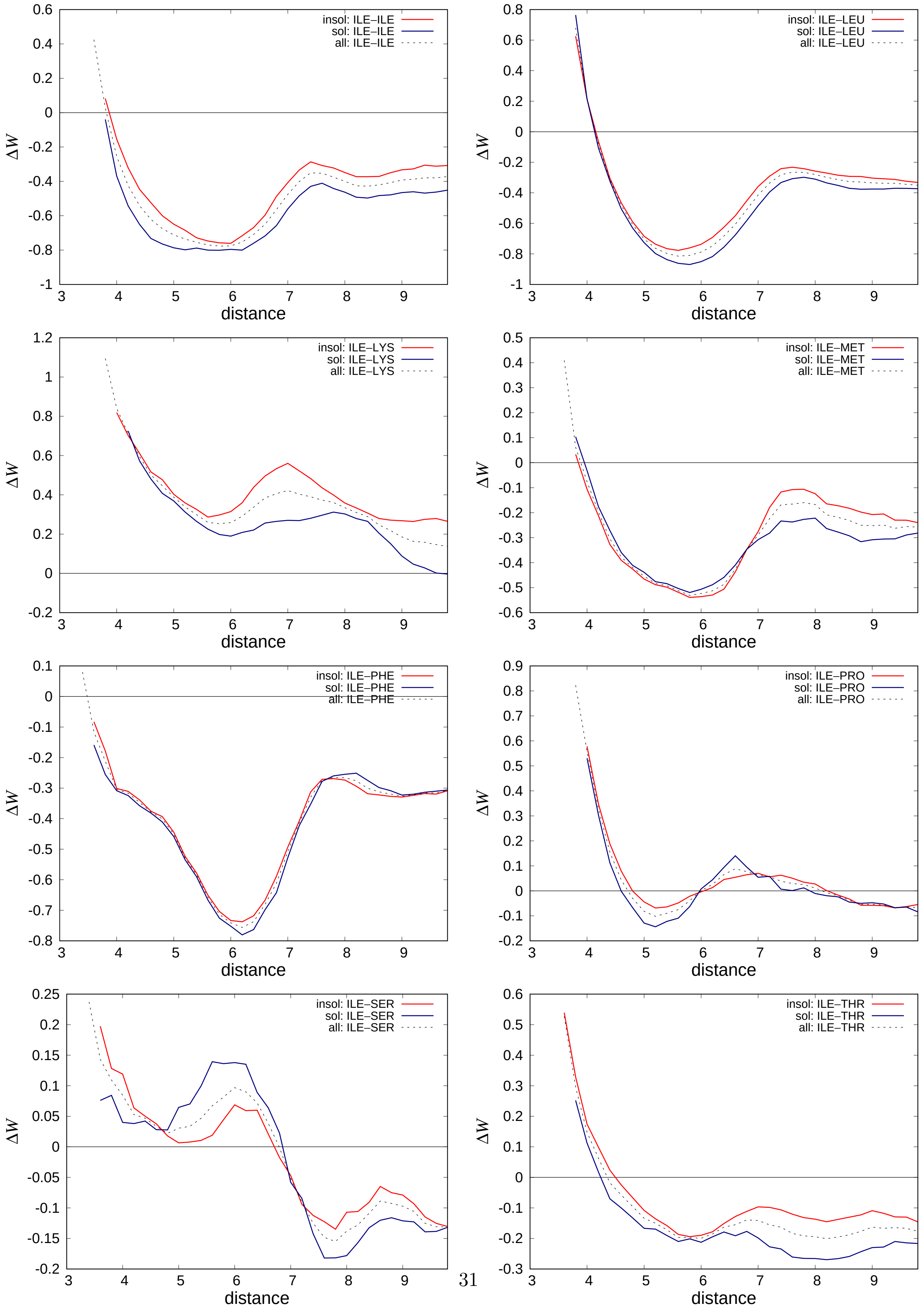
insol: ILE–ILE
sol: ILE–ILE
all: ILE–ILE
insol: ILE–LEU
sol: ILE–LEU
all: ILE–LEU
insol: ILE–LYS
sol: ILE–LYS
all: ILE–LYS
insol: ILE–MET
sol: ILE–MET
all: ILE–MET
insol: ILE–PHE
sol: ILE–PHE
all: ILE–PHE
insol: ILE–PRO
sol: ILE–PRO
all: ILE–PRO
insol: ILE–SER
sol: ILE–SER
all: ILE–SER
insol: ILE–THR
sol: ILE–THR
all: ILE–THR
ΔW
distance

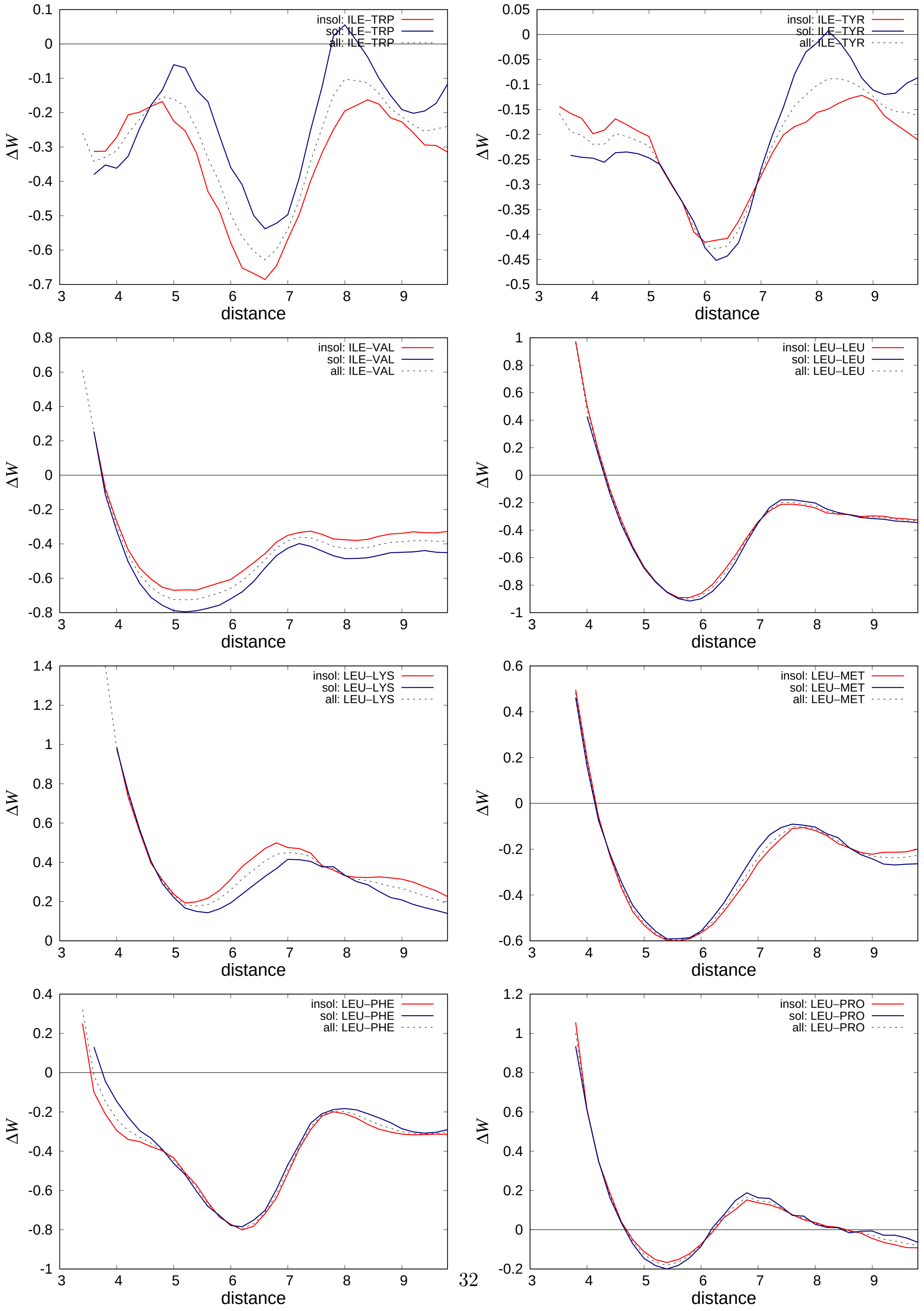
insol: ILE–TRP
sol: ILE–TRP
all: ILE–TRP
insol: ILE–TYR
sol: ILE–TYR
all: ILE–TYR
insol: ILE–VAL
sol: ILE–VAL
all: ILE–VAL
insol: LEU–LEU
sol: LEU–LEU
all: LEU–LEU
insol: LEU–LYS
sol: LEU–LYS
all: LEU–LYS
insol: LEU–MET
sol: LEU–MET
all: LEU–MET
insol: LEU–PHE
sol: LEU–PHE
all: LEU–PHE
insol: LEU–PRO
sol: LEU–PRO
all: LEU–PRO
ΔW
distance

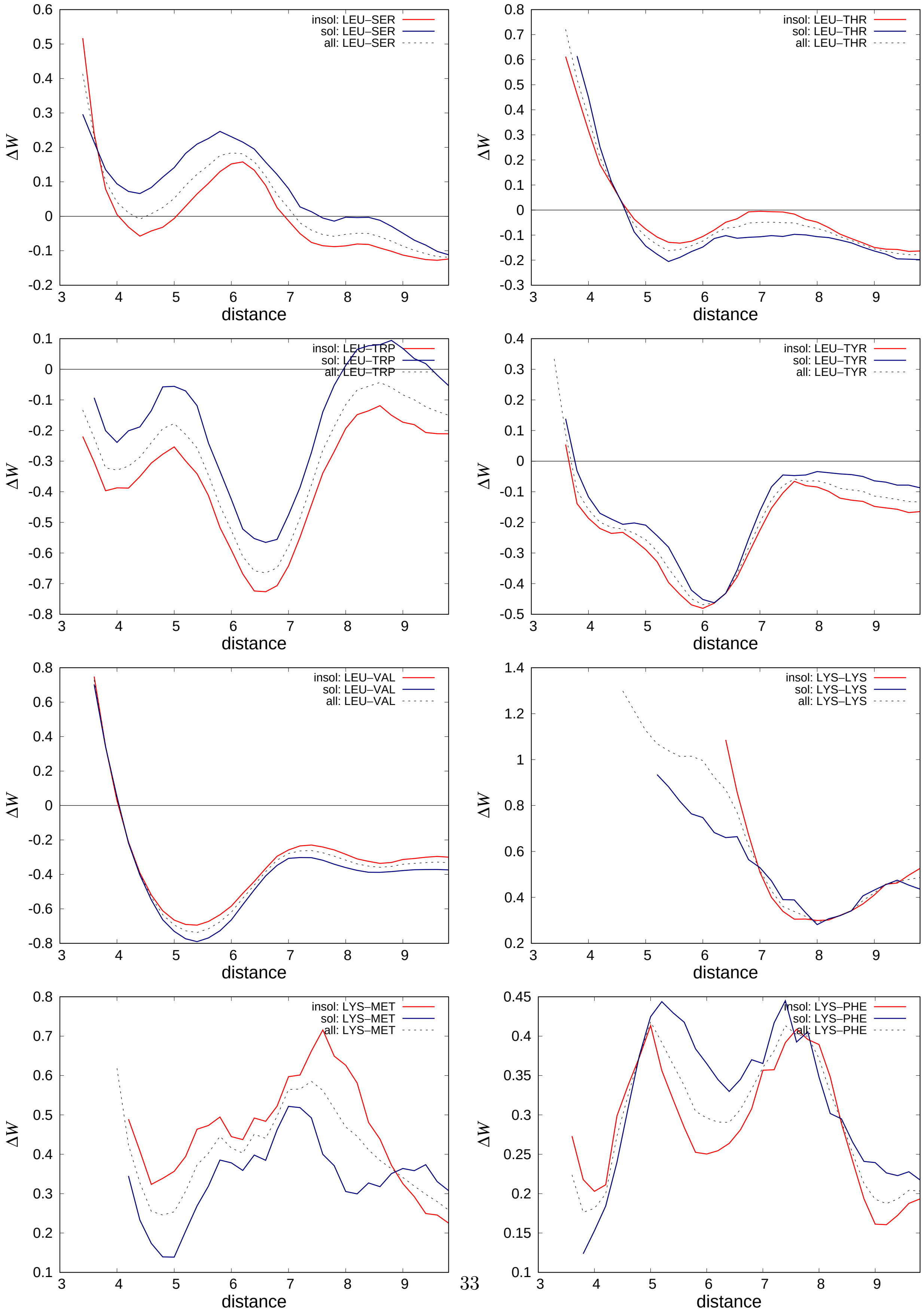
insol: LEU–SER
sol: LEU–SER
all: LEU–SER
insol: LEU–THR
sol: LEU–THR
all: LEU–THR
insol: LEU–TRP
sol: LEU–TRP
all: LEU–TRP
insol: LEU–TYR
sol: LEU–TYR
all: LEU–TYR
insol: LEU–VAL
sol: LEU–VAL
all: LEU–VAL
insol: LYS–LYS
sol: LYS–LYS
all: LYS–LYS
insol: LYS–MET
sol: LYS–MET
all: LYS–MET
insol: LYS–PHE
sol: LYS–PHE
all: LYS–PHE
ΔW
distance

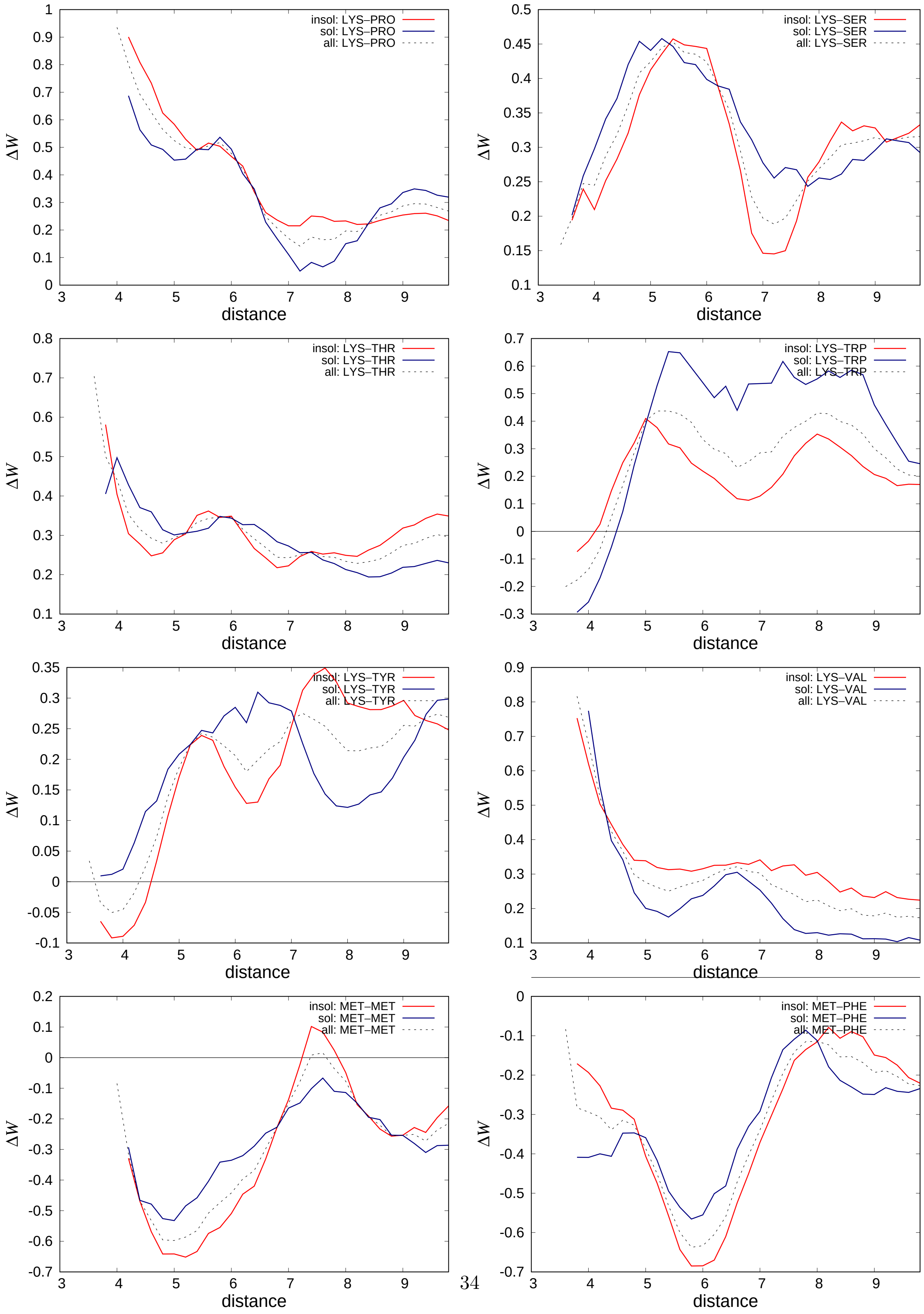
insol: LYS–PRO
sol: LYS–PRO
all: LYS–PRO
insol: LYS–SER
sol: LYS–SER
all: LYS–SER
insol: LYS–THR
sol: LYS–THR
all: LYS–THR
insol: LYS–TRP
sol: LYS–TRP
all: LYS–TRP
insol: LYS–TYR
sol: LYS–TYR
all: LYS–TYR
insol: LYS–VAL
sol: LYS–VAL
all: LYS–VAL
insol: MET–MET
sol: MET–MET
all: MET–MET
insol: MET–PHE
sol: MET–PHE
all: MET–PHE
ΔW
distance

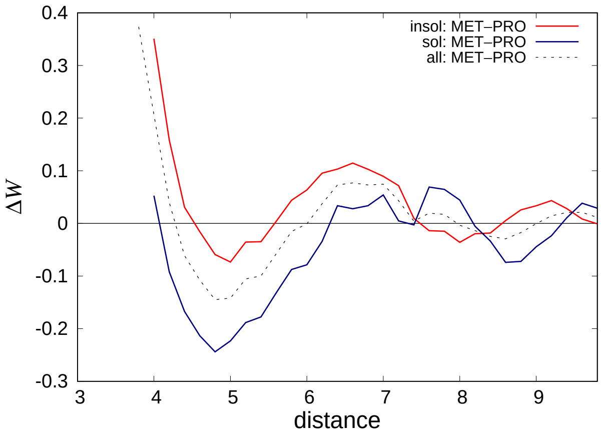
insol: MET–PRO
sol: MET–PRO
all: MET–PRO
ΔW
distance

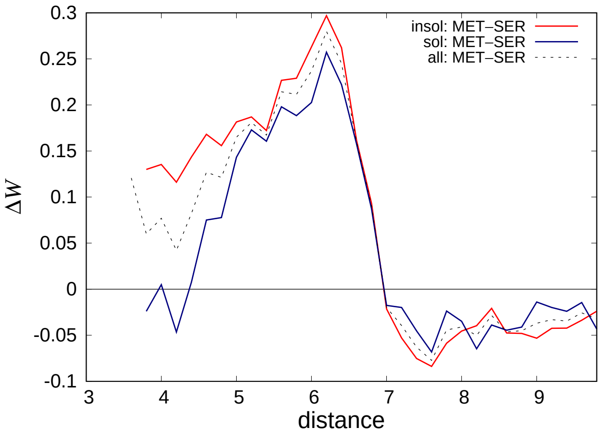
insol: MET–SER
sol: MET–SER
all: MET–SER
ΔW
distance

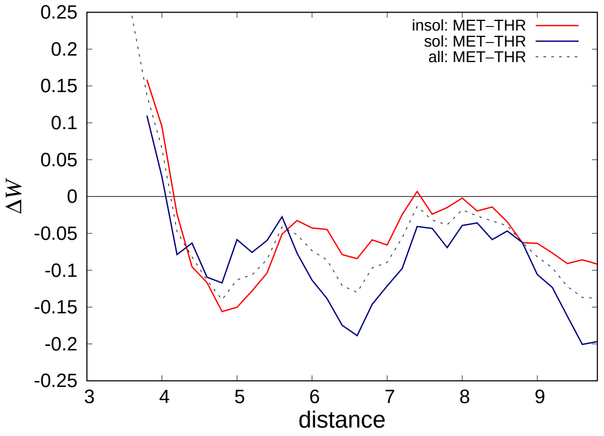
insol: MET–THR
sol: MET–THR
all: MET–THR
ΔW
distance

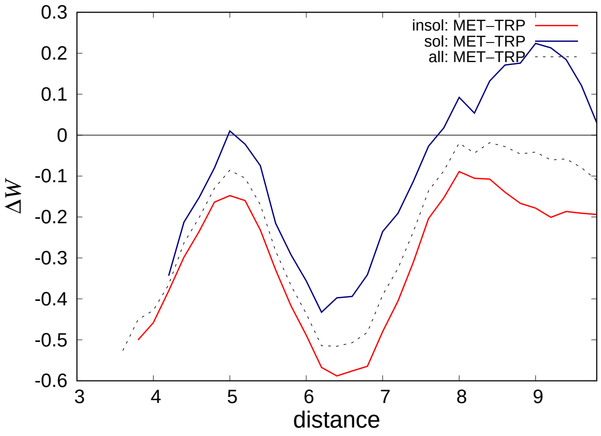
insol: MET–TRP
sol: MET–TRP
all: MET–TRP
ΔW
distance

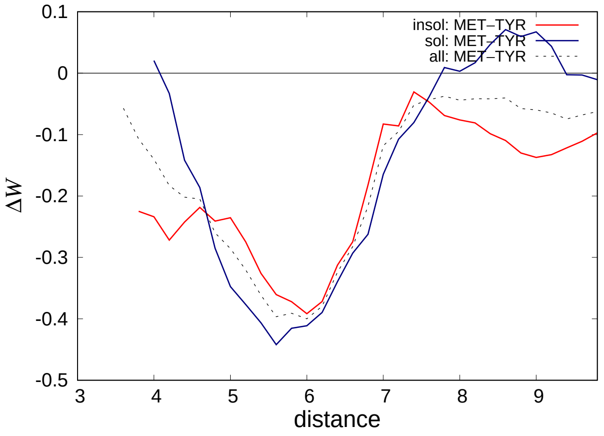
insol: MET–TYR
sol: MET–TYR
all: MET–TYR
ΔW
distance

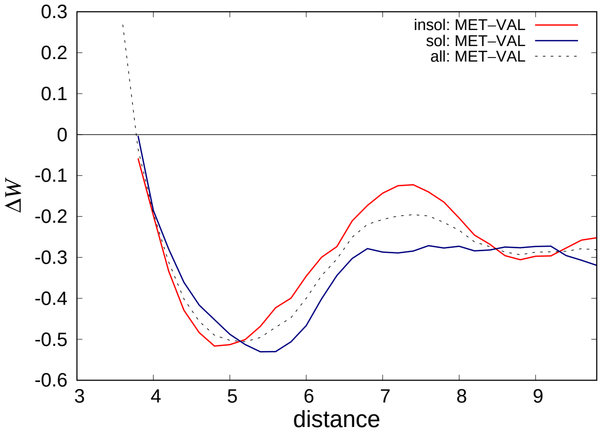
insol: MET–VAL
sol: MET–VAL
all: MET–VAL
ΔW
distance

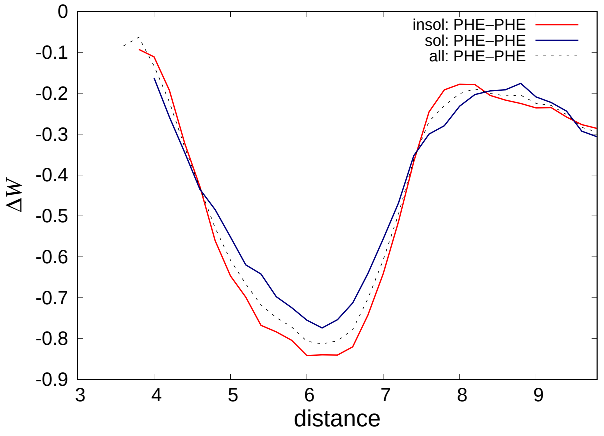
insol: PHE–PHE
sol: PHE–PHE
all: PHE–PHE
ΔW
distance

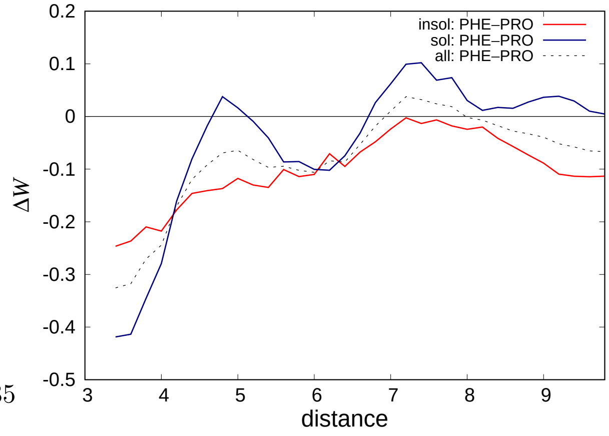
insol: PHE–PRO
sol: PHE–PRO
all: PHE–PRO
ΔW
distance

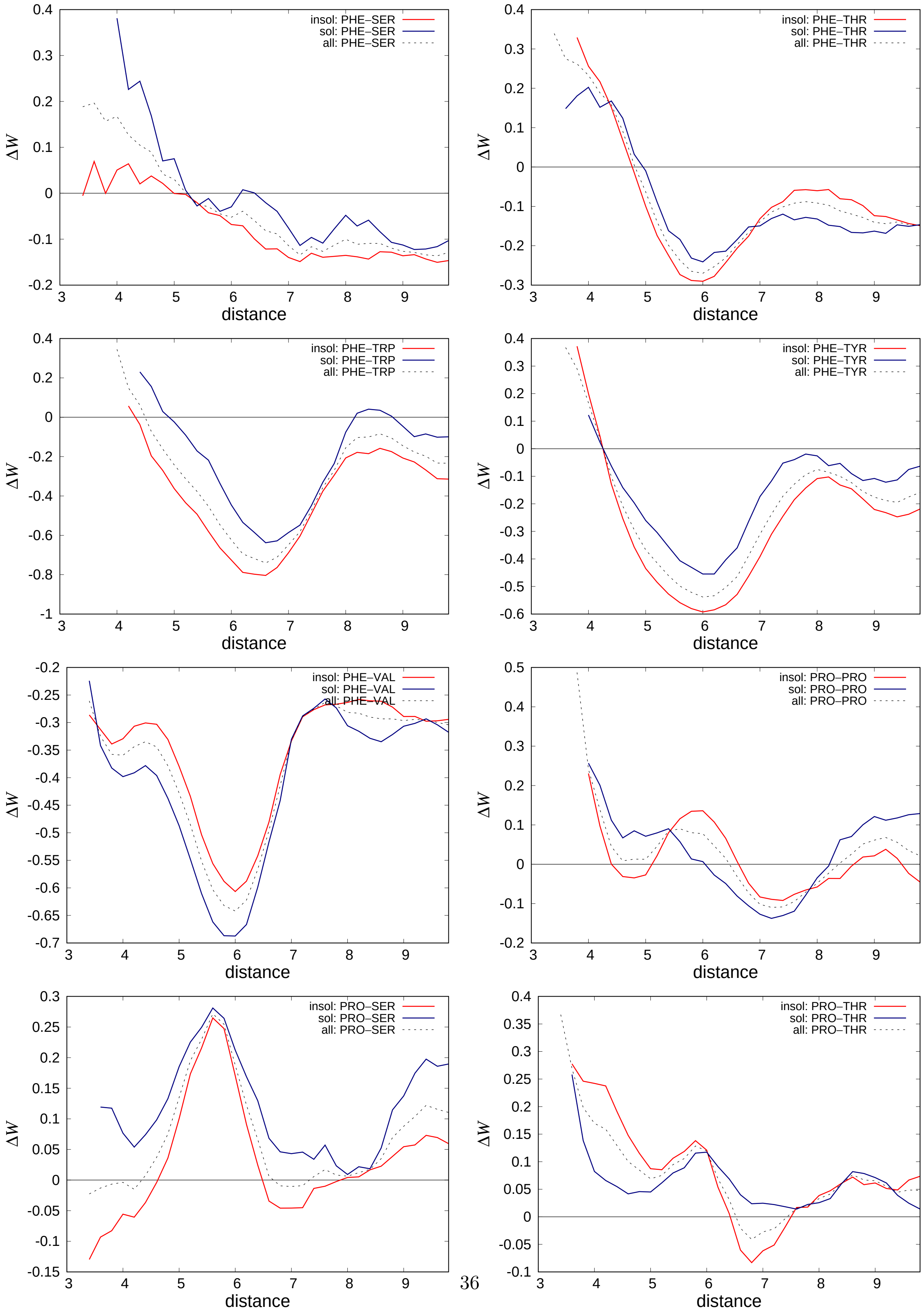
insol: PHE–SER
sol: PHE–SER
all: PHE–SER
insol: PHE–THR
sol: PHE–THR
all: PHE–THR
insol: PHE–TRP
sol: PHE–TRP
all: PHE–TRP
insol: PHE–TYR
sol: PHE–TYR
all: PHE–TYR
insol: PHE–VAL
sol: PHE–VAL
all: PHE–VAL
insol: PRO–PRO
sol: PRO–PRO
all: PRO–PRO
insol: PRO–SER
sol: PRO–SER
all: PRO–SER
insol: PRO–THR
sol: PRO–THR
all: PRO–THR
ΔW
distance

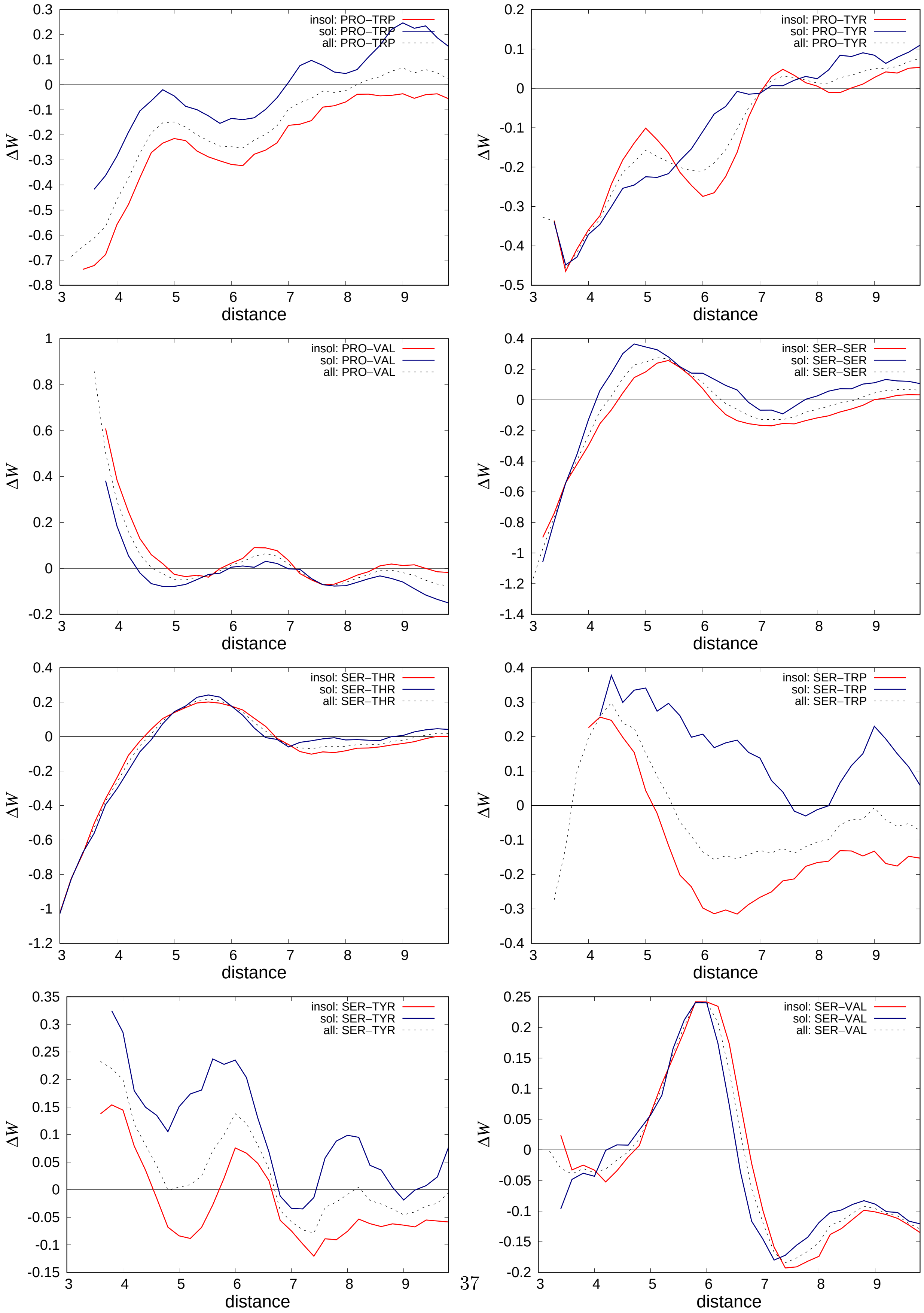
insol: PRO–TRP
sol: PRO–TRP
all: PRO–TRP
insol: PRO–TYR
sol: PRO–TYR
all: PRO–TYR
insol: PRO–VAL
sol: PRO–VAL
all: PRO–VAL
insol: SER–SER
sol: SER–SER
all: SER–SER
insol: SER–THR
sol: SER–THR
all: SER–THR
insol: SER–TRP
sol: SER–TRP
all: SER–TRP
insol: SER–TYR
sol: SER–TYR
all: SER–TYR
insol: SER–VAL
sol: SER–VAL
all: SER–VAL
ΔW
distance

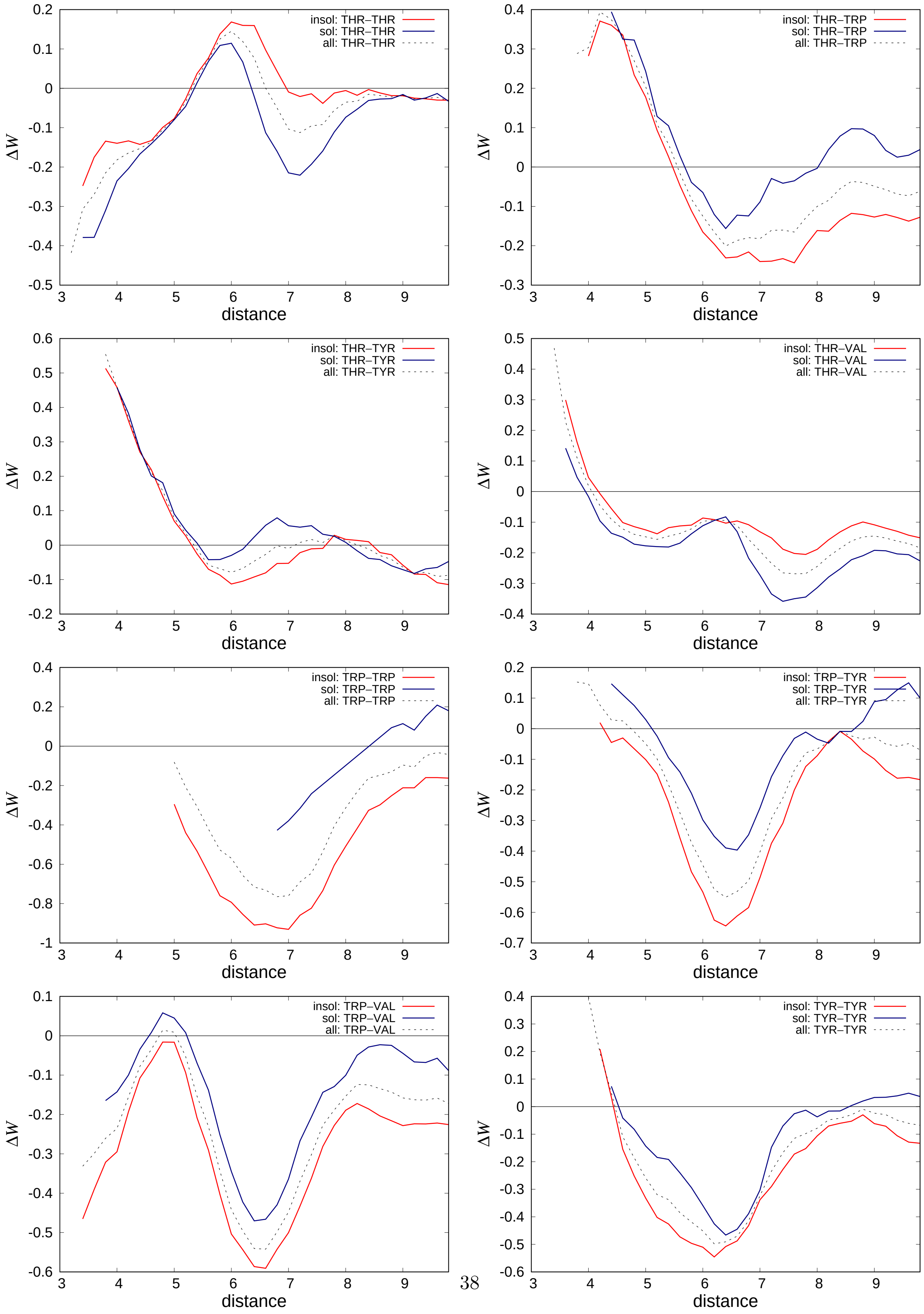
insol: THR–THR
sol: THR–THR
all: THR–THR
insol: THR–TRP
sol: THR–TRP
all: THR–TRP
insol: THR–TYR
sol: THR–TYR
all: THR–TYR
insol: THR–VAL
sol: THR–VAL
all: THR–VAL
insol: TRP–TRP
sol: TRP–TRP
all: TRP–TRP
insol: TRP–TYR
sol: TRP–TYR
all: TRP–TYR
insol: TRP–VAL
sol: TRP–VAL
all: TRP–VAL
insol: TYR–TYR
sol: TYR–TYR
all: TYR–TYR
ΔW
distance

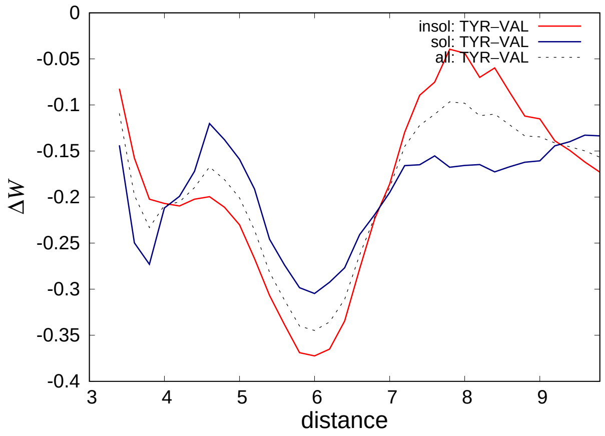
insol: TYR–VAL
sol: TYR–VAL
all: TYR–VAL
ΔW
distance

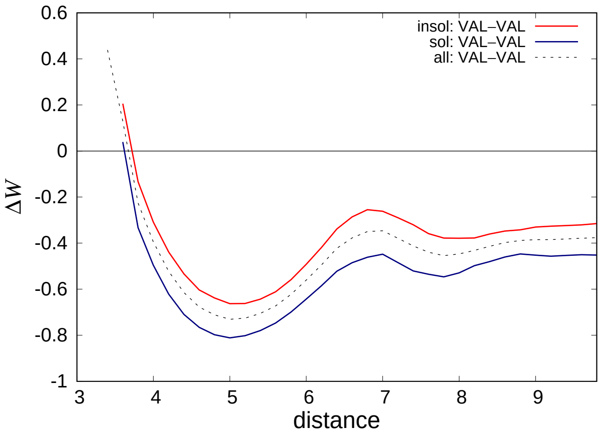
insol: VAL–VAL
sol: VAL–VAL
all: VAL–VAL
ΔW
distance

Figure S4. Residue-residue group potentials derived from datasets of highly soluble proteins ($\mathcal{S} \geq 70\%$; dashed blue line), soluble proteins ($\mathcal{S} \geq 64\%$; blue line), aggregation-prone proteins ($\mathcal{S} < 64\%$; red line), highly aggregation-prone proteins ($\mathcal{S} \leq 30\%$; red dashed line) and all proteins together (black dotted line). The energies are in kcal/mol, the distance d is computed between the residue side chain centroids of the smallest amino acids in the group, and the residue pairs are separated by at least 8 residues along the chain. Distance bins containing twenty occurrences or less are not drawn.

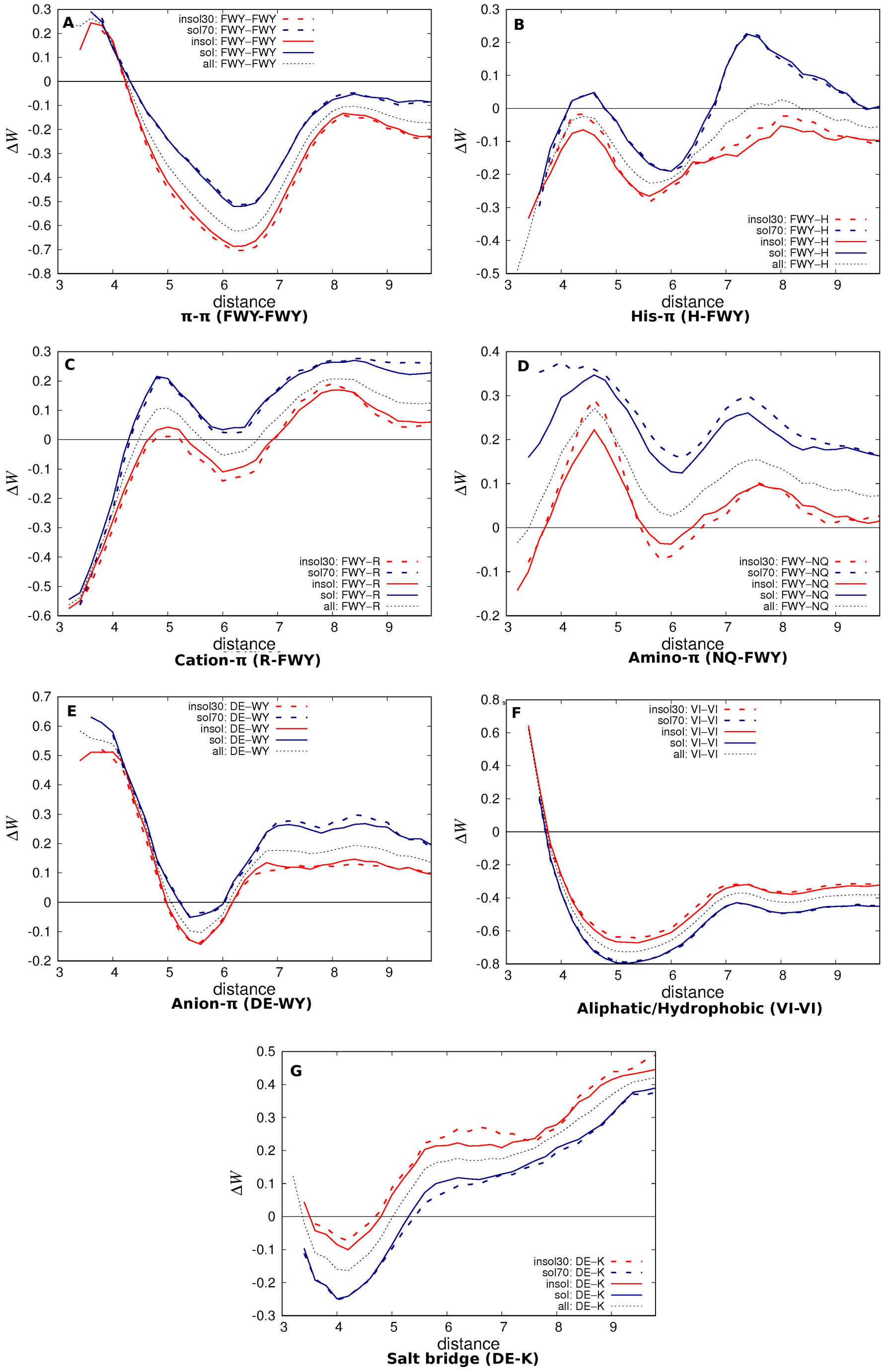

Supplement: Supplementary file 1 — Supplementary Information [file 41598_2018_32988_MOESM1_ESM.pdf]
